# Discovery of the First Highly Selective Antagonist of the GluK3 Kainate Receptor Subtype

**DOI:** 10.3390/ijms23158797

**Published:** 2022-08-08

**Authors:** Paulina Chałupnik, Alina Vialko, Darryl S. Pickering, Markus Hinkkanen, Stephanie Donbosco, Thor C. Møller, Anders A. Jensen, Birgitte Nielsen, Yasmin Bay, Anders S. Kristensen, Tommy N. Johansen, Kamil Łątka, Marek Bajda, Ewa Szymańska

**Affiliations:** 1 Department of Technology and Biotechnology of Drugs, Jagiellonian University Medical College in Kraków, 30-688 Kraków, Poland; 2 Department of Drug Design and Pharmacology, Faculty of Health and Medical Sciences, University of Copenhagen, 2100 Copenhagen, Denmark; 3 Department of Physicochemical Drug Analysis, Jagiellonian University Medical College in Kraków, 30-688 Kraków, Poland

**Keywords:** glutamate receptors, kainate receptors, subunit selectivity

## Abstract

Kainate receptors belong to the family of glutamate receptors ion channels, which are responsible for the majority of rapid excitatory synaptic transmission in the central nervous system. The therapeutic potential of kainate receptors is still poorly understood, which is also due to the lack of potent and subunit-selective pharmacological tools. In search of selective ligands for the GluK3 kainate receptor subtype, a series of quinoxaline-2,3-dione analogues was synthesized and pharmacologically characterized at selected recombinant ionotropic glutamate receptors. Among them, compound **28** was found to be a competitive GluK3 antagonist with submicromolar affinity and unprecedented high binding selectivity, showing a 400-fold preference for GluK3 over other homomeric receptors GluK1, GluK2, GluK5 and GluA2. Furthermore, in functional assays performed for selected metabotropic glutamate receptor subtypes, **28** did not show agonist or antagonist activity. The molecular determinants underlying the observed affinity profile of **28** were analyzed using molecular docking and molecular dynamics simulations performed for individual GluK1 and GluK3 ligand-binding domains.

## 1. Introduction

Ionotropic receptors for (*S*)-glutamate (iGluR), the main excitatory neurotransmitter in the mammalian central nervous system (CNS), are associated with Na^+^, K^+^ and Ca^2+^ ion channels and are responsible for the majority of rapid excitatory neurotransmission in the CNS. The iGluR family comprises four functional classes, including N-methyl-D-aspartate receptors (NMDAR), α-amino-3-hydroxy-5-methyl-4-isoxazolepropionic acid receptors (AMPAR), kainate receptors (KAR) and delta receptors. As with other iGluRs, kainate receptors function as tetramers (dimers of dimers), which are composed of individual subunits GluK1 to GluK5. Based on the affinity of kainic acid, the KAR subfamily is divided into low-affinity subunits GluK1–3, which can form functional homomeric or heteromeric ion channels, and high-affinity subunits GluK4–5 that form functional receptors only in combination with subunits GluK1–3 [1,2,3]. KARs are highly expressed in the CNS, particularly in the hippocampal formation, lateral amygdala, dorsal root ganglia, bipolar cells of the retina, cerebral cortex, and cerebellum. GluK2/GluK5 receptors represent a major population of KARs in the brain, while the expression of other subunits varies according to location, cell types, synapses, and developmental stages; for example, GluK3 is expressed primarily in the dentate gyrus in the hippocampus as well as neocortex regions [1,4,5,6,7,8].

Compared to NMDARs and AMPARs, the kainate receptor subfamily is the least explored group of iGluRs in terms of both function and mechanism of action. Unlike other iGluRs, which are located mainly postsynaptically, kainate receptors are found in both the post- and presynaptic regions. Postsynaptic KARs contribute to excitatory neurotransmission, while presynaptically localized receptors are believed to have modulatory function for both GABA and glutamate release [3,4,5,9]. Recent studies confirm that in addition to ionotropic action, both presynaptic and postsynaptic KARs can also activate ‘non-canonical’ metabotropic signaling pathways through G protein-dependent or independent mechanisms [4,10,11,12,13]. The regulation role of presynaptic KARs in glutamate release was intensively studied for hippocampal mossy fiber synapses, projecting from the dentate gyrus to CA3 pyramidal cells (MF-CA3 synapses), where KARs contribute to short- and long-term synaptic plasticity [4,5,6,13,14]. It has been suggested that in mossy fiber synapses, GluK2 and GluK3 are essential subunits of presynaptic kainate autoreceptors and most likely form GluK2/3 heteromers that facilitate synaptic plasticity and display low sensitivity to glutamate.

Various KAR subunits have been implicated in different neurological and psychiatric disorders, e.g., temporal lobe epilepsy, pain, mental retardation, migraine, schizophrenia, depression, anxiety, and bipolar disorder [4,7,15,16,17,18]. However, compared to other KARs, the role of the GluK3 subunit in physiological and pathophysiological conditions is still poorly understood. Recent observations seem to suggest that dysfunction of the GluK3-containing receptors may be involved in major depressive disorder [4,7,17,19], schizophrenia [4,7,17,20,21], and pain transmission [22].

Understanding the physiological and pharmacological potential of kainate receptors has also been slowed by the lack of highly selective pharmacological tools. Because of the high homology of orthosteric binding site regions observed among the AMPAR and KAR subunits, most of the competitive KAR ligands interact with both receptor subfamilies, and only a few compounds can discriminate between individual receptor subtypes. For this reason, little progress has been made in the clinical development of KAR-selective agents, and so far, potent antagonists with high KAR subtype selectivity have been described only for the GluK1 subunit [23,24,25,26,27]. Very recently, (*S*)-2-mercaptohistidine, a competitive micromolar GluK3 antagonist, has been reported to show at least 15–100-fold binding preference at GluK3 over the GluK1, GluK2 and GluK5 subtypes [28].

**Figure 1:**
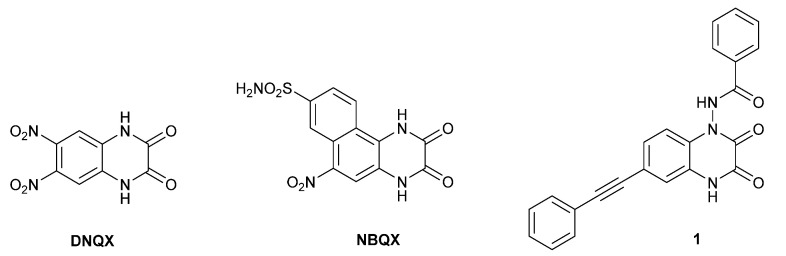
Structures of selected AMPA/kainate receptor antagonists based on the quinoxaline-2,3-dione core.

Quinoxaline-2,3-diones are one of the most important chemical classes of competitive iGluR antagonists. The early generation of quinoxalinediones such as DNQX or NBQX (Figure 1) has been reported in the 1980s and 1990s as potent AMPAR/KAR antagonists [29,30]. In the last two decades of the twentieth century, the structure–activity relationship was intensively studied in this chemical group, and many compounds built on the quinoxaline-2,3-dione core were developed in order to increase potency, selectivity, and water solubility [31]. Some of these compounds in the early stages of clinical studies demonstrated neuroprotective effects against ischemic/postischemic damage or epilepsy [16,18,19]. However, the further clinical development of these agents was stopped for various reasons.

Recently, we have published the results of studies on the new series of quinoxaline-2,3-dione derivatives and their pharmacological characterization at native and recombinant KA and AMPA receptors [32,33]. Among others, we found that aromatic amido substituents at the N1 position of the quinoxalinedione core were essential for binding to the KAR subunits GluK1 and GluK3, while a phenylethynyl moiety at the 6-position was beneficial for GluK3/GluK1 selectivity. The analogue N-(2,3-dioxo-6-(phenylethynyl)-3,4-dihydroquino-xalin-1(2*H*)-yl)benzamide (**1**, Figure 1) with micromolar affinity at GluK3 receptors, showed an approximately 30-fold binding preference for GluK3 over other KAR subtypes and an 8-fold preference over the GluA2 AMPAR subtype.

Considering **1** as a lead structure, we have developed a new series of quinoxaline-2,3-dione analogues with an arylethynyl substituent at position 6 to identify useful ligands for the functional characterization of GluK3 receptors. Here, we report the synthesis of these compounds and their evaluation at selected cloned homomeric iGluRs expressed in the *Sf9* insect cell membranes. The most active analogues were further characterized in functional assays at the GluK3 KAR subtype, as well as selected representatives of the three mGluR families. To identify the molecular determinants underlying the observed affinity profile for selected compounds, molecular docking and molecular dynamics simulations have been performed for individual GluK1 and GluK3 ligand-binding domains.

## 2. Results and Discussion

### 2.1. Chemistry

**Scheme 1:**
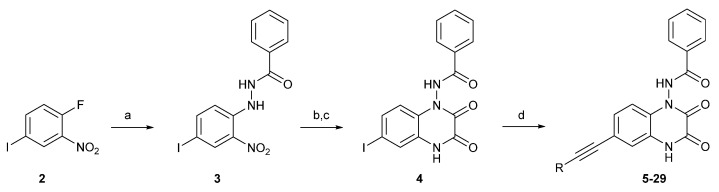
Synthesis of target compounds **5**–**29**: (**a**) benzohydrazide, K_2_CO_3_, DMSO, rt, 12 h; (**b**) ethyl chlorooxoacetate, triethylamine, THF, rt, 30 min; (**c**) iron powder, acetic acid, reflux, 30 min; (**d**) appropriate arylethynyl reagent, PdCl_2_(PPh_3_)_2_, CuI, triethylamine, DMF.

The synthesis of the target quinoxaline-2,3-diones (**5–29**) was achieved through the synthetic route presented in Scheme 1, following a modified procedure described by Pallesen for compound **1** [33]. Commercially available 1-fluoro-4-iodo-2-nitrobenzene **2** was transformed into **3** through an S_N_Ar reaction using benzohydrazide. The next step proceeded as a one-pot procedure through acylation, reduction of the nitro group, and spontaneous ring closure. The Sonogashira cross-coupling reaction allowed the introduction of a triple C-C bond moiety in place of iodine and the obtaining of the target structures (**5–29**).

### 2.2. Pharmacological Characterization

#### 2.2.1. Binding Pharmacology

The final compounds were characterized in binding studies using homomeric recombinant rat iGluRs: GluK1-GluK3, GluK5, and GluA2, expressed in *Sf9* insect cell membranes. The in vitro binding data are presented in Table 1.

The observed results suggest that the introduction of the arylethynyl moiety to the quinoxaline-2,3-dione core is strongly related to the effect of preferential binding to GluK3 receptors. Most synthesized quinoxaline-2,3-diones showed measurable affinity exclusively at GluK3 (or both GluK3 and GluK1) homomeric receptors. Among the compounds tested at the receptors GluK2, GluK5 and GluA2, all analogues except **27** and **29** were found to be inactive (*K*_i_ > 100 μM, Table 1).

Within the series obtained, compounds **5–13** were designed to explore the available steric space of the GluK3 binding site and contained an additional lipophilic substituent in the arylethynyl moiety. In general, the introduction of lipophilic groups at position 4 of the phenyl ring (**6**–**13**) resulted in a drop of GluK3 binding: the more significant, the larger the size of the substituent. Similarly, a decrease in GluK3 affinity was observed for most polar modifications (**14**–**24**), which were intended to explore the potential for hydrogen bond formation in this part of the receptor-binding pocket. Only selected substituents at the 3- or 2-position of the phenyl ring appeared to be well tolerated by GluK3, good examples of which are compounds **5**, **19** and **21**, which are equipotent or slightly more potent compared to the reference compound **1**.

Analysis of collected data, with particular emphasis on the high GluK3 potency of 2- and 4-amino derivatives **21** and **23** (*K*_i_ = 1.0 and 1.4 µM, respectively), has led to the design of the third subseries of compounds that contained a heteroaromatic moiety in the ethynyl substituent (**25**–**29**). This modification appeared to be more preferable for binding to GluK3 receptors and yielded the most interesting compounds within the entire series, **27–29**, showing 10-23-fold lower *K*_i_ at GluK3 compared to **1**. Among analogues **27–29**, compound **28** presented the most promising selectivity profile of all synthesized compounds, with submicromolar GluK3 affinity (*K*_i_ = 0.25 μM) and a preference of at least 400 times for homomeric receptors GluK3 over all other receptors tested. To our knowledge, this affinity profile makes **28** a unique compound among all KAR ligands described so far, opening new possibilities in research on the development of selective pharmacological tools for GluK3-containing kainate receptors.

The introduction of a heterocyclic substituent in the case of **27** and **29** resulted in submicromolar affinities at GluK3 receptors but with lower iGluR selectivity compared to **28**. Compound **29**, the most potent at GluK3 receptors, also showed a high binding affinity to the GluK2 and GluK1 KAR subtypes (*K*_i_ = 0.091 and 0.15 µM, respectively) as well as to the GluA2 AMPAR subtype (*K*_i_ = 0.23 µM).

#### 2.2.2. Functional Pharmacology

**Figure 2:**
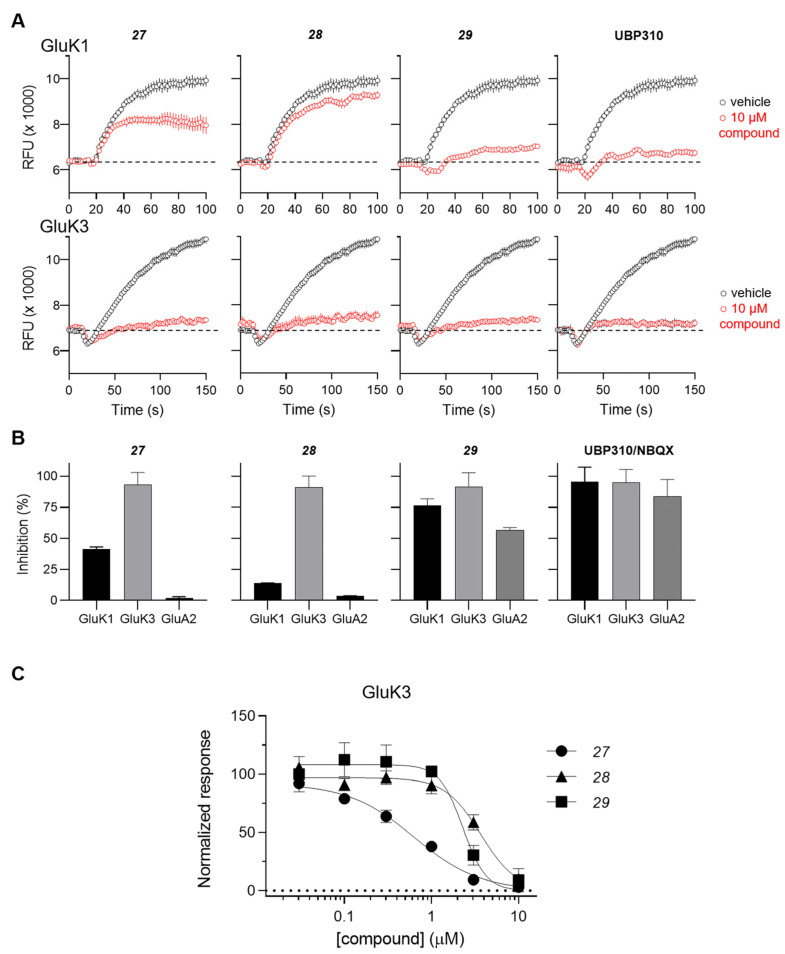
Functional testing of **27**, **28**, and **29** at GluK1, GluK3 and GluA2 receptors in a Ca^2+^ fluorescence assay. (**A**) Characterization of antagonistic activity at GluK1 and GluK3 receptors using a 96-well based [Ca^2+^] imaging assay. Representative traces from recordings of Ca^2+^ dye fluorescence from HEK293 cells expressing GluK1 (upper traces) and GluK3 (lower traces) in absence (*black*) and presence (*red*) of the test compound. At t = 16 s, agonist-induced responses are evoked by application of 0.1 mM KA. The stippled lines indicate baseline fluorescence. Traces represent mean fluorescence from four identical wells. Error bars are the SEM and are shown when larger than symbol size. (**B**) Summary of the inhibition of agonist-evoked responses produced by 10 µM concentrations of the test compounds at GluK1, GluK3, and GluA2 receptors. The inhibition by the reference antagonists UBP310 (GluK1 and GluK3) and NBQX (GluA2) are shown to the right. Data points represent the mean from at eight individual wells. Error bars are the SEM and are shown when larger than symbol size. (**C**) Concentration inhibition curves for **27**, **28**, **29** at GluK3. Data points represent the mean from at least four individual wells. Error bars are the SEM and are shown when larger than symbol size. Maximum compound test concentration was limited to 10 μM due to the solubility issues of all compounds in the FLUO buffer at room temperature.

The antagonist properties of compounds **27–29** were confirmed at the GluK3 homomeric receptor subtype in an intracellular Ca^2+^ imaging assay. As shown in Figure 2, **27**, **28** and **29** dose-dependently antagonized agonist-evoked responses at GluK3 with calculated IC_50_ values of 0.6, 3.6, and 2.2 μM, respectively.

The inhibitory activity of 10 µM concentration of compounds **27**, **28** and **29** was also tested at the GluK1 receptor and the AMPA-type GluA2 receptors. Compound **28** showed the highest level of selectivity by displaying less than 5% inhibition at GluK1 and GluA2 compared to full inhibition at GluK3 (Figure 2A,B).

Additionally, compound **28** was characterized in functional assays at selected representatives of mGluR families I, II and III: mGluR2, mGluR4 and mGluR5. In a concentration of 0.1 mM, **28** was found to be neither agonistic nor antagonistic at tested metabotropic glutamate receptor subtypes (Supplementary Materials, Figure S1).

### 2.3. Molecular Modeling

To investigate the binding mode of the new compounds and to elucidate the differences in ligand–protein interactions underlying the observed GluK3/GluK1 selectivity, molecular modeling studies have been performed. In our docking and molecular dynamics simulations, we used the available X-ray structure of the GluK1 ligand-binding domain (LBD) in complex with one of the quinoxaline-2,3-dione-based antagonists, PDB code 6SBT. As no experimental high-resolution structure of GluK3-LBD bound to a competitive antagonist to our knowledge has been resolved so far, for the purpose of this work, a homology model of GluK3-LBD was developed based on the crystal structure 6SBT (Supplementary Materials, Figure S2).

**Figure 3:**
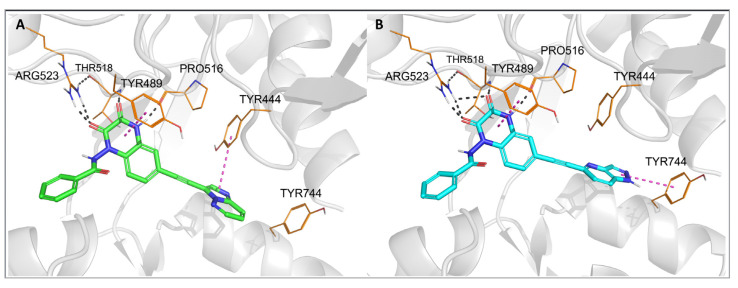
Top-ranked docking poses of **28** (**A**) and **29** (**B**) in the GluK1-LBD (PDB code: 6SBT). Hydrogen bonds are marked with gray dashes, π–π interactions are marked with pink dashes.

**Figure 4:**
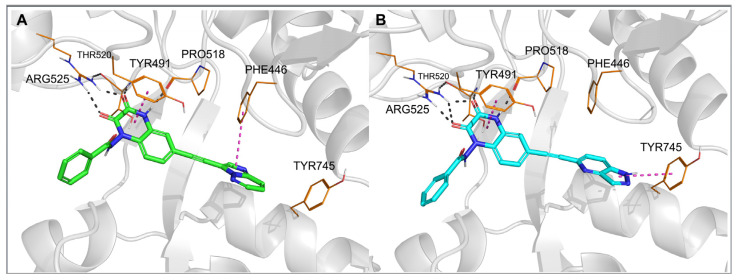
Top-ranked docking poses of **28** (**A**) and **29** (**B**) in the GluK3-LBD homology model. Hydrogen bonds are marked with gray dashes; π–π interactions are marked with pink dashes.

Flexible docking of all compounds to both the 6SBT structure and the GluK3-LBD homology model has been performed in the Schrodinger Suite environment [35]. The predominant number of compounds was successfully docked to GluK1- and GluK3-LBD, with the top-ranking docking poses adopting a similar position inside both binding sites (Supplementary Materials, Figure S3). The same characteristic pattern of interactions between the quinoxaline-2,3-dione scaffold and the D1 lobe of the receptor was observed for all ligands anchored, as in the case of other analogues in this chemical group, which were bound to the KAR or AMPAR subtypes [32,33,36]. A crucial role in these interactions is played by Arg523/525, Pro516/518, and Thr518/520 (Figure 3 and Figure 4, numbering of amino acids for GluK1 and GluK3 sequences, respectively). These amino acids are conserved among all KAR and AMPAR subunits. Additionally, the quinoxalinedione system was involved in π–π stacking with the aromatic ring of Tyr489/491. On the other hand, the benzamide moiety of the molecules was located at the border of GluK1 or GluK3 binding pockets, between the D1 and D2 lobes, and, for most of the ligands, did not form any direct interactions with the protein.

The observed GluK3-preference of the studied compounds was obviously determined by the substituent at position 6 of the quinoxaline-2,3-dione scaffold, as previously suggested [33]. In our docking results, the arylethynyl substituent of the ligands was located near Tyr444/Ph446 and Tyr744/745, showing in most of the docking poses T-shaped π-stacking interactions with at least one of these residues (Figure 3 and Figure 4). However, in the case of **28**, no direct interaction was found that could clearly discriminate between both receptors and explain the preferential binding of **28** to the GluK3 subunit.

For other X-ray structures of GluK1 antagonist complexes, 6FZ4 and 3S2V, it can be observed that the hydroxyl group of Tyr444 is involved in the water-mediated hydrogen bond network formed with the OH groups of Thr740 and Ser741 [33]. In the 4QF9 crystal structure, in turn, the alpha-amino acid moiety at position 6 of the quinoxalinedione replaces the water molecules, interacting with all the residues mentioned [37]. In the case of most of the new derivatives described herein, an arylethynyl substituent at position 6 is probably not capable of contributing to the existing H-bond network system in GluK1, but it can disrupt it, which could be an explanation for the lack of affinity at this receptor. However, it should be noted that the GluK3-binding pocket in the vicinity of the arylethynyl tail of the docked ligands is more hydrophobic due to the replacement of Tyr444 and Ser741 in GluK1 for Phe446 and Thr742 in GluK3.

**Figure 5:**
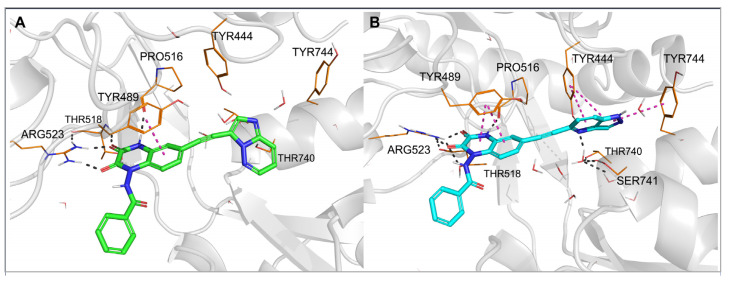
Complexes of the GluK1-LBD with compound **28** (**A**) and **29** (**B**), after 20 ns molecular dynamics simulation. Hydrogen bonds are marked with dark dashes, aromatic interactions are marked with pink dashes.

**Figure 6:**
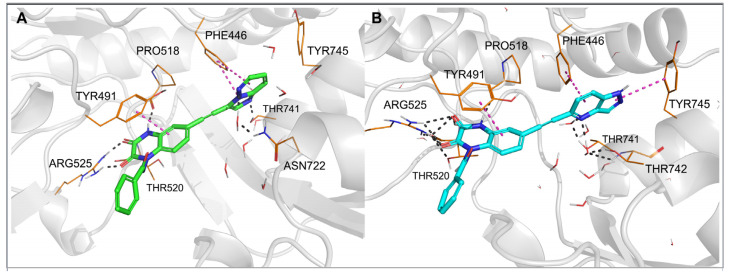
Complexes of the GluK3-LBD with compound **28** (**A**) and **29** (**B**) after 20 ns molecular dynamics simulation. Hydrogen bonds are marked with dark dashes, aromatic interactions are marked with pink dashes.

To examine in detail the molecular determinants underlying the observed affinity profile of **28** and **29**, for individual complexes of those compounds with GluK1- and GluK3-LBD, previously obtained in flexible docking, molecular dynamics (MD) simulations were conducted (Figure 5 and Figure 6; Supplementary Materials, Figures S4 and S5).

In each of the two MD simulations for **29**-GluK1-LBD, rotation of the bicyclic aromatic moiety attached to the triple bond was observed compared to the initial docking pose. This allowed the nitrogen atom at position 4 of the 1*H*-pyrazolo [4,3-*b*] pyridin-5-yl fragment to create a hydrogen bond with the hydroxyl group of Tyr444 (Figure 5B), and at the same time, it did not affect the water-mediated H-bond network arranged between Tyr444, Ser741 and Thr740. Additionally, a stable CH–π stacking between the pyridine ring and Tyr444 as well as NH–π interaction between the pyrazole ring and Tyr744 were formed. Maintenance of a hydrogen bond network, in which the N4-nitrogen atom of the pyrazolopyridinyl moiety can replace one of the water molecules, with the simultaneous formation of favorable aromatic interactions, is likely to explain the high affinity of this compound for GluK1 receptors.

Another situation could be seen for **28**, which is inactive at GluK1 but shows high potency and selectivity on the GluK3 receptor subunit. At the beginning of the MD simulation for the **28**-GluK1-LBD complex, the imidazo [1,2-*b*]pyridazin-3-yl fragment moved away from the side chain of Tyr444 and Thr740 compared to the initial docking position, allowing access of water molecules to the hydroxyl groups of these residues and reconstitution of the H-bond network (Figure 5A). Displacement of the aromatic fragment from its initial location deprived it of the beneficial CH–π stacking with Tyr444. No other direct interactions were detected between this moiety and Tyr444, Thr740 or Tyr744, either. These results suggest that the substituent at position 6 of **28** does not fit the described GluK1 binding pocket and most likely explains its lack of activity at GluK1.

In the case of the **28**-GluK3-LBD and **29**-GluK3-LBD complexes, the results of the MD simulations were consistent for both compounds. The aromatic part of the arylethynyl substituent formed a stable CH–π stacking with Phe446 (Figure 6). The more extended pyrazolopyridinyl fragment of **29** allowed the NH–π stacking with Tyr745, while **28** formed hydrophobic interactions with this residue. The N4-nitrogen atom of the heterocyclic moiety in **29** created water-mediated H-bonds with Thr741 and Thr742 (Figure 6B). The N2-nitrogen atom of the imidazopyridazinyl fragment in **28** formed a direct hydrogen bond with the -OH group of Thr741. Furthermore, the position of the heterocyclic moiety in **28** allows for the creation of a water-mediated hydrogen bond between the N4-nitrogen atom and non-conserved Asn722 (Figure 6A). This amino acid is replaced in GluK1 by Ser721, with a shorter side chain, probably preventing the formation of an analogous interaction in GluK1. Therefore, the water-mediated hydrogen bond to Asn722 may be one of the determinants of the observed GluK3-selectivity of **28** in addition to the poor fit to GluK1 described above.

## 3. Materials and Methods

### 3.1. Chemistry

All reagents were purchased from commercial suppliers and used without further purification. Melting points (mp) were determined on a MEL-TEMP II melting point apparatus (LD Inc., USA) and were uncorrected. ^1^H NMR spectra for compounds **14**, **19**, **20**, and **23** were recorded at 300 MHz on a Varian-Mercury-VX 300 MHz PFG spectrometer, while for other compounds, ^1^H NMR spectra were recorded at 500 MHz, using a 500 MHz JEOL FT-NMR spectrometer (JNM-ECZR500 RS1 version ECZR) with a solvent signal as an internal standard. All ^13^C NMR spectra were recorded at 126 Hz using a 500 MHz JEOL FT-NMR spectrometer. Dimethyl-*d*_6_-sulfoxide (DMSO-*d*_6_) was used as a solvent. Chemical shifts (δ) are expressed in parts per million (ppm). Multiplicities are depicted as the abbreviations: s—singlet, br.s—broad singlet, d—doublet, t—triplet, q—quartet, m—multiplet, dd—doublet of doublets, ddd—doublet of doublet of doublets, dt—doublet of triplets, tt—triplet of triplets, td—triplet of doublets. Mass spectra (LC/MS) were performed on a Waters ACQUITY TQ Detector mass spectrometer (electrospray ionization mode ESI–tandem quadrupole), coupled to a Waters Acquity Ultra Performance Liquid Chromatography (UPLC). The UPLC purity of all final compounds was determined (%). Elemental analyses (C, H, N) were performed on an Elemental Analyzer CHNS (Vario Micro Cube), and the obtained results were within 0.4% of the theoretical values unless stated otherwise. For column chromatography purification, a CombiFlash Rf+ apparatus (Teledyne Isco Inc., USA) was used, and the mixture of dichloromethane/methanol was applied as the mobile phase.

General procedure for the preparation of target compounds (**5**–**29**).

Compound **4** (102 mg, 0.25 mmol) was dissolved in dry DMF (1 mL), PdCl_2_(PPh_3_)_2_ (18 mg, 0.025 mmol) and CuI (5 mg, 0.25 mmol) were added, and the reaction mixture was degassed with N_2_ for 5 min. Triethylamine (1.4 mL, 10 mmol), adequate acetylene derivative (0.325 mmol) and DMF (1 mL) were added to the flask, and the mixture was stirred under nitrogen at 110 °C for 15 min to 3 h. The raw product was purified by flash column chromatography using dichloromethane and methanol as mobile phase.

All final compounds **5**–**29** were analytically characterized by ^1^H NMR, ^13^C NMR, LC/MS purity determination as well as elemental analysis. All data are shown in the Supplementary Materials (section Analytical characterization of the final compounds **5**–**29**).

### 3.2. Pharmacology

#### 3.2.1. Receptor-Binding Studies

Ligand binding affinities at recombinant rat homomeric GluA2, GluK1−3, and GluK5 were determined as previously detailed [38,39,40]. In short, ligands were diluted in assay buffer (GluA2: 50 mM Tris-HCl, 100 mM KSCN, 2.5 mM CaCl_2_ pH 7.2 at 4 °C; GluK1−3,5: 50 mM Tris-HCl pH 7.1 at 4 °C), mixed with *sf9* insect cell membranes expressing the respective receptors and radioligand (GluA2: [^3^H]-(*RS*)-AMPA (57.5 Ci/mmol; PerkinElmer, Waltham, MA, USA), GluK1: [^3^H]-(*S*)-NF608 (16.3 Ci/mmol) [38], GluK2,3,5: [isopropenyl-^3^H]-kainic acid (43.6–47.2 Ci/mmol; PerkinElmer, Waltham, MA, USA) in a total volume of 0.25 mL followed by 2 h equilibration at 4 °C. GluK1−3 assays were filtered through GF/B type glass fiber filters in microtiter plate format (UniFilter-96, PerkinElmer, Waltham, MA, USA) and washed twice with ice-cold assay buffer on a FilterMate manifold (PerkinElmer, Waltham, MA, USA). The filters were dried at 70 °C for 1 h, and 50 μL/well Microscint 20 (PerkinElmer, Waltham, MA, USA) was added. Radioactivity was detected with a TopCounter (PerkinElmer, Waltham, MA, USA). GluA2 and GluK5 assays were filtered on GF/C type filters using Millipore 12-well filtration manifolds (Merck Life Science, Søborg, Denmark) and washed twice with ice-cold assay buffer. Filters were placed in pony vials (PerkinElmer, Waltham, MA, USA), 3 mL of Ultima Gold scintillation fluid (PerkinElmer, Waltham, MA, USA) was added, and DPM (disintegrations per minute) were determined using a TriCarb 2900 scintillation counter (PerkinElmer, Waltham, MA, USA).

All competition curves (n ≥ 3 per ligand) were determined in triplicate. Data were analyzed using GraphPad Prism 6 (GraphPad Software, San Diego, CA, USA) to determine ligand affinity (*K*_i_) and Hill coefficient (*n*_H_) using the one-site *K*_i_ and four-parameter logistics equations, respectively.

#### 3.2.2. Intracellular Ca^2+^ Assay

For the determination of antagonistic activity of compounds at GluK1, GluK3 and GluA2 receptors, stable HEK293 cell lines expressing rat GluK1b(*Q*), GluK3a, and GluA2(*Q*)i were created. A polyclonal stable cell line expressing GluK3a was created as described previously [28]. For the creation of polyclonal stable cell lines expressing GluK1and GluA2 receptors, cDNA encoding blasticidin-S deaminase (blas) enzymes were inserted into the pIRES plasmid vector (Clontech, Mountain View, CA, USA) to create the plasmid vector pRES(blas). Subsequently, cDNA for rat GluK1(Q)1b and the flip isoform of rat GluA2(Q)i were inserted into pRES(blas) to create the expression constructs GluK1b(Q)pIRES(blas) and GluA2i(Q)pIRES(blas), respectively. These were used to transfect GripTite^TM^ HEK293 cells followed by selecting with 20 µg/mL blasticidin (Invivogen, Toulouse, France) and 0.50 mg/mL geneticin in the presence of 20 µM of the AMPA receptor antagonist CNQX (Tocris, Bristol, UK) until stably transfected GluK1b(Q) and GluA2(Q)i cell lines were obtained. For functional assays, cells were plated into clear-bottomed, black 96-well fluorescence culture plates (Corning, Vordingborg, Denmark), and when >70% confluent, the cells were loaded with 2 μM Fluo 4-AM calcium sensitive dye (Abcam, Cambridge, UK) and used for the imaging of agonist-evoked changes in intracellular Ca^2+^ using a FlexStation I plate reader (Molecular Devices, San Jose, CA, USA). For IC_50_ experiments, cells were pre-incubated with 50 µL FLUO buffer containing (in mM): 100 choline chloride, 5 KCl, 1 MgCl_2_, 20 CaCl_2_, 10 HEPES (pH 7.4) and increasing concentrations of test compound for 20 min at room temperature to achieve equilibrium before measurement of agonist responses. The maximum compound test concentration was limited to 10 µM due to the solubility issues of all compounds in the FLUO buffer at room temperature. Changes in dye fluorescence in response to the addition of an agonist solution were then measured at 538 nm using excitation at 485 nm and emission cut-off at 515 nm. Baseline fluorescence was measured for 16 s before the addition of 50 µL agonist solution to each well of the assay plate, and fluorescence was measured for 120 to 160 s after addition of the agonists. Peak change in fluorescence was calculated as the difference between the maximal observed increase in fluorescence and pre-agonist baseline fluorescence. The agonist solutions contained 100 µM KA together with 300 µM of the KAR positive allosteric modulator BPAM344 (Sigma-Aldrich Chemical, St. Louis, MO, USA) for GluK1 and GluK3 experiments or 100 µM glutamate together with 100 µM of the AMPAR positive allosteric modulator cyclothiazide for GluA2 experiments (HelloBio, Bristol, UK). Responses were determined in at least triplicate at each compound concentration.

#### 3.2.3. mGluR2 Functional Assay

Culture media, serum, antibiotics and buffers for cell culture were obtained from Invitrogen (Paisley, UK). The Fluo-4/AM dye was obtained from Molecular Probes (Eugene, OR, USA), and Glu was purchased from Sigma-Aldrich. The construction and basic pharmacological characterization of the stable mGlu_2_/Gqo5-HEK293 cell line has been described previously [41].

Cell culture and Ca^2+^/Fluo-4 Assay. The mGlu_2_/Gqo5-HEK293 cells were cultured in Dulbecco’s Modified Eagle Medium-Glutamax-I supplemented with 100 U/mL penicillin, 100 µg/mL streptomycin, 5% dialyzed fetal bovine serum, 100 µg/mL zeocin and 1 mg/mL G418. The cells were split into poly-D-lysine-coated black 96-well plates with clear bottoms (6 × 10^4^ cells/well). The following day, the culture medium was aspirated, and the cells were incubated in 50 µL assay buffer (Hanks Buffered Salt Solution containing 20 mM HEPES, 1 mM CaCl_2_, 1 mM MgCl_2_ and 2.5 mM probenecid, pH 7.4) supplemented with 6 mM Fluo–4/AM at 37 °C for 1 h. Then, the buffer was aspirated, the cells were washed once with 100 µL assay buffer, and then, 100 µL assay buffer was added to the cells (in the antagonist experiments, the test compound was added at this point). The 96-well plate was assayed in a FLEXStation (Molecular Devices, Crawley, UK) measuring emission (in fluorescence units (FU)) at 525 nm caused by excitation at 485 nm before and up to 90 s after the addition of 33.3 µL agonist-containing assay buffer. Antagonist activity was tested by adding the compounds on the cells in the assay buffer prior to agonist application, and Glu EC_80_ was used as agonist concentration. The compound was tested in duplicate both as agonist and antagonist at least three times at the cell line.

#### 3.2.4. mGluR4/5 Functional Assay

Dulbecco’s modified Eagle medium (DMEM), dialyzed fetal bovine serum (FBS), Opti-MEM, penicillin-streptomycin, Dulbecco’s phosphate-buffered saline (DPBS), Hank’s balanced salt solution (HBSS) and Lipofectamine 2000 were purchased from Thermo Fisher Scientific (Waltham, MA, USA). An IP-One G_q_ kit was purchased from Cisbio (Codolet, France). Quisqualate and poly-D-lysine (PDL) hydrobromide were purchased from Sigma-Aldrich (St. Louis, MO, USA). Falcon clear, tissue culture-treated 96-well plates were from Corning Inc. (Corning, NY, USA). OptiPlate-384 white, opaque 384-well plates were from PerkinElmer, (Waltham, MA, USA). L-AP4 and LY341495 were purchased from Tocris Bioscience (Bristol, UK). The HEK293A cells were a kind gift from Dr. Asuka Inoue (Tohoku University, Sendai, Japan). Plasmids encoding human mGluR4 and Flag-tagged human mGluR5 were kind gifts from Jesper M. Mathiesen (University of Copenhagen, København, Denmark). The plasmid encoding Gqo5 was a kind gift from Dr. B.R. Conklin (University of San Francisco, San Francisco, CA, USA).

Cell culture and IP_1_ Assay. HEK293A cells were cultured in DMEM with high (4.5 g/L) glucose and GlutaMAX supplemented with 10% dialyzed FBS and 100 U/mL penicillin-streptomycin at 37 °C and 5% CO_2_ in a humidified incubator. For measuring IP_1_ accumulation from mGluR4 and mGluR5, HEK293A cells were transiently transfected with Lipofectamine 2000 using a reverse transfection protocol. For the transfection of 1 mL cells, 1.6 µL of Lipofectamine 2000 was diluted in 100 µL Opti-MEM. Then, 5 min later, it was mixed with 640 ng DNA diluted in Opti-MEM. After 20–25 min the mix was added to 480,000 cells in 1 mL culture medium, and 100 µL (40,000 cells) was distributed to each well in PDL-coated Falcon 96-well cell culture plates. For mGluR4, 1 mL of cells was transfected with 320 ng mGluR4, 80 ng EAAT3, 160 ng Gqo5 and 80 ng pcDNA3.1(+). For mGluR5, 1 mL of cells was transfected with 60 ng mGluR5, 80 ng EAAT3 and 500 ng pcDNA3.1(+). 24 h after transfection; then, cells were washed once with assay buffer (HBSS supplemented with 1 mM CaCl_2_, 1 mM MgCl_2_ and 20 mM HEPES pH 7.4) and incubated for 2 h at 37 °C to remove ambient glutamate. The assay buffer was replaced with 50 µL assay buffer or antagonist and incubated for 30 min at 37 °C. Then, 20 µL agonist diluted in assay buffer containing 40 mM (final concentration) LiCl was added to each well, and the plates were incubated for 30 min at 37 °C. After stimulation, wells were emptied and 30 µL of Lysis & Detection Buffer 5 (IP-One Gq kit) was added and incubated for 30 min at room temperature. Then, 10 µL of lysate and 10 µL of detection solution were transferred to an OptiPlate-384 white, opaque 384-well plate, and the plate was incubated for 1 h at room temperature. IP_1_ accumulation was measured on an EnVision 2104 Multilabel Reader using a 340/60 nm excitation filter and 615/8.5 nm (donor) and 665/7.5 nm (acceptor) emission filters. HTRF ratios were calculated as the ratio of acceptor over donor emission (665 nm/615 nm) and converted to IP_1_ concentrations with an IP_1_ standard curve according to the instructions from the kit manufacturer.

### 3.3. Molecular Modeling

#### 3.3.1. Homology Modeling

The protein sequence for the human GluK3 receptor was retrieved in FASTA format from the UniProt database (accession number: Q13003). The crystal structures of GluK1-LBD in complex with antagonists belonging to the group of quinoxaline-2,3-dione derivatives were used as templates (PDB codes: 6FZ4, 6SBT, 4QF9), and for each template, we employed the monomer A. GluK3-LBD homology models were constructed with Modeller 9.18 [42] using the alignments obtained from the Promals3D server [43]. Based on each template, one hundred models were built with the preservation of the ligand from the crystal structure, and, additionally, one hundred models without ligand included. A high level of optimization was applied. Furthermore, additional models were built using the SwissModel [44] using an automatically generated alignment. For each template, the one best model generated by SWISS-MODEL and the three best models (according to DOPEscore) built in Modeller were submitted for further evaluation. In this way, a pool of 21 different GluK3-LBD homology models was obtained. Their quality was assessed using the DOPE score, QMEAN, and Ramachandran plot. The models with the best parameters were selected for further docking studies.

The final GluK3-LBD homology model was selected based on the usefulness of the model to distinguish active from inactive compounds as well as the ability to explain the structure–activity relationship for the compounds studied. Ligands for enrichment calculations were selected from data from the literature. Active compounds constituted 10% of the total database. The enrichment plot was based on the MM-GBSA dG bind calculated for the best docking poses of each ligand. For the MM-GBSA calculations, default options of Prime MMGBSA were used, including the VSGB solvation model, OPLS4 force field and no protein flexibility (Supplementary Materials, Figure S2).

A model built on the 6SBT template using Modeller, without a conserved ligand, was found to be the best model.

#### 3.3.2. Docking Studies

Docking studies were performed using the Glide from the Schrödinger Suite [35]. All ligands were prepared with the LigPrep module, and the ionization states were predicted under physiological conditions (pH = 7.4 ± 0.5) with the Epik program. The optimization of the ligands was performed using the OPLS4 force field. GluK1-LBD crystal structures and GluK3-LBD homology models were prepared in the Protein Preparation Wizard (addition of hydrogen atoms, removal of water molecules, sulphate ions, chloride ions, and glycerol, assignment of protonation states of residues, and optimization of the hydrogen bond network). The optimal docking settings for each of the GluK1-LBD crystal structures were established by redocking the ligands therein. The same settings were then applied for docking to the GluK3-LBD homology models built on each template. The grid center for GluK1-LBD (6SBT) was defined by a bound ligand, which was also the center of the grid for the GluK3-LBD homology model after its superimposition with the 6SBT template. In both cases, the size of the inner box was 10 Å × 10 Å × 10 Å, and the size of the outer box was 20 Å × 20 Å × 20 Å. In Glide docking, the extra precision (XP) option and OPLS4 force field were applied. The obtained ligand-protein complexes were ranked according to the calculated values of the Docking Score and Glide Score functions (Supplementary Materials, Figure S3).

For the graphical presentation of the selected docking poses with the highest docking scores, we used PyMOL software [45]. Interactions marked in the figures have been assigned by the Schrödinger software, and the criteria are defined as follows:

hydrogen bond: max. distance 2.8 Å, donor min. angle 120°, acceptor min. angle 90°;

halogen bond: max. distance 3.5 Å;

salt bridges: max. distance 5.0 Å.

π–π interactions “face to face”: max. distance between ring centroids 4.4 Å, max. angle 30°;

π–π interactions “edge to face”: max. distance between ring centroids 5.5 Å, min. angle 60°;

π–cation interactions: max. distance 6.6 Å, max. angle 30°.

Due to the options implemented in the used software, NH–π and CH–π interactions are labeled in the figures as “edge to face” π–π interactions between ring centroids, and they are marked as pink dashes.

#### 3.3.3. Molecular Dynamics

Molecular dynamics simulations were performed for dimers of the GluK1–LBD and GluK3–LBD complexes with selected ligands. For GluK1, we used dimers from the 6SBT crystal structure. For GluK3, the dimers were obtained by superimposing two monomers on the GluK1 dimer. The side chains of the amino acids constituting the interface between the individual GluK3 monomers were optimized with Prime. MD simulations were performed in NAMD 2.13 using the CHARMM36m force field [46]. The input files were prepared with the CHARMM-GUI online server. The dimers of the protein-ligand complexes were solvated with TIP3P water molecules. The size of the water box was 102 Å × 102 Å × 102 Å in the case of GluK1 and 109 Å × 109 Å × 109 Å in the case of GluK3. Sodium and chloride ions (0.15 M NaCl) were added to provide standard physiological ionic strength. The system was equilibrated via the one-step protocol suggested by CHARM-GUI. The MD simulations were run at 303.15 K with a time step of 2 fs and a total duration of 20 ns. The intervals for both the energy and the trajectory recordings were 10 ps. The results were analyzed with the VMD program [47] (Supplementary Materials, Figures S4 and S5). Selected snapshots were illustrated with the PyMOL software [45].

## 4. Conclusions

Taking the previously reported *N*-(2,3-dioxo-6-(phenylethynyl)-3,4-dihydroquinoxalin-1(2*H*)-yl)benzamide **1** as a lead structure, we have synthesized 25 new derivatives of quinoxaline-2,3-dione with various arylethynyl substituents at position 6. Compounds were evaluated in the preliminary radioligand binding studies for their affinity for recombinant rat homomeric iGluR subtypes: GluK1-GluK3, GluK5 and GluA2 receptors. This assay allowed the selection of the most potent compounds **27–29**, for which antagonist properties toward individual receptor subtypes were confirmed in the intracellular Ca^2+^ imaging assay. Compound **29** demonstrated the highest binding affinity across the GluK1−3 and GluA2 receptors within the obtained series, while **28** showed the highest GluK3-selectivity, with a preference for GluK3 receptors of at least 400 times over the GluK1, GluK2, GluK5 and GluA2 receptors. Furthermore, **28** displayed no agonist nor antagonist activity at any of tested metabotropic glutamate receptor subtypes, which are representatives of the three mGluR families. The molecular factors underlying the observed in vitro results for **28** and **29** were examined by molecular docking and molecular dynamics methods, using the X-ray structure of GluK1-LBD (6SBT) as well as a homology model of GluK3-LBD.

To our knowledge, compound **28** is the first competitive GluK3 antagonist to show such a high selectivity profile among all other GluK3 antagonists. Taking into account the high potential of **28** in a further search for GluK3-selective pharmacological tools, we are going to evaluate this compound or its close analogues in more advanced in vitro and ex vivo pharmacological assays.

## Figures and Tables

**Table 1:** Receptor binding affinities (mean ± SEM) at recombinant rat iGluRs.

cmpd	R	*K*_i_ (µM)				
		GluK1	GluK2	GluK3	GluK5	GluA2
**DNQX ^1^**		0.65 ± 0.03	2.1 ± 0.3	0.36 ± 0.03	7.1 ± 0.9	0.25 ± 0.01
**NBQX**		2.6 ± 0.1	5.4 ± 1.2	3.4 ± 0.6	152 ± 23	0.077 ± 0.010
**1 ^2^**	Ph	>100	≈100	2.9 ± 0.3	>100	24 ± 6
**5**	3-Me-Ph	≈100	≈100	1.4 ± 0.4	>100	>100
**6**	4-Me-Ph	≥100	nd	6.1 ± 0.8	nd	nd
**7**	4-Et-Ph	>100	>100	20 ± 1	>100	>100
**8**	4-*n*Pr-Ph	>100	>100	50 ± 12	>100	>100
**9**	4-*i*Pr-Ph	>100	>100	38 ± 10	>100	>100
**10**	4-Cl-Ph	>100	>100	3.5 ± 0.7	>100	>100
**11**	4-CF_3_-Ph	≈100	>100	>100	>100	>100
**12**	4-MeO-Ph	>100	>100	8.3 ± 0.4	>100	>100
**13**	4-PhO-Ph	≥100	nd	7.9 ± 0.7	nd	nd
**14**	3-COOH-Ph	>100	>100	28 ± 2	>100	>100
**15**	4-COOH-Ph	>100	>100	31 ± 4	>100	>100
**16**	4-NH_2_SO_2_-Ph	>100	>100	≈100	>100	>100
**17**	4-FSO_2_-Ph	1.1 ± 0.4	>100	8.1 ± 0.4	>100	≈100
**18**	2-OH-Ph	>100	nd	14 ± 2	nd	nd
**19**	3-OH-Ph	>100	>100	3.2 ± 0.6	>100	≈100
**20**	4-OHCH_2_-Ph	≈100	>100	5.8 ± 1.5	>100	>100
**21**	2-NH_2_-Ph	14 ± 2	nd	1.0 ± 0.1	nd	nd
**22**	3-NH_2_-Ph	>100	nd	≈100	nd	nd
**23**	4-NH_2_-Ph	>100	>100	1.4 ± 0.2	>100	>100
**24**	4-N(CH_3_)_2_-Ph	39 ± 4	nd	24 ± 4	nd	nd
**25**	pyridin-4-yl	>100	>100	25 ± 6	>100	>100
**26**	6-hydroxypyridin-3-yl	>100	>100	11 ± 1	>100	>100
**27**	pyrimidin-5-yl	11 ± 2	12 ± 2	0.28 ± 0.02	>100	>100
**28**	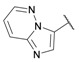	≈100	>100	0.25 ± 0.01	>100	>100
**29**	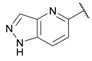	0.15 ± 0.05	0.091 ± 0.015	0.13 ± 0.03	3.8 ± 0.1	0.23 ± 0.02

^1^ Data from the reference [34]; ^2^ Data from the reference [33]; nd—not determined.

## Data Availability

Not applicable.

## Supplementary Materials

The following supporting information can be downloaded at: https://www.mdpi.com/article/10.3390/ijms23158797/s1.
