# Supplementary material for: Discovery of the First Highly Selective Antagonist of the GluK3 Kainate Receptor Subtype
Source: Int J Mol Sci. 2022 Aug 8;23(15):8797. doi: 10.3390/ijms23158797 (PMC9369419; doi:10.3390/ijms23158797)

Supplementary Materials

Discovery of the first highly selective antagonist of the GluK3 kainate receptor subtype

Paulina Chałupnik [1], Alina Vialko [1], Darryl S. Pickering [2], Markus Hinkkanen [2], Stephanie Donbosco [2], Thor C. Møller [2], Anders A. Jensen [2], Birgitte Nielsen [2], Yasmin Bay [2], Anders S. Kristensen [2], Tommy N. Johansen [2], Kamil Łątka [3], Marek Bajda [3] and Ewa Szymańska [1,*]

[1] *Department of Technology and Biotechnology of Drugs, Jagiellonian University Medical College in Kraków, PL 30-688 Kraków, Poland*
[2] *Department of Drug Design and Pharmacology, Faculty of Health and Medical Sciences, University of Copenhagen, DK-2100 Copenhagen, Denmark*
[3] *Department of Physicochemical Drug Analysis, Jagiellonian University Medical College in Kraków, PL 30-688 Kraków, Poland*
***** *Correspondence: ewa.szymanska@uj.edu.pl*

Table of Contents

ANALYTICAL CHARACTERIZATION OF THE FINAL COMPOUNDS 5-29 2

SUPPLEMENTARY FIGURES 10

NMR SPECTRA 13

Analytical characterization of the final compounds **5-29**

N-(2,3-dioxo-6-(m-tolylethynyl)-3,4-dihydroquinoxalin-1(2H)-yl)benzamide **(5)**

Yield: 58%, mp. 318-320°C Anal. for $C_{24}H_{17}N_3O_3$: Calc.: C, 72.90; H, 4.33; N, 10.63. Found: C, 72.69; H, 4.43; N, 10.39. **LC/MS**: purity 100%, t_{ret}= 6.84 min, *m/z* 396.29 [M^++1]. **^{1}H NMR** (500 MHz, DMSO-d_6) δ 2.29 (s, 3H, CH_3), 7.21 (d, *J* = 7.6 Hz, 1H, Ar), 7.28 (t, *J* = 7.6 Hz, 1H, Ar), 7.31 – 7.39 (m, 5H, Ar), 7.57 (t, *J* = 8.4 Hz, 2H, Ar), 7.66 (tt, *J* = 7.6, 1.3 Hz, 1H, Ar), 8.01 (dd, *J* = 7.0, 1.4 Hz, 2H, Ar), 11.73 (s, 1H, NH), 12.43 (s, 1H, NH). **^{13}C NMR** (126 MHz, DMSO-d_6) δ 21.2 (CH_3), 88.6 (C≡C), 90.2 (C≡C), 115.0, 118.6, 118.7, 122.4, 125.2, 127.4, 128.5, 128.7, 129.0, 129.2, 129.3, 130.3, 131.7, 132.4, 133.4, 138.7, 154.0 (C=O), 154.6 (C=O), 165.9 (C=O).

N-(2,3-dioxo-6-(p-tolylethynyl)-3,4-dihydroquinoxalin-1(2H)-yl)benzamide **(6)**

Yield: 61%, mp. 325-327°C Anal. for $C_{24}H_{17}N_3O_3$: Calc.: C, 72.90; H, 4.33; N, 10.63. Found: C, 73.01; H, 4.28; N, 10.74. **LC/MS**: purity 100%, t_{ret}= 6.86 min, *m/z* 396.25 [M^++1]. **^{1}H NMR** (500 MHz, DMSO-d_6) δ 2.30 (s, 3H, CH_3), 7.20 (d, *J* = 8.0 Hz, 2H, Ar), 7.30 – 7.32 (m, 2H, Ar), 7.34 (s, 1H, Ar), 7.43 (d, *J* = 7.8 Hz, 2H, Ar), 7.57 (t, *J* = 7.8 Hz, 2H, Ar), 7.66 (tt, *J* = 7.7, 1.4 Hz, 1H, Ar), 8.01 (d, *J* = 7.2 Hz, 2H, Ar), 11.73 (br. s, 1H, NH), 12.42 (br. s, 1H). **^{13}C NMR** (126 MHz, DMSO-d_6) δ 21.6 (CH_3), 88.3 (C≡C), 90.2 (C≡C), 115.0, 118.5, 118.8, 119.5, 125.2, 127.3, 128.5, 128.6, 129.3, 130.0, 131.6, 131.9, 133.4, 139.3, 154.0 (C=O), 154.6 (C=O), 165.9 (C=O).

N-(6-((4-ethylphenyl)ethynyl)-2,3-dioxo-3,4-dihydroquinoxalin-1(2H)-yl)benzamide **(7)**

Yield: 78%, mp. 308-310°C Anal. for $C_{25}H_{19}N_3O_3$ x 0.5 H_2O: Calc.: C, 71.76; H, 4.82; N, 10.04. Found: C, 71.88; H, 4.76; N, 9.80. **LC/MS**: purity 100%, t_{ret}= 7.28 min, *m/z* 410.32 [M^++1]. **^{1}H NMR** (500 MHz, DMSO-d_6) δ 1.15 (t, *J* = 7.6 Hz, 3H, $CH_2\underline{CH_3}$), 2.60 (q, *J* = 7.6 Hz, 2H, $\underline{CH_2}CH_3$), 7.24 (d, *J* = 8.2 Hz, 2H, Ar), 7.31 – 7.32 (m, 2H), 7.34 (s, 1H), 7.46 (d, *J* = 8.2 Hz, 2H, Ar), 7.57 (t, *J* = 7.9 Hz, 2H, Ar), 7.66 (tt, *J* = 7.4, 1.9 Hz, 1H, Ar), 8.01 (dd, *J* = 7.2, 1.4 Hz, 2H, Ar), 11.83 (br. s, 1H, NH), 12.32 (br. s, 1H, NH). **^{13}C NMR** (126 MHz, DMSO-d_6) δ 15.8 ($CH_2\underline{CH_3}$), 28.6 ($\underline{CH_2}CH_3$), 88.3 (C≡C), 90.2 (C≡C), 115.0, 118.6, 118.8, 119.8, 125.2, 127.3, 128.5, 128.7, 128.8, 129.3, 131.7, 132.0, 133.4, 145.5, 154.0 (C=O), 154.6 (C=O), 165.8 (C=O).

N-(2,3-dioxo-6-((4-propylphenyl)ethynyl)-3,4-dihydroquinoxalin-1(2H)-yl)benzamide **(8)**
Yield: 63%, mp. 305-306°C Anal. for $C_{26}H_{19}N_3O_3$ x 0.5 H_2O: Calc.: C, 72.21; H, 5.13; N, 9.72. Found: C, 72.45; H, 5.10; N, 9.66. **LC/MS**: purity 100%, t_{ret}= 7.77 min, *m/z* 424.21 [M^++1]. **^{1}H NMR** (500 MHz, DMSO-d_6) δ 0.85 (t, *J* = 7.3 Hz, 3H, $CH_2CH_2\underline{CH_3}$), 1.50 – 1.61 (m, 2H, $CH_2\underline{CH_2}CH_3$), 2.55 (t, *J* = 7.6 Hz, 2H, $\underline{CH_2}CH_2CH_3$), 7.21 (d, *J* = 7.8 Hz, 2H, Ar), 7.29 – 7.35 (m, 3H), 7.45 (d, *J* = 7.8 Hz, 2H, Ar), 7.57 (t, *J* = 7.6 Hz, 2H, Ar), 7.66 (t, *J* = 7.4 Hz, 1H, Ar), 8.01 (d, *J* = 7.6 Hz, 2H, Ar), 11.72 (br. s, 1H, NH), 12.42 (br. s, 1H, NH). **^{13}C NMR** (126 MHz, DMSO-d_6) δ 14.1 ($CH_2CH_2\underline{CH_3}$), 24.3 ($CH_2\underline{CH_2}CH_3$), 37.6 ($\underline{CH_2}CH_2CH_3$), 88.3 (C≡C), 90.2 (C≡C), 115.0, 118.6, 118.8, 119.8, 125.2, 127.3, 128.5, 128.7, 129.3, 129.3, 131.7, 131.9, 133.4, 143.9, 154.0 (C=O), 154.6 (C=O), 165.8 (C=O).

N-(6-((4-isopropylphenyl)ethynyl)-2,3-dioxo-3,4-dihydroquinoxalin-1(2H)-yl)benzamide **(9)**
Yield: 68%, mp. 263-264°C Anal. for $C_{26}H_{21}N_3O_3$ x 1 H_2O: Calc.: C, 70.74; H, 5.25; N, 9.52. Found: C, 70.43; H, 4.97; N, 9.44. **LC/MS**: purity 96.99%, t_{ret}= 7.66 min, *m/z* 424.06 [M^++1]. **^{1}H NMR** (500 MHz, DMSO-d_6) δ 1.16 (d, *J* = 6.9 Hz, 6H, CH_3), 2.82 – 2.93 (m, 1H, $\underline{CH}(CH_3)_2$), 7.26 (d, *J* = 7.9 Hz, 2H, Ar), 7.28 – 7.36 (m, 3H, Ar), 7.46 (d, *J* = 7.8 Hz, 2H, Ar), 7.57 (t, *J* = 7.5 Hz, 2H, Ar), 7.66 (t, *J* = 7.5 Hz, 1H, Ar), 8.02 (d, *J* = 7.6 Hz, 2H, Ar), 11.74 (br. s, 1H, NH), 12.44 (br. s, 1H, NH). **^{13}C NMR** (126 MHz, DMSO-d_6) δ 24.1 (CH_3), 33.9 ($\underline{CH}(CH_3)_2$), 88.2 (C≡C), 90.2 (C≡C), 115.0, 118.6, 118.8, 119.9, 125.2, 127.3, 128.5, 128.7, 129.3, 131.7, 132.0, 133.4, 150.0, 154.0 (C=O), 154.6 (C=O), 165.8 (C=O).

N-(6-((4-chlorophenyl)ethynyl)-2,3-dioxo-3,4-dihydroquinoxalin-1(2H)-yl)benzamide **(10)**
Yield: 76%, mp. 326-327°C Anal. for $C_{23}H_{14}ClN_3O_3$: Calc.: C, 66.43; H, 3.39; N, 10.11. Found: C, 66.15; H, 3.50; N, 9.96. **LC/MS**: purity 100%, t_{ret}= 7.01 min, *m/z* 416.36 [M^+]. **^{1}H NMR** (500 MHz, DMSO-d_6) δ 7.33 – 7.34 (m, 2H, Ar), 7.36 (t, *J* = 1.2 Hz, 1H, Ar), 7.47 (dt, *J* = 8.6, 2.1 Hz, 2H, Ar), 7.53 – 7.60 (m, 4H, Ar), 7.66 (tt, *J* = 7.4, 1.8 Hz, 1H, Ar), 8.01 (dd, *J* = 7.5, 1.4 Hz, 2H), 11.73 (s, 1H, NH), 12.44 (s, 1H, NH). **^{13}C NMR** (126 MHz, DMSO-d_6) δ 88.9 (C≡C), 89.9 (C≡C), 115.0, 118.3, 118.7, 121.4, 125.3, 127.5, 128.5, 128.9, 129.3, 129.5, 131.6, 133.4, 133.7, 134.2, 154.0 (C=O), 154.5 (C=O), 165.9 (C=O).

N-(2,3-dioxo-6-((4-(trifluoromethyl)phenyl)ethynyl)-3,4-dihydroquinoxalin-1(2H)-yl)benzamide **(11)**

Yield: 63%, mp. 317-319°C Anal. for $C_{24}H_{14}F_3N_3O_3$ x 1 H_2O: Calc.: C, 61.67; H, 3.19; N, 8.99. Found: C, 62.02; H, 3.19; N, 8.97. **LC/MS**: purity 100%, t_{ret}= 7.15 min, *m/z* 450.19 [M^++1]. **^{1}H NMR** (500 MHz, DMSO-*d*$_6$) δ 7.33 – 7.44 (m, 3H, Ar), 7.57 (t, *J* = 7.7 Hz, 2H, Ar), 7.66 (t, *J* = 7.2 Hz, 1H, Ar), 7.73 – 7.80 (m, 4H, Ar), 8.01 (d, *J* = 7.2 Hz, 2H, Ar), 11.76 (s, 1H, NH), 12.46 (s, 1H, NH). **^{13}C NMR** (126 MHz, DMSO-*d*$_6$) δ 88.5 (C≡C), 91.4 (C≡C), 115.1, 117.8, 118.9, 124.5, 125.3, 126.2, 126.9, 127.6, 128.6, 129.2, 129.3, 131.7, 132.7, 133.4, 154.0 (C=O), 154.5 (C=O), 165.8 (C=O).

N-(6-((4-methoxyphenyl)ethynyl)-2,3-dioxo-3,4-dihydroquinoxalin-1(2H)-yl)benzamide **(12)**
Yield: 76%, mp. 304-305°C Anal. for $C_{24}H_{17}N_3O_4$ x 0.5 H_2O: Calc.: C, 68.57; H, 4.32; N, 9.99. Found: C, 68.80; H, 4.27; N, 9.87. **LC/MS**: purity 100%, t_{ret}= 6.30 min, *m/z* 412.18 [M^++1]. **^{1}H NMR** (500 MHz, DMSO-*d*$_6$) δ 3.76 (s, 3H, CH_3), 6.95 (dt, *J* = 8.8, 1.9 Hz, 2H, Ar), 7.29 – 7.31 (m, 2H, Ar), 7.31 – 7.33 (m, 1H), 7.48 (t, *J* = 8.8, 2.7 Hz, 2H, Ar), 7.56 (t, *J* = 7.3 Hz, 2H), 7.66 (tt, *J* = 7.5, 1.3 Hz, 1H), 8.01 (dd, *J* = 7.2, 1.3 Hz, 2H), 11.72 (br. s, 1H, NH), 12.41 (br. s, 1H, NH). **^{13}C NMR** (126 MHz, DMSO-*d*$_6$) δ 55.8 (CH_3), 87.5 (C≡C), 90.2 (C≡C), 114.4, 114.9, 115.0, 118.4, 119.1, 125.2, 127.2, 128.5, 128.5, 129.3, 131.7, 133.4, 133.6, 154.0 (C=O), 154.6 (C=O), 160.22 ($\underline{C_{Ar}}$-OCH_3), 165.8 (C=O).

N-(2,3-dioxo-6-((4-phenoxyphenyl)ethynyl)-3,4-dihydroquinoxalin-1(2H)-yl)benzamide **(13)**
Yield: 75%, mp. 262-263°C, Anal. for $C_{29}H_{19}N_3O_4$ x 1 H_2O: Calc.: C, 70.87; H, 4.31; N, 8.55. Found: C, 70.89; H, 4.18; N, 8.41. **LC/MS**: purity 100%, t_{ret}= 7.66 min, *m/z* 474.14 [M^++1]. **^{1}H NMR** (500 MHz, DMSO-*d*$_6$) δ 6.97 (dt, *J* = 8.7, 2.0 Hz, 2H, Ar), 7.06 (dd, *J* = 7.6, 0.9 Hz, 2H, Ar), 7.18 (tt, *J* = 7.2, 1.1 Hz, 1H, Ar), 7.27 – 7.36 (m, 3H, Ar), 7.36 – 7.44 (m, 2H, Ar), 7.52 – 7.60 (m, 4H, Ar), 7.66 (tt, *J* = 7.3, 1.5 Hz, 1H, Ar), 8.01 (d, *J* = 7.2 Hz, 2H, Ar), 11.73 (br. s, 1H, NH), 12.43 (br. s, 1H, NH). **^{13}C NMR** (126 MHz, DMSO-*d*$_6$) δ 88.2 (C≡C), 89.7 (C≡C), 115.0, 117.0, 118.5, 118.7, 120.1, 124.9, 125.2, 127.3, 128.5, 128.7, 129.3 130.8, 131.7, 133.4, 134.0, 154.0 (C=O), 154.5 (C=O), 156.1, 158.1, 165.8 (C=O).

3-((1-benzamido-2,3-dioxo-1,2,3,4-tetrahydroquinoxalin-6-yl)ethynyl)benzoic acid **(14)**
Yield: 55%, mp. 310-312°C Anal. for $C_{24}H_{15}N_3O_5$: Calc.: C, 67.76; H, 3.55; N, 9.88. Found: C, 67.90; H, 3.71; N, 9.91. **LC/MS**: purity 96.28%, t_{ret}= 5.23 min, *m/z* 426.35 [M^++1]. **^{1}H NMR** (300 MHz, DMSO-*d*$_6$) δ 7.36 – 7.39 (m, 2H, Ar), 7.41 (d, *J* = 1.6 Hz, 1H, Ar), 7.49 – 7.64 (m, 3H, Ar), 7.68 (tt, *J* = 7.4, 2.4, 1.3 Hz, 1H, Ar), 7.80 (dt, *J* = 8.1, 1.2 Hz, 1H, Ar), 7.95 (dt, *J* = 7.9, 1.6 Hz, 1H, Ar), 8.01 – 8.07 (m, 3H, Ar), 11.74 (br.s, 1H, NH), 12.46 (br. s, 1H, NH).

^{13}C NMR (126 MHz, DMSO-d_6) δ 89.0 (C≡C), 89.6 (C≡C), 115.0, 118.2, 118.7, 123.0, 125.3, 127.6, 128.5, 129.0, 129.3, 129.8, 130.1, 131.7, 132.0, 132.6, 133.4, 135.9, 154.0 (C=O), 154.5 (C=O), 165.9 (C=O), 167.1 (COOH).

4-((1-benzamido-2,3-dioxo-1,2,3,4-tetrahydroquinoxalin-6-yl)ethynyl)benzoic acid **(15)**
Yield: 64%, mp. 348-349°C Anal. for $C_{24}H_{15}N_3O_5$ x 1.5 H_2O: Calc.: C, 63.72; H, 4.01; N, 9.29. Found: C, 64.04; H, 4.21; N, 8.97. **LC/MS**: purity 97.78%, t_{ret}= 5.04 min, *m/z* 424.09 [M^+-1]. **^{1}H NMR** (500 MHz, DMSO-d_6) δ 7.32 – 7.40 (m, 3H, Ar), 7.57 (t, *J* = 7.7 Hz, 2H, Ar), 7.63 – 7.70 (m, 3H, Ar), 7.93 (d, *J* = 9.0 Hz, 2H, Ar), 8.01 (d, *J* = 7.3 Hz, 2H, Ar), 11.74 (s, 1H, NH), 12.46 (s, 1H, NH), 13.15 (br. s, 1H, COOH). **^{13}C NMR** (126 MHz, DMSO-d_6) δ 89.3 (C≡C), 91.5 (C≡C), 115.1, 118.0, 118.8, 125.3, 126.8, 127.6, 128.5, 129.1, 129.3, 130.1, 131.3, 131.6, 132.1, 133.4, 154.0 (C=O), 154.5 (C=O), 165.9 (C=O), 167.3 (COOH).

N-(2,3-dioxo-6-((4-sulfamoylphenyl)ethynyl)-3,4-dihydroquinoxalin-1(2H)-yl)benzamide **(16)**
Yield: 59%, mp. 303-305°C Anal. for $C_{23}H_{16}N_4O_5S$: Calc.: C, 59.99; H, 3.50; N, 12.17. Found: C, 60.11; H, 3.57; N, 12.38. **LC/MS**: purity 98.07%, t_{ret}= 4.72 min, *m/z* 461.09 [M^++1]. **^{1}H NMR** (500 MHz, DMSO-d_6) δ 7.36 (s, 2H, Ar), 7.40 (s, 1H, Ar), 7.46 (s, 2H, SO_2NH_2), 7.56 (t, *J* = 7.5 Hz, 2H, Ar), 7.65 (t, *J* = 7.4 Hz, 1H, Ar), 7.74 (d, *J* = 8.0 Hz, 2H, Ar), 7.83 (d, *J* = 8.0 Hz, 2H, Ar), 8.02 (d, *J* = 7.6 Hz, 2H, Ar). **^{13}C NMR** (126 MHz, DMSO-d_6) δ 88.8 (C≡C), 91.3 (C≡C), 115.1, 117.8, 118.9, 125.4, 125.9, 126.6, 127.6, 128.5, 129.3, 131.8, 132.5, 133.3, 144.4, 154.0 (C=O), 154.6 (C=O), 165.9 (C=O).

4-((1-benzamido-2,3-dioxo-1,2,3,4-tetrahydroquinoxalin-6-yl)ethynyl)benzenesulfonyl fluoride **(17)**
Yield: 69%, mp. 319-320°C Anal. for $C_{23}H_{14}FN_3O_5S$ x 2 H_2O: Calc.: C, 55.31; H, 3.63; N, 8.41. Found: C, 55.46; H, 3.64; N, 8.44. **LC/MS**: purity 96.03%, t_{ret}= 6.59 min, *m/z* 464.15 [M^+]. **^{1}H NMR** (500 MHz, DMSO-d_6) δ 7.34 (d, *J* = 1.1 Hz, 2H, Ar), 7.36 (t, *J* = 1.2 Hz, 1H, Ar), 7.47 (dt, *J* = 8.5, 2.1 Hz, 2H, Ar), 7.53 – 7.60 (m, 4H, Ar), 7.66 (tt, *J* = 7.4, 1.3 Hz, 1H, Ar), 8.01 (dd, *J* = 6.9, 1.6 Hz, 2H, Ar), 11.73 (s, 1H, NH), 12.44 (s, 1H, NH). **^{13}C NMR** (126 MHz, DMSO-D_6) δ 88.0 (C≡C), 94.2 (C≡C), 115.2, 117.3, 119.1, 125.4, 127.9, 128.5, 129.31, 129.34, 129.6, 130.8, 131.3 (d, *J* = 23.9 Hz, C_{Ar}-SO_2F, 131.6, 133.4, 133.5, 154.0 (C=O), 154.5 (C=O), 165.9 (C=O).

N-(6-((2-hydroxyphenyl)ethynyl)-2,3-dioxo-3,4-dihydroquinoxalin-1(2H)-yl)benzamide **(18)**

Yield: 58%, mp. 349-351°C Anal. for $C_{23}H_{15}N_3O_4$ x 0.5 H_2O: Calc.: C, 67.98; H, 3.97; N, 10.34. Found: C, 68.15; H, 3.93; N, 10.11. **LC/MS**: purity 100%, t_{ret}= 6.26 min, *m/z* 398.18 [M^++1]. **^{1}H NMR** (500 MHz, DMSO-d_6) δ 7.24 (t, *J* = 7.4 Hz, 1H, Ar), 7.30 (t, *J* = 7.7 Hz, 1H, Ar), 7.36 (s, 1H, OH), 7.41 (d, *J* = 8.6 Hz, 1H, Ar), 7.55 – 7.76 (m, 7H, Ar), 8.03 (d, *J* = 7.6 Hz, 2H, Ar), 11.75 (s, 1H, NH), 12.46 (s, 1H, NH). **^{13}C NMR** (126 MHz, DMSO-D_6) δ 102.8, 111.7, 112.0, 115.3, 121.0, 121.8, 123.9, 125.3, 125.5, 126.6, 128.5, 128.7, 129.3, 131.7, 133.4, 154.0 (C=O), 154.60 (C=O), 154.6, 154.8, 165.9 (C=O).

N-(6-((3-hydroxyphenyl)ethynyl)-2,3-dioxo-3,4-dihydroquinoxalin-1(2H)-yl)benzamide **(19)**
Yield: 71%, mp. 313-314°C Anal. for $C_{23}H_{15}N_3O_4$ x 1 H_2O: Calc.: C, 66.5; H, 4.12; N, 10.12. Found: C, 66.67; H, 4.20; N, 9.87. **LC/MS**: purity 100%, t_{ret}= 5.34 min, *m/z* 398.18 [M^++1]. **^{1}H NMR** (300 MHz, DMSO-d_6) δ 6.81 (ddd, *J* = 8.2, 2.5, 1.0 Hz, 1H, Ar), 6.86 – 6.93 (m, 1H, Ar), 6.97 (dt, *J* = 8.0, 1.0 Hz, 1H, Ar), 7.21 (t, *J* = 7.9 Hz, 1H, Ar), 7.33 (d, *J* = 1.0 Hz, 2H, Ar), 7.35 – 7.37 (m, 1H, Ar), 7.59 (tt, *J* = 7.2, 1.2 Hz, 2H, Ar), 7.68 (tt, *J* = 7.3, 1.5 Hz, 1H, Ar), 8.03 (dt, *J* = 6.9, 2.0 Hz, 2H, Ar), 9.69 (s, 1H, OH), 11.73 (br. s, 1H, NH), 12.39 (br. s, 1H, NH). **^{13}C NMR** (126 MHz, DMSO-d_6) δ 88.3 (C≡C), 90.2 (C≡C), 115.0, 117.0, 118.3, 118.6, 118.7, 122.8, 123.4, 125.2, 127.4, 128.5, 128.7, 129.3, 130.5, 131.7, 133.4, 154.0 (C=O), 154.54 (C=O), 157.9 (C_{Ar}-OH), 165.9 (C=O).

N-(6-((4-(hydroxymethyl)phenyl)ethynyl)-2,3-dioxo-3,4-dihydroquinoxalin-1(2H)-yl)benzamide **(20)**
Yield: 75%, mp. 298-300°C Anal. for $C_{24}H_{17}N_3O_4$ x 2 H_2O: Calc.: C, 64.42; H, 4.73; N, 9.39. Found: C, 64.71; H, 4.58; N, 9.33. **LC/MS**: purity 98.77%, t_{ret}= 4.98 min, *m/z* 412.26 [M^++1]. **^{1}H NMR** (300 MHz, DMSO-d_6) δ 4.52 (d, *J* = 5.3 Hz, 2H, C$\underline{H_2}$OH), 5.27 (t, *J* = 5.7 Hz, 1H, OH), 7.32 – 7.39 (m, 5H, Ar), 7.52 (dt, *J* = 8.4, 1.9 Hz, 2H, Ar), 7.59 (tt, *J* = 7.0, 1.5 Hz, 2H, Ar), 7.68 (tt, *J* = 7.4, 1.4 Hz, 1H, Ar), 8.03 (dt, *J* = 7.1, 2.1 Hz, 2H, Ar), 11.73 (s, 1H, NH), 12.41 (s, 1H, NH). **^{13}C NMR** (126 MHz, DMSO-d_6) δ 63.0 ($\underline{C}H_2$OH), 88.4 (C≡C), 90.2 (C≡C), 115.0, 118.6, 118.7, 120.7, 125.2, 127.2, 127.4, 128.5, 128.7, 129.3, 131.6, 131.8, 133.4, 144.2, 154.0 (C=O), 154.6 (C=O), 165.9 (C=O).

N-(6-((2-aminophenyl)ethynyl)-2,3-dioxo-3,4-dihydroquinoxalin-1(2H)-yl)benzamide **(21)**
Yield: 71%, mp. 303-305°C Anal. for $C_{23}H_{16}N_4O_3$ x 1.5 H_2O: Calc.: C, 65.24; H, 4.52; N, 13.23. Found: C, 65.44; H, 4.61; N, 12.98. **LC/MS**: purity 100%, t_{ret}= 5.72 min, *m/z* 397.24 [M^++1]. **^{1}H NMR** (500 MHz, DMSO-d_6) δ 5.49 (s, 2H, NH_2), 6.50 (t, *J* = 7.5 Hz, 1H, Ar), 6.69

(d, J = 8.0 Hz, 1H, Ar), 7.05 (td, J = 8.6, 7.3, 1.7 Hz, 1H, Ar), 7.21 (dd, J = 7.8, 1.6 Hz, 1H, Ar), 7.31 (d, J = 8.5 Hz, 1H, Ar), 7.35 – 7.43 (m, 2H, Ar), 7.57 (t, J = 7.7 Hz, 2H, Ar), 7.66 (t, J = 7.3 Hz, 1H, Ar), 8.01 (d, J = 7.2 Hz, 2H, Ar), 11.71 (br. s, 1H, NH), 12.41 (br. s, 1H, NH). **^{13}C NMR** (126 MHz, DMSO-d_6) δ 87.8 (C≡C), 93.4 (C≡C), 105.7, 114.6, 114.8, 116.4, 118.5, 119.5, 125.1, 127.4, 128.3, 128.5, 129.3, 130.5, 131.7, 132.4, 133.4, 150.3, 154.0 (C=O), 154.6 (C=O), 165.9 (C=O).

N-(6-((3-aminophenyl)ethynyl)-2,3-dioxo-3,4-dihydroquinoxalin-1(2H)-yl)benzamide **(22)**
Yield: 64%, mp. 274-275°C Anal. for $C_{23}H_{16}N_4O_3$ x 0.5 H_2O: Calc.: C, 72.21; H, 5.13; N, 9.72. Found: C, 72.21; H, 5.10; N, 9.66. **LC/MS**: purity 100%, t_{ret}= 4.76 min, *m/z* 397.24 [M^++1]. **^{1}H NMR** (500 MHz, DMSO-d_6) δ 5.20 (br. s, 2H, NH_2), 6.57 (d, J = 7.9 Hz, 1H, Ar), 6.64 (d, J = 7.5 Hz, 1H, Ar), 6.70 (s, 1H, Ar), 7.02 (t, J = 7.8 Hz, 1H, Ar), 7.22 – 7.35 (m, 3H, Ar), 7.57 (t, J = 7.6 Hz, 2H, Ar), 7.66 (t, J = 7.4 Hz, 1H, Ar), 8.01 (d, J = 7.2 Hz, 2H, Ar), 11.73 (s, 1H, NH), 12.42 (s, 1H, NH). **^{13}C NMR** (126 MHz, DMSO-d_6) δ 87.5 (C≡C), 91.0 (C≡C), 114.9, 115.4, 116.8, 118.6, 118.9, 119.4, 122.6, 125.2, 127.3, 128.5, 128.6, 129.3, 129.8, 131.6, 133.4, 154.0 (C=O), 154.6 (C=O), 165.8 (C=O).

N-(6-((4-aminophenyl)ethynyl)-2,3-dioxo-3,4-dihydroquinoxalin-1(2H)-yl)benzamide **(23)**
Yield: 65%, mp. 312-314°C Anal. for $C_{23}H_{16}N_4O_3$ x 1 H_2O: Calc.: C, 66.66; H, 4.38; N, 13.52. Found: C, 66.54; H, 4.49; N, 13.31. **LC/MS**: purity 100%, t_{ret}= 4.93 min, *m/z* 396.85 [M^++1]. **^{1}H NMR** (300 MHz, DMSO-d_6) δ 5.57 (s, 2H, NH_2), 6.54 (dt, J = 8.6, 2.0 Hz, 2H, Ar), 7.19 (dt, J = 8.6, 1.9 Hz, 2H, Ar), 7.22 – 7.32 (m, 3H, Ar), 7.59 (tt, J = 8.3, 1.3 Hz, 2H, Ar), 7.68 (tt, J = 7.4, 1.3 Hz, 1H, Ar), 7.98 – 8.08 (m, 2H, Ar), 11.70 (s, 1H, NH), 12.38 (s, 1H, NH). **^{13}C NMR** (126 MHz, DMSO-D_6) δ 86.1 (C≡C), 92.1 (C≡C), 108.2, 114.2, 114.9, 118.0, 119.9, 125.2, 126.9, 128.0, 128.5, 129.3, 131.7, 133.2, 133.4, 150.2, 154.0 (C=O), 154.6 (C=O), 165.8 (C=O).

N-(6-((4-(dimethylamino)phenyl)ethynyl)-2,3-dioxo-3,4-dihydroquinoxalin-1(2H)-yl)benzamide **(24)**
Yield: 71%, mp. 273-275°C Anal. for $C_{19}H_{16}N_4O_3$ x 0.5 H_2O: Calc.: C, 69.27; H, 4.88; N, 12.93. Found: C, 69.54; H, 4.67; N, 13.04. **LC/MS**: purity 100%, t_{ret}= 6.57 min, *m/z* 425.29 [M^++1]. **^{1}H NMR** (500 MHz, DMSO-d_6) δ 2.91 (s, 6H, CH_3), 6.67 (d, J = 9.0 Hz, 2H, Ar), 7.20 – 7.31 (m, 3H, Ar), 7.33 (dt, J = 8.9, 1.9 Hz, 2H, Ar), 7.57 (t, J = 7.3 Hz, 2H, Ar), 7.66 (tt, J = 7.5, 1.2 Hz, 1H, Ar), 8.01 (dd, J = 7.1, 1.4 Hz, 2H, Ar), 11.71 (br. s, 1H, NH), 12.39 (br. s, 1H, NH).

^{13}**C NMR** (126 MHz, DMSO-d_6) δ 86.8 (C≡C), 91.7 (C≡C), 108.5, 112.0, 112.4, 114.9, 118.1, 119.7, 125.2, 126.9, 128.1, 128.5, 129.3, 131.7, 133.0, 133.2, 133.4, 150.8, 153.9 (C=O), 154.6 (C=O), 165.8 (C=O).

N-(2,3-dioxo-6-(pyridin-4-ylethynyl)-3,4-dihydroquinoxalin-1(2H)-yl)benzamide **(25)**
Yield: 77%, mp. 239-241°C Anal. for $C_{22}H_{14}N_4O_3$ x 0.5 H_2O: Calc.: C, 67.51; H, 3.86; N, 14.32. Found: C, 67.67; H, 4.02; N, 14.11. **LC/MS**: purity 100%, t_{ret}= 3.59 min, *m/z* 383.20 [M^++1]. ^{1}**H NMR** (500 MHz, DMSO-d_6) δ 7.34 – 7.42 (m, 3H, Ar), 7.52 (d, *J* = 5.0 Hz, 2H, Ar), 7.57 (t, *J* = 7.6 Hz, 2H, Ar), 7.66 (t, *J* = 7.4 Hz, 1H, Ar), 8.01 (d, *J* = 7.6 Hz, 2H, Ar), 8.60 (d, *J* = 5.2 Hz, 2H, Ar), 11.81 (br. s, 1H, NH), 12.42 (br. s, 1H, NH). ^{13}**C NMR** (126 MHz, DMSO-d_6) δ 87.4 (C≡C), 93.1 (C≡C), 115.1, 117.4, 119.0, 125.3, 125.9, 127.8, 128.5, 129.3, 129.5, 130.5, 131.6, 133.4, 150.5, 154.0 (C=O), 154.5 (C=O), 165.9 (C=O).

N-(6-((6-hydroxypyridin-3-yl)ethynyl)-2,3-dioxo-3,4-dihydroquinoxalin-1(2H)-yl)benzamide **(26)**
Yield: 64%, mp. 273-275°C Anal. for $C_{22}H_{14}N_4O_4$ x 1 H_2O: Calc.: C, 63.46; H, 3.87; N, 13.46. Found: C, 63.54; H, 4.02; N, 13.67. **LC/MS**: purity 96.11%, t_{ret}= 3.85 min, *m/z* 399.15 [M^++1]. ^{1}**H NMR** (500 MHz, DMSO-d_6) δ 6.33 (d, *J* = 9.5 Hz, 1H, Ar), 7.23 – 7.33 (m, 3H, Ar), 7.51 (dd, *J* = 9.5, 2.6 Hz, 1H, Ar), 7.56 (t, *J* = 7.6 Hz, 2H, Ar), 7.66 (t, *J* = 7.4 Hz, 1H, Ar), 7.76 (d, *J* = 2.6 Hz, 1H, Ar), 8.00 (d, *J* = 7.1 Hz, 2H, Ar), 11.71 (s, 1H, NH), 11.97 (br. s, 1H, OH), 12.43 (s, 1H, NH). ^{13}**C NMR** (126 MHz, DMSO-d_6) δ 86.7 (C≡C), 88.5 (C≡C), 100.6, 115.0, 118.3, 118.8, 120.7, 125.2, 127.1, 128.5, 128.5, 129.3, 131.6, 133.4, 140.6, 142.9, 143.1, 153.9 (C=O), 154.6 (C=O), 161.7 (C_{Ar}-OH), 165.8 (C=O).

N-(2,3-dioxo-6-(pyrimidin-5-ylethynyl)-3,4-dihydroquinoxalin-1(2H)-yl)benzamide **(27)**
Yield: 74%, mp. 318-320°C Anal. for $C_{21}H_{13}N_5O_3$ x 1.5 H_2O: Calc.: C, 61.46; H, 3.93; N, 17.07. Found: C, 61.68; H, 3.73; N, 17.21. **LC/MS**: purity 100%, t_{ret}= 4.40 min, *m/z* 384.20 [M^++1]. ^{1}**H NMR** (500 MHz, DMSO-d_6) δ 7.32 – 7.41 (m, 2H, Ar), 7.48 (d, *J* = 1.1 Hz, 1H, Ar), 7.55 (t, *J* = 7.4 Hz, 2H, Ar), 7.65 (tt, *J* = 7.4, 1.1 Hz, 1H, Ar), 8.04 (dd, *J* = 7.0, 1.5 Hz, 2H, Ar), 9.01 (s, 2H, Ar), 9.16 (s, 1H, Ar). ^{13}**C NMR** (126 MHz, DMSO-d_6) δ 83.6 (C≡C), 95.1 (C≡C), 115.1, 117.4, 118.8, 125.3, 127.6, 128.5, 129.3, 129.4, 131.6, 133.4, 154.0 (C=O), 154.5 (C=O), 157.5, 159.3, 165.9 (C=O).

N-(6-(imidazo[1,2-b]pyridazin-3-ylethynyl)-2,3-dioxo-3,4-dihydroquinoxalin-1(2H)-yl)benzamide **(28)**

Yield: 79%, mp. 314-315°C Anal. for $C_{23}H_{14}N_6O_3$ x 1 H_2O: Calc.: C, 62.73; H, 3.66; N, 19.08. Found: C, 62.94; H, 3.84; N, 18.98. **LC/MS**: purity 100%, t_{ret}= 4.40 min, *m/z* 423.08 [M^++1]. ^{1}**H NMR** (500 MHz, DMSO-d_6) δ 7.33 – 7.39 (m, 3H, Ar), 7.41 (s, 1H, Ar), 7.57 (t, *J* = 7.6 Hz, 2H, Ar), 7.67 (t, *J* = 7.4 Hz, 1H, Ar), 8.01 (d, *J* = 7.2 Hz, 2H, Ar), 8.22 (d, *J* = 9.2 Hz, 2H, Ar), 8.67 (d, *J* = 4.4 Hz, 1H, Ar), 11.75 (s, 1H, NH), 12.45 (s, 1H, NH). ^{13}**C NMR** (126 MHz, DMSO-d_6) δ 77.4 (C≡C), 97.7 (C≡C), 112.1, 115.1, 118.0, 118.4, 119.6, 125.4, 126.6, 127.1, 128.5, 129.1, 129.3, 131.6, 133.4, 139.0, 140.1, 145.5, 154.0 (C=O), 154.5 (C=O), 165.9 (C=O).

N-(6-((1H-pyrazolo[4,3-b]pyridin-5-yl)ethynyl)-2,3-dioxo-3,4-dihydroquinoxalin-1(2H)-yl)benzamide **(29)**

Yield: 65%, mp. Over 350°C Anal. for $C_{23}H_{14}N_6O_3$ x 0.5 H_2O: Calc.: C, 64.03; H, 3.50; N, 19.48. Found: C, 63.96; H, 3.51; N, 19.38. **LC/MS**: purity 100%, t_{ret}= 4.31 min, *m/z* 423.14 [M^++1]. ^{1}**H NMR** (500 MHz, DMSO-d_6) δ 7.34 – 7.44 (m, 3H, Ar), 7.54 – 7.62 (m, 3H, Ar), 7.67 (tt, *J* = 7.5, 1.2 Hz, 1H, Ar), 7.99 – 8.07 (m, 3H, Ar), 8.29 (s, 1H, Ar), 11.75 (s, 1H, NH), 12.48 (s, 1H, NH), 13.50 (s, 1H, NH). ^{13}**C NMR** (126 MHz, DMSO-d_6) δ 86.8 (C≡C), 90.3 (C≡C), 115.1 (C≡C), 117.8, 118.9, 119.6, 124.9, 125.3, 127.7, 128.5, 129.2, 129.3, 131.6, 131.8, 133.4, 134.6, 137.7, 141.7, 150.5, 154.0 (C=O), 154.5 (C=O), 165.9 (C=O).

Supplementary Figures

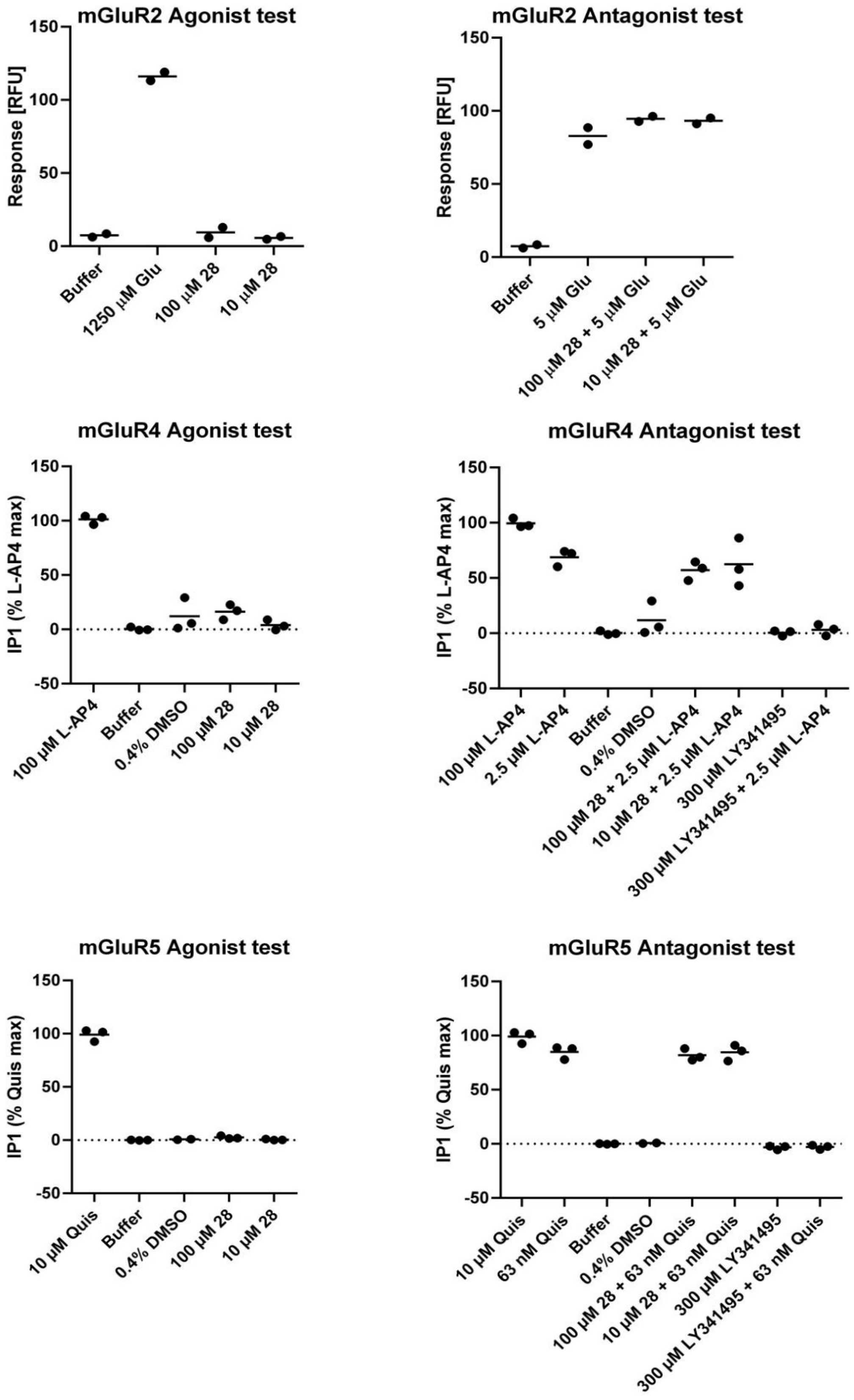

Figure S1. Functional testing of compound **28** at mGluR2,4,5. **28** exhibited neither agonistic nor antagonistic effects at the tested mGluR subtypes. Quis = quisqualic acid; Glu = L-glutamate.

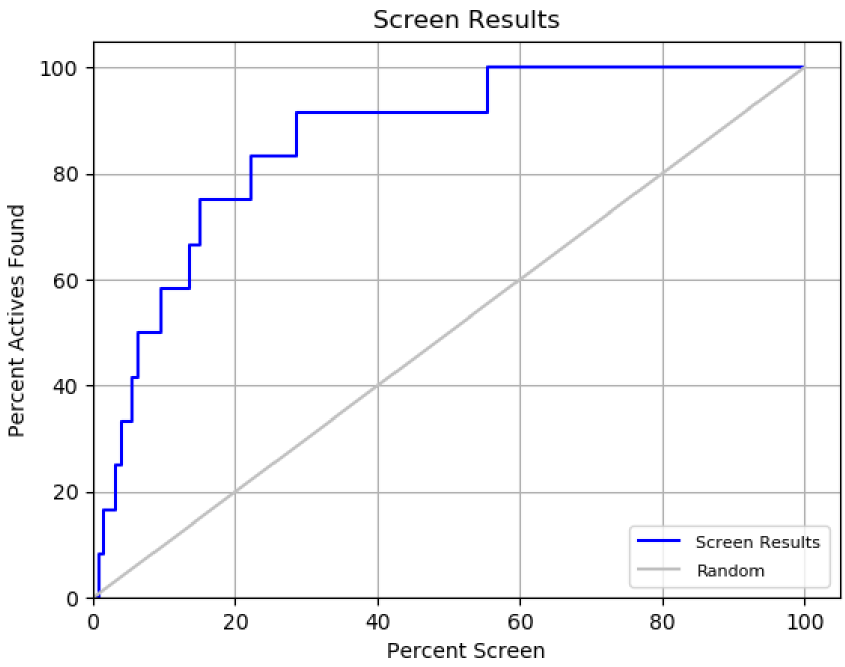

Figure S2. Enrichment plot for the GluK3-LBD homology mode built with the Modeller program, using the 6SBT crystal structure as a template.

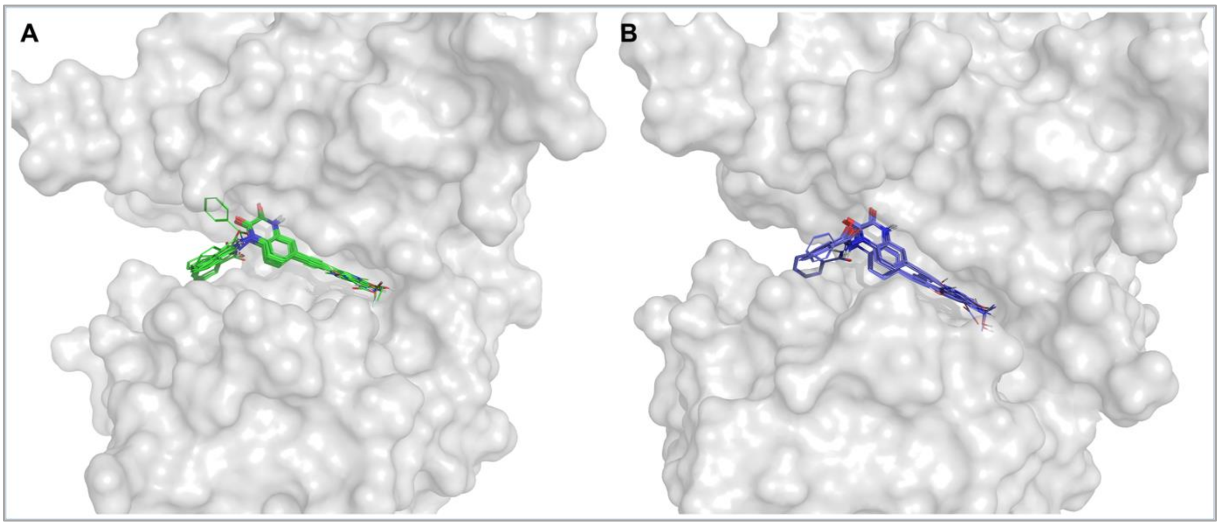

Figure S3. Comparison of the binding modes for the top-ranked docking poses of **5-29** in the GluK1-LBD X-ray structure (PDB code: 6SBT) (**A**) and the GluK3-LBD homology model (**B**).

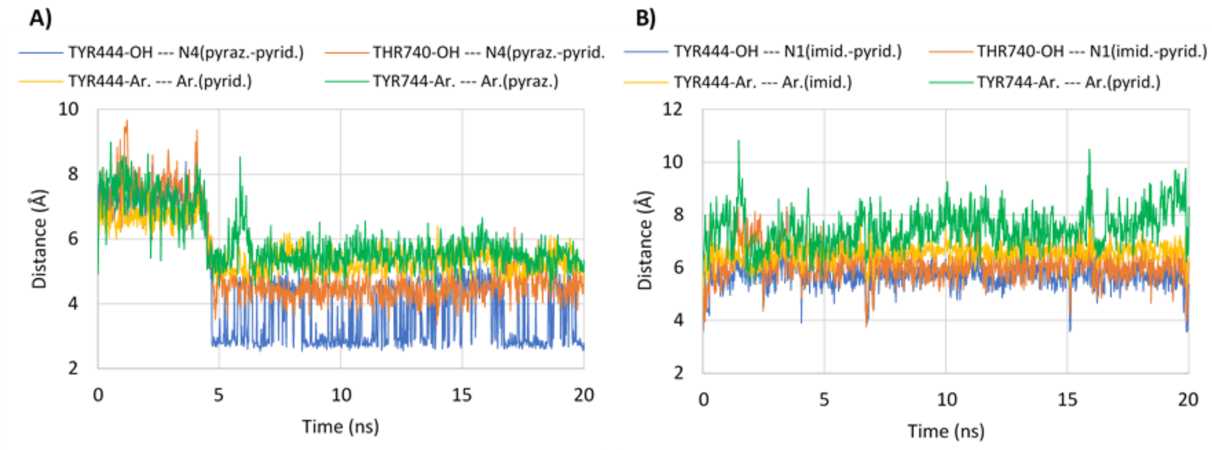

Figure S4. Distance changes between amino acid residues of the GluK1-LBD and nitrogen atoms/aromatic rings of the substituent at position 6 of compound **29** (**A**) and **28** (**B**), engaged in key interactions, during 20 ns molecular dynamics simulation. The distances between aromatic fragments were measured between the centroids of the indicated rings.

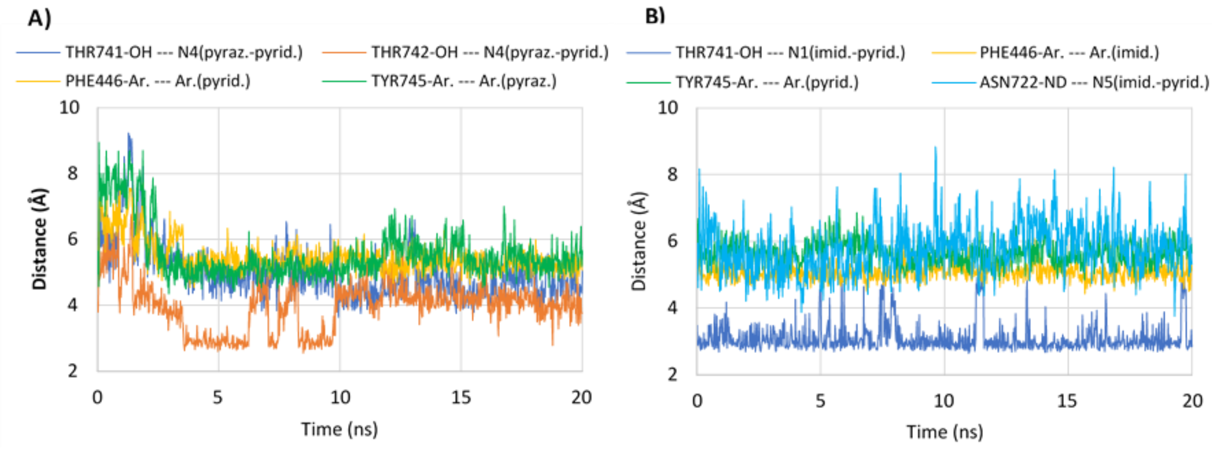

Figure S5. Changes in distance between amino acid residues of GluK3-LBD and nitrogen atoms/aromatic rings of the substituent at position 6 of compound **29** (**A**) and **28** (**B**), engaged in key interactions, during a 20 ns molecular dynamics simulation. The distances between aromatic fragments were measured between the centroids of the indicated rings.

NMR Spectra

N-(2,3-dioxo-6-(*m*-tolylethynyl)-3,4-dihydroquinoxalin-1(2*H*)-yl)benzamide (**5**)

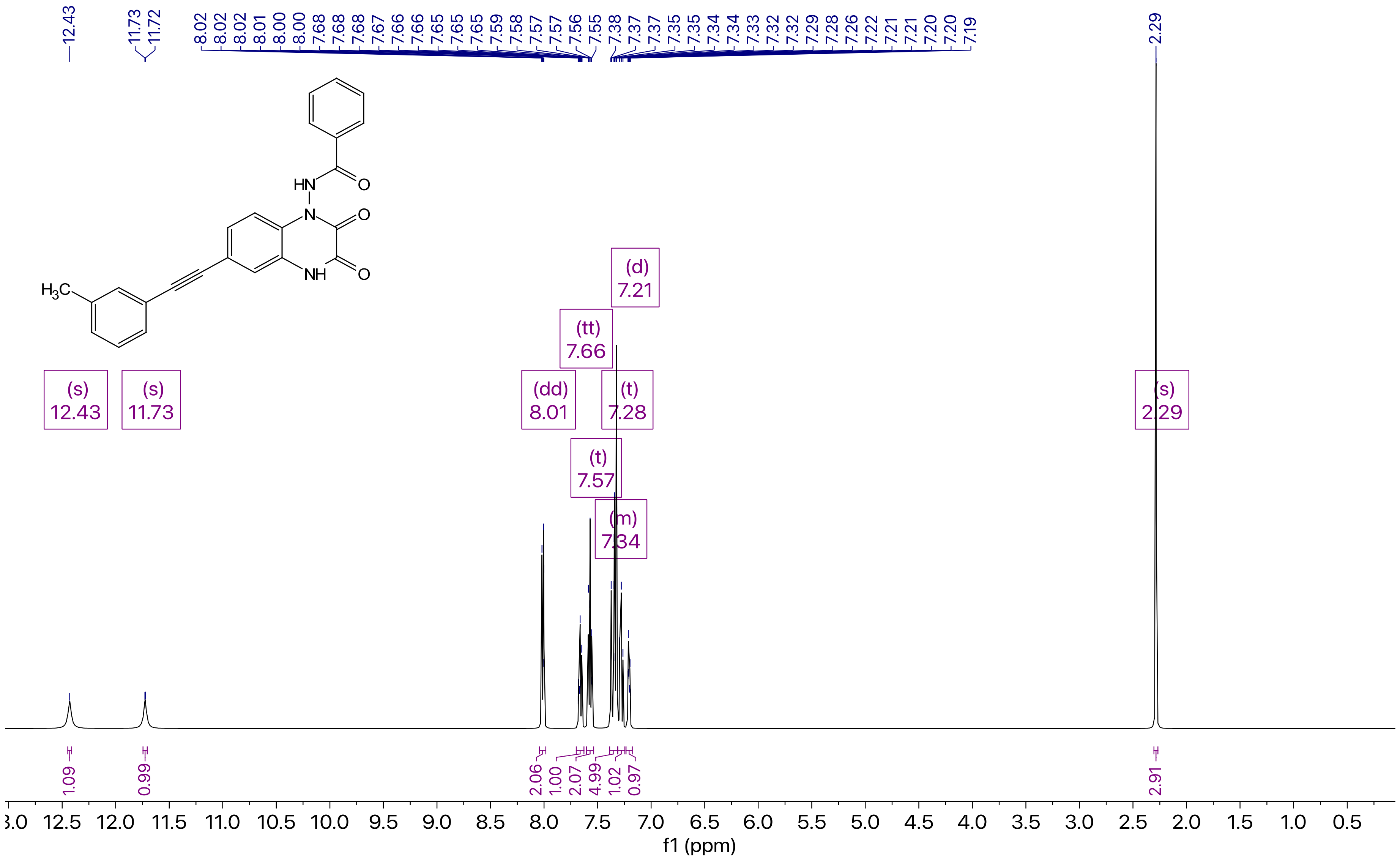

N-(2,3-dioxo-6-(*m*-tolylethynyl)-3,4-dihydroquinoxalin-1(2*H*)-yl)benzamide (**5**)

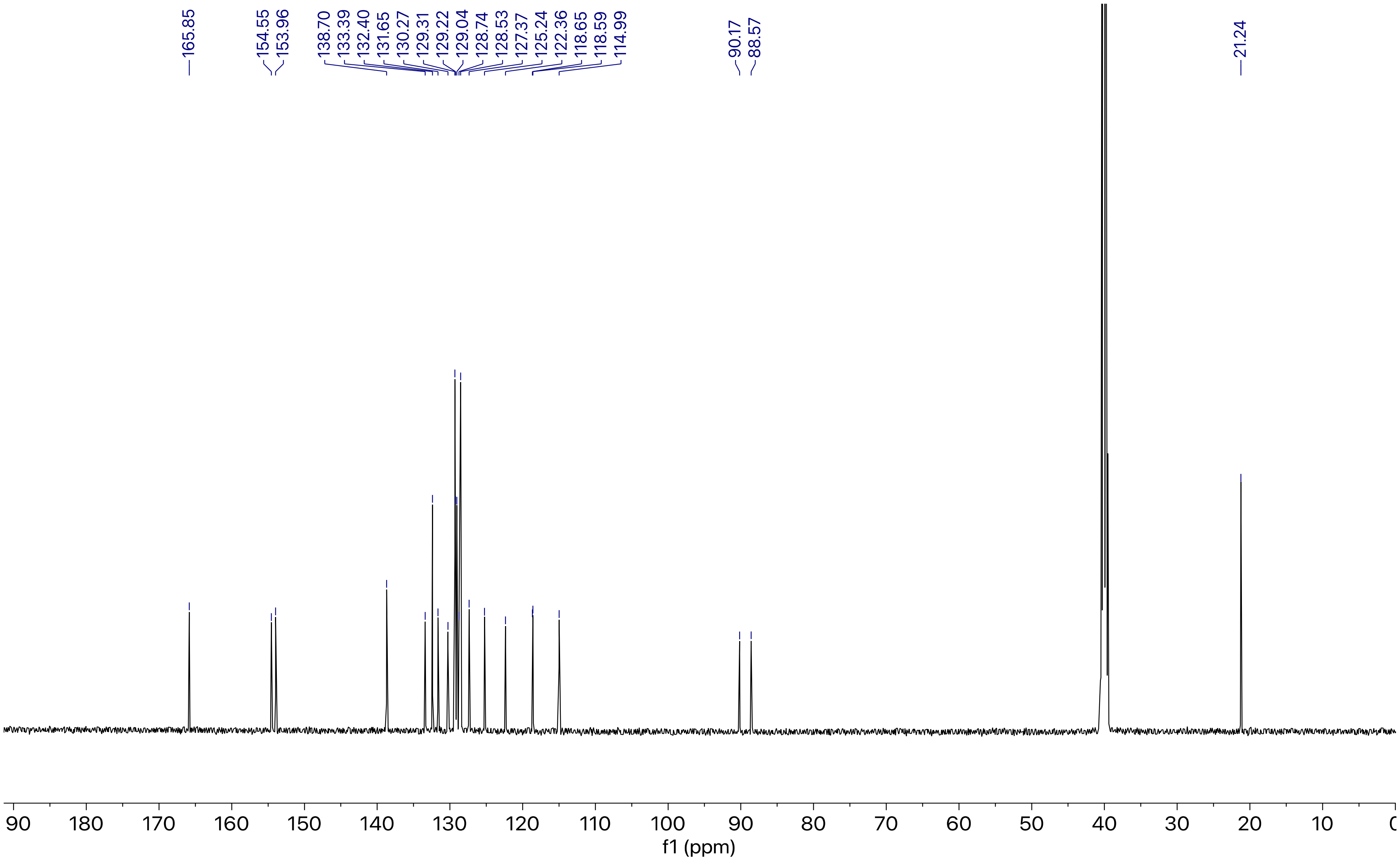

N-(2,3-dioxo-6-(*p*-tolylethynyl)-3,4-dihydroquinoxalin-1(2*H*)-yl)benzamide (**6**)

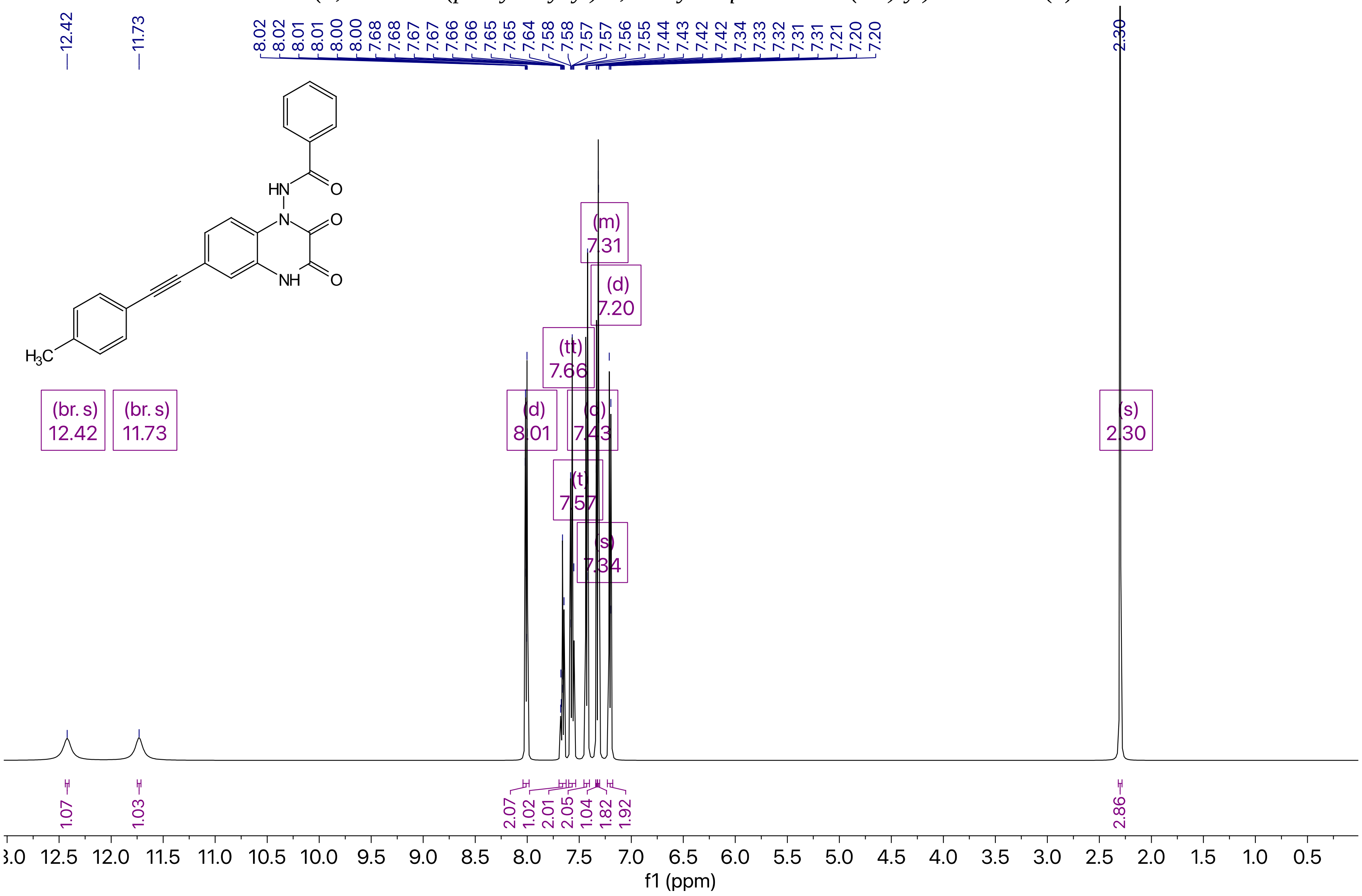

N-(2,3-dioxo-6-(p-tolylethynyl)-3,4-dihydroquinoxalin-1(2H)-yl)benzamide (**6**)

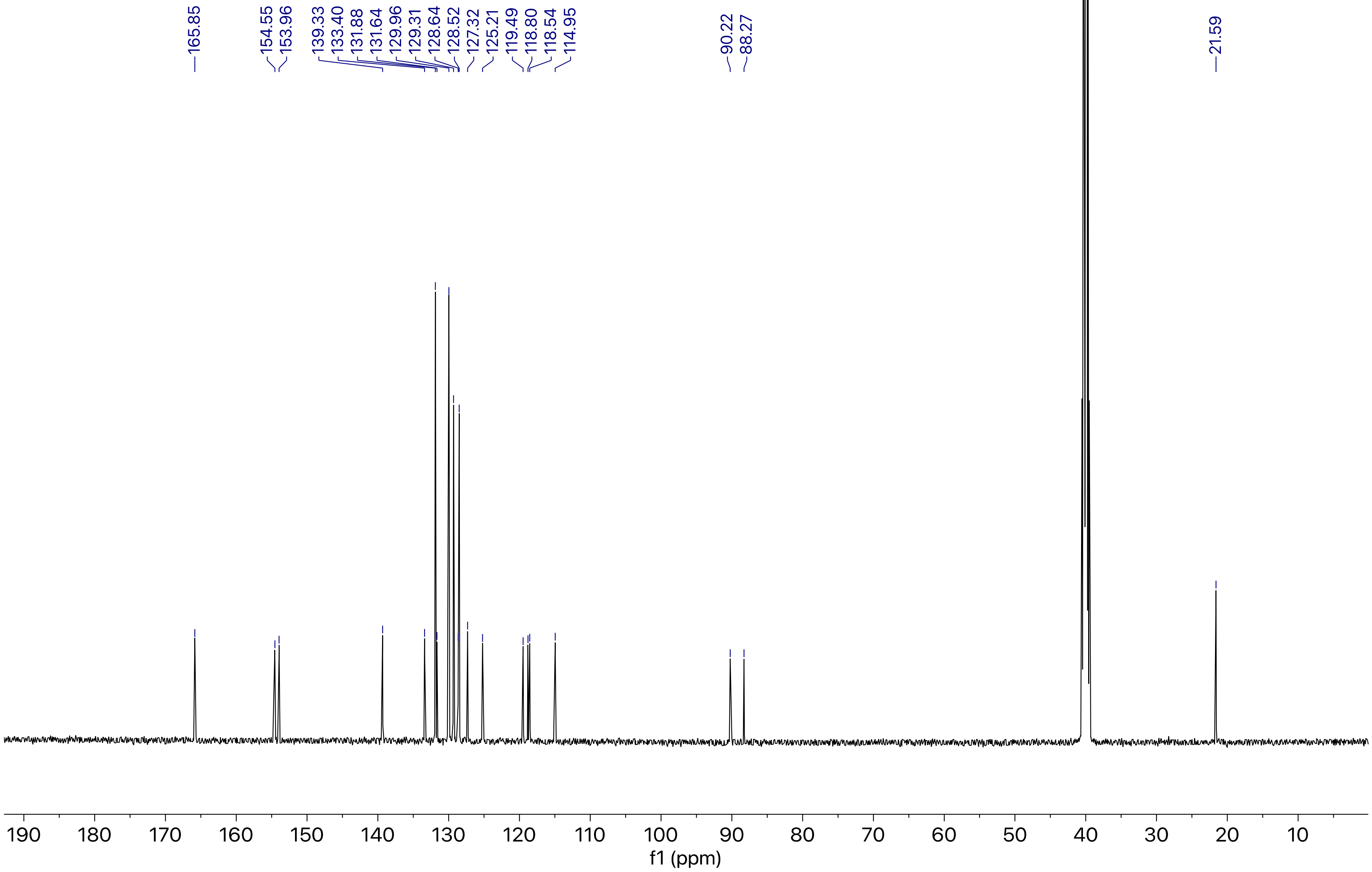

N-(6-((4-ethylphenyl)ethynyl)-2,3-dioxo-3,4-dihydroquinoxalin-1(2H)-yl)benzamide (**7**)

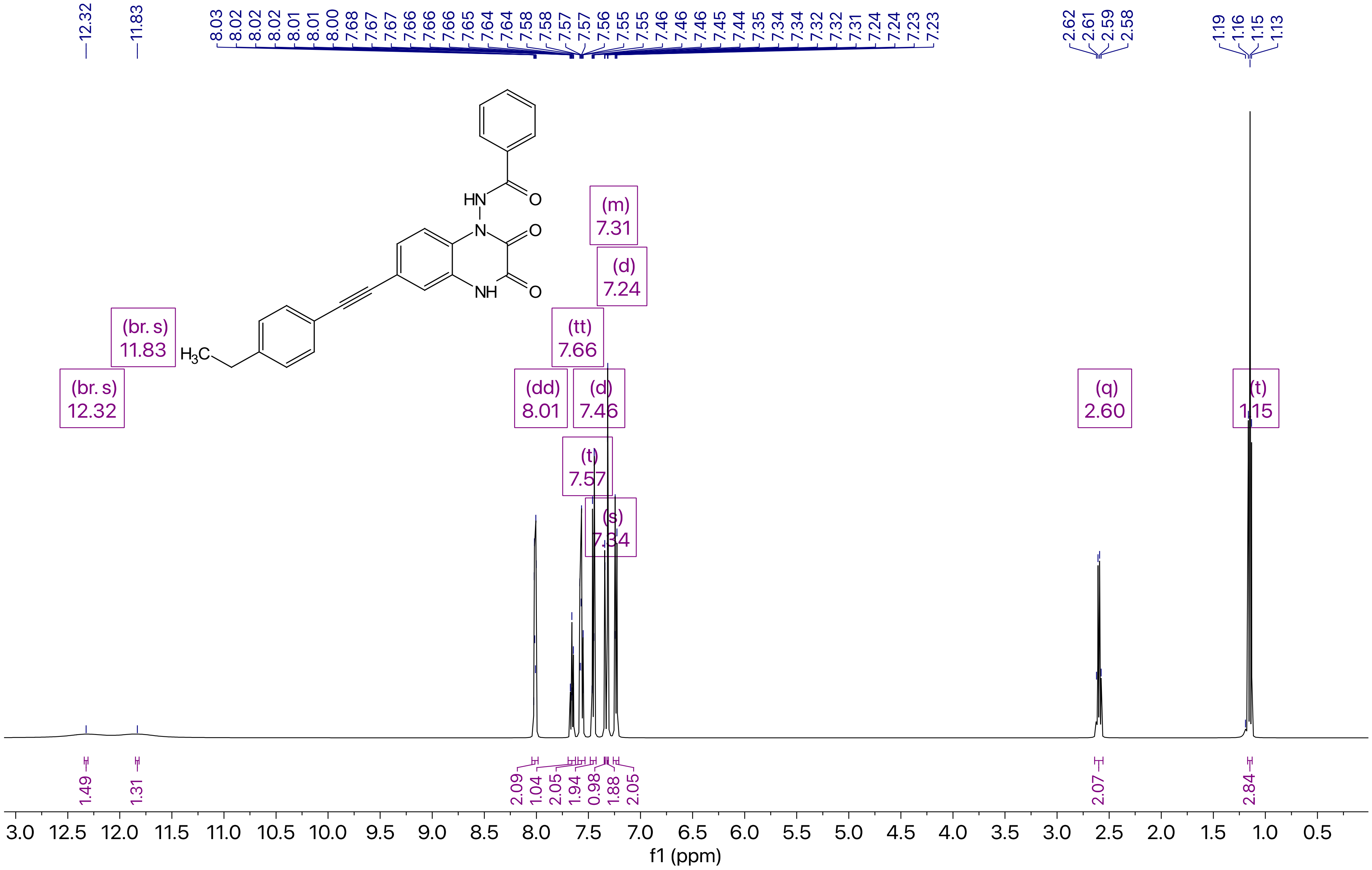

N-(6-((4-ethylphenyl)ethynyl)-2,3-dioxo-3,4-dihydroquinoxalin-1(2H)-yl)benzamide (**7**)

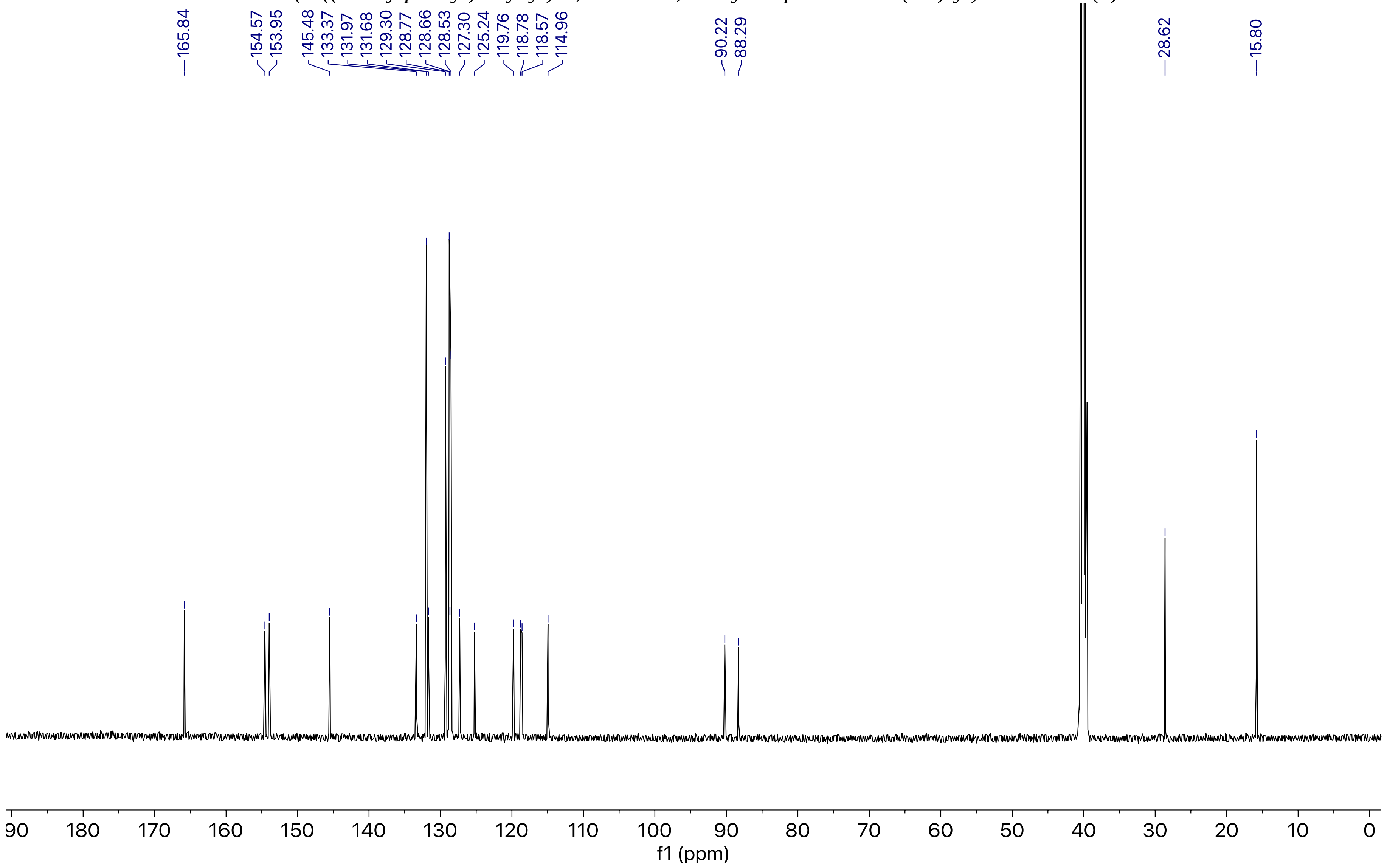

N-(2,3-dioxo-6-((4-propylphenyl)ethynyl)-3,4-dihydroquinoxalin-1(2H)-yl)benzamide (**8**)

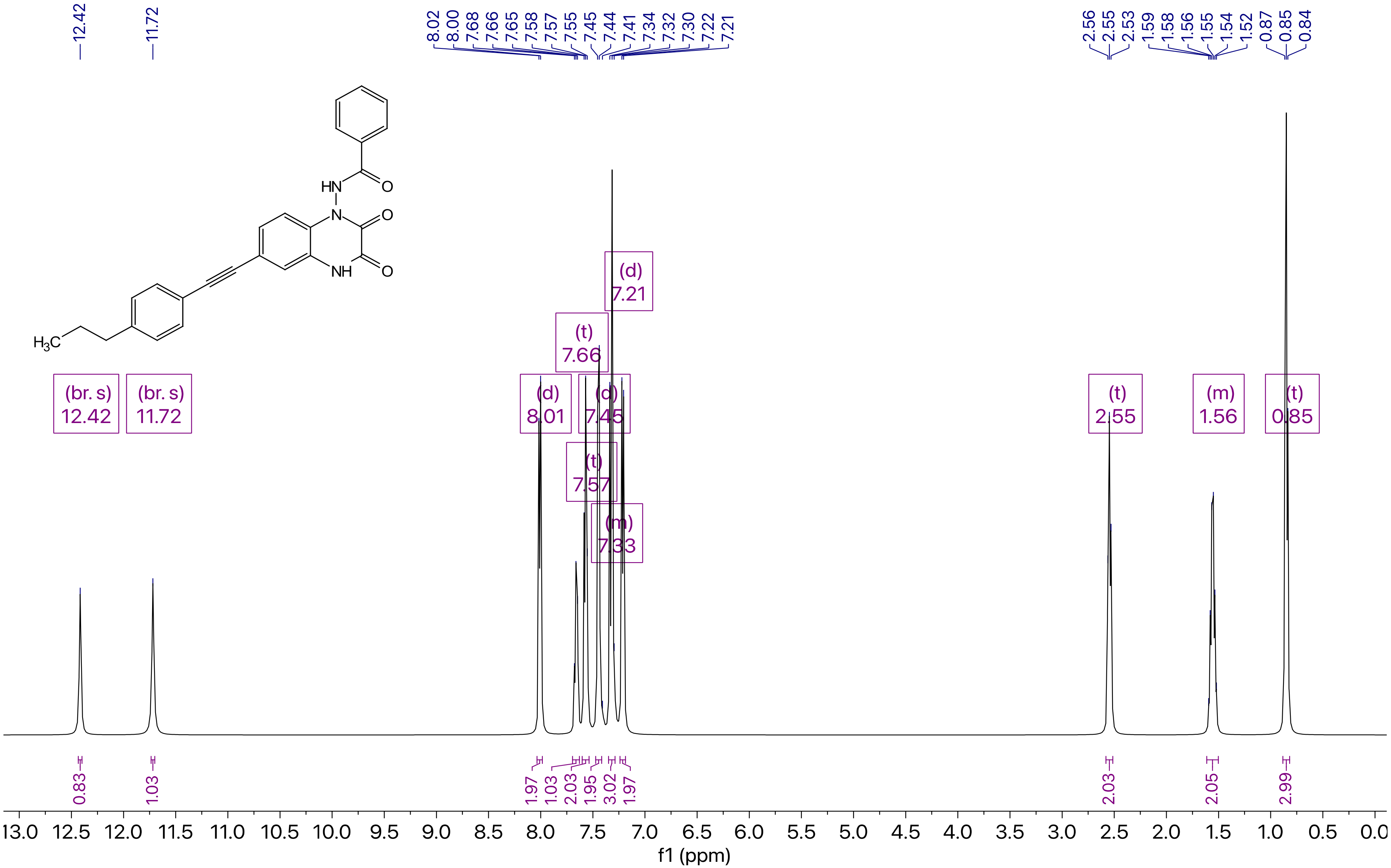

N-(2,3-dioxo-6-((4-propylphenyl)ethynyl)-3,4-dihydroquinoxalin-1(2*H*)-yl)benzamide (**8**)

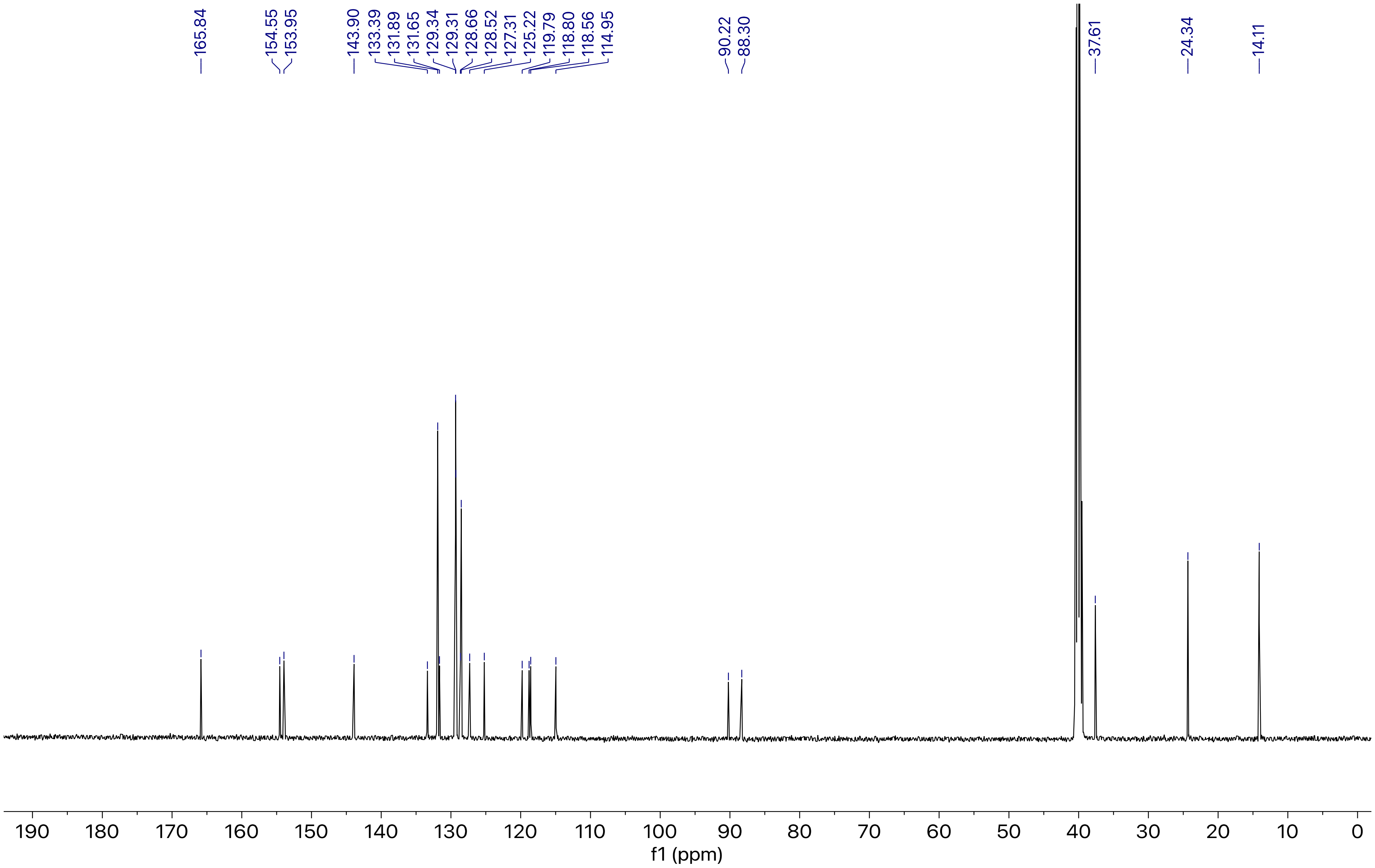

N-(6-((4-isopropylphenyl)ethynyl)-2,3-dioxo-3,4-dihydroquinoxalin-1(2*H*)-yl)benzamide (**9**)

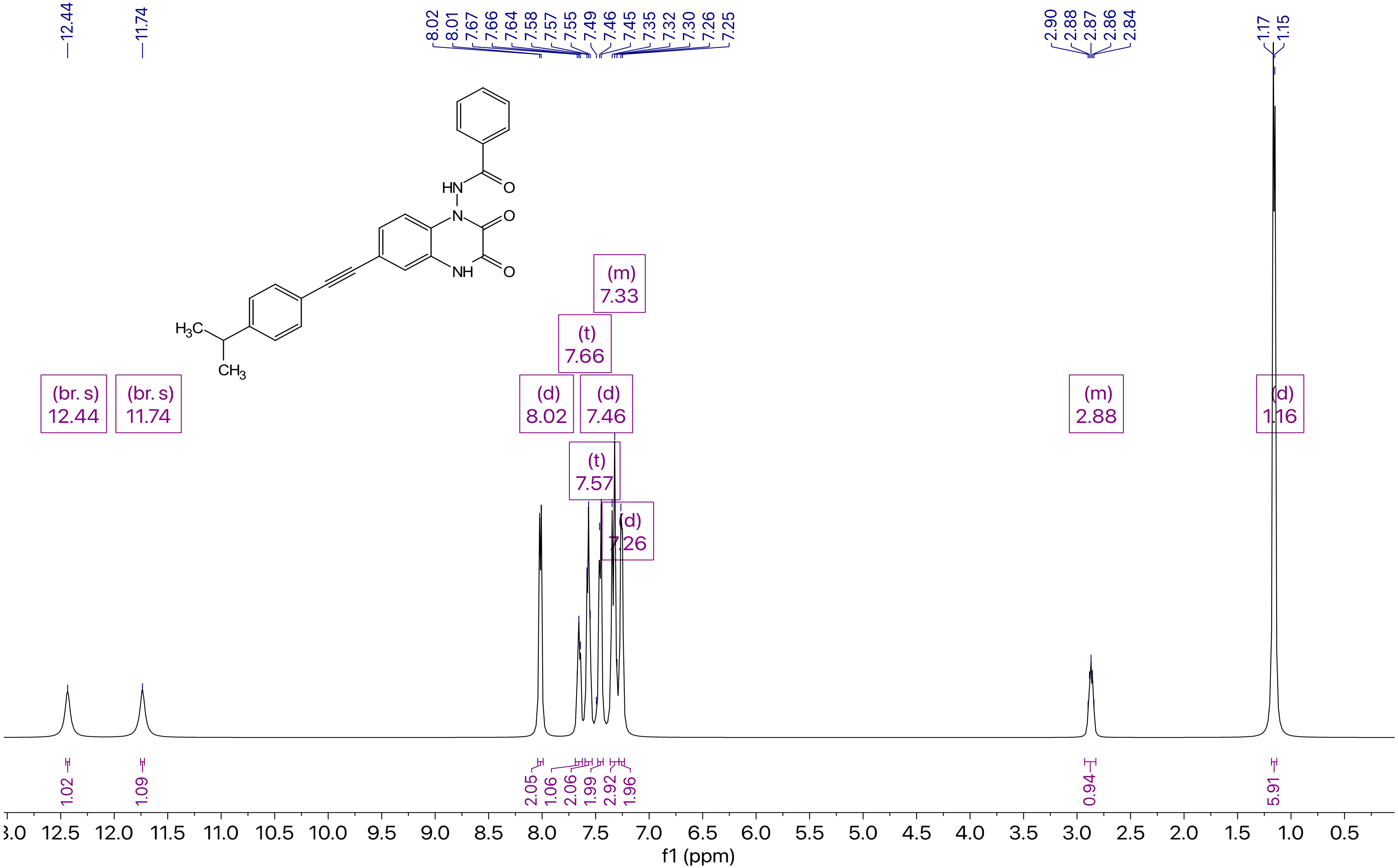

N-(6-((4-isopropylphenyl)ethynyl)-2,3-dioxo-3,4-dihydroquinoxalin-1(2H)-yl)benzamide (**9**)

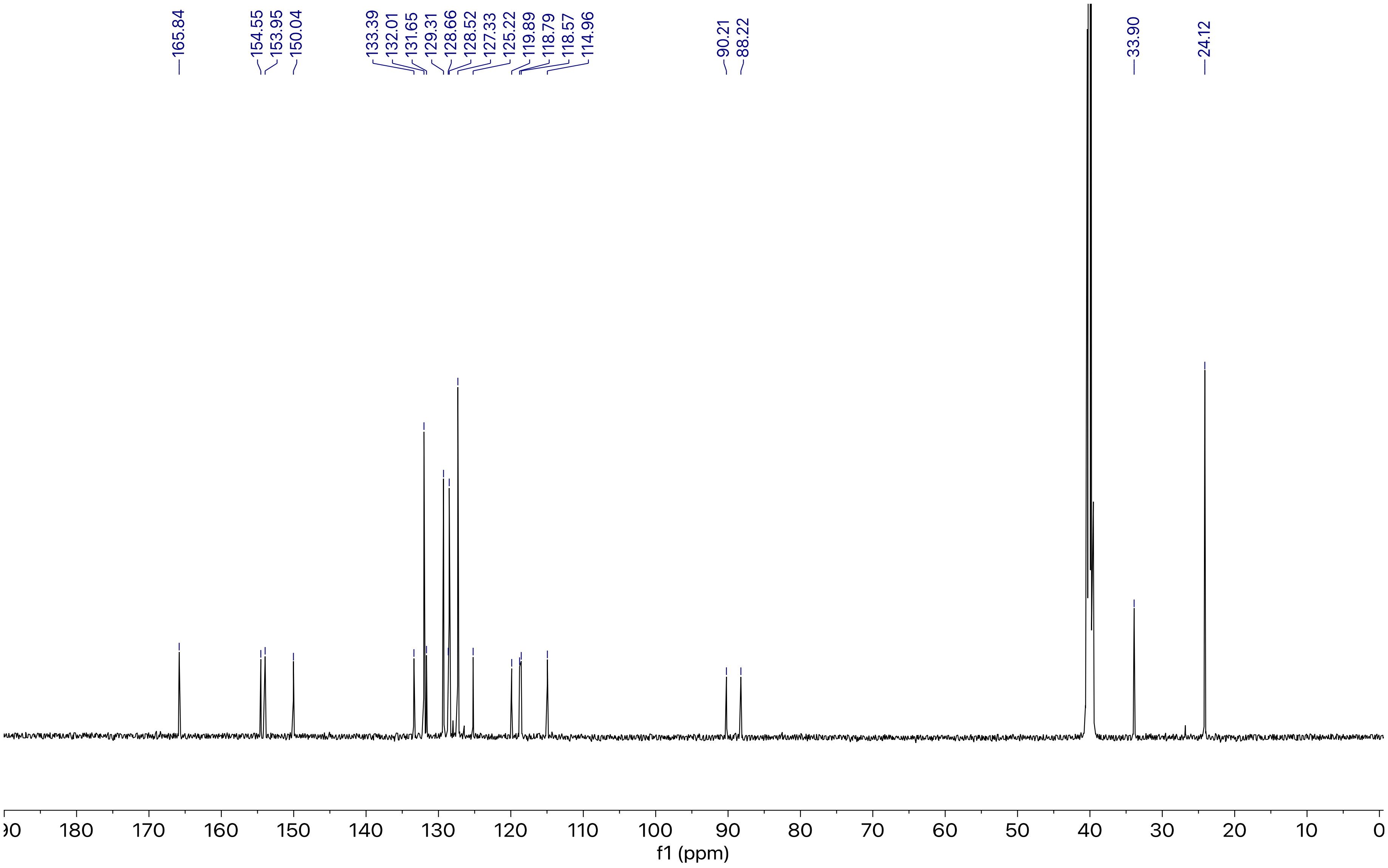

N-(6-((4-chlorophenyl)ethynyl)-2,3-dioxo-3,4-dihydroquinoxalin-1(2*H*)-yl)benzamide (**10**)

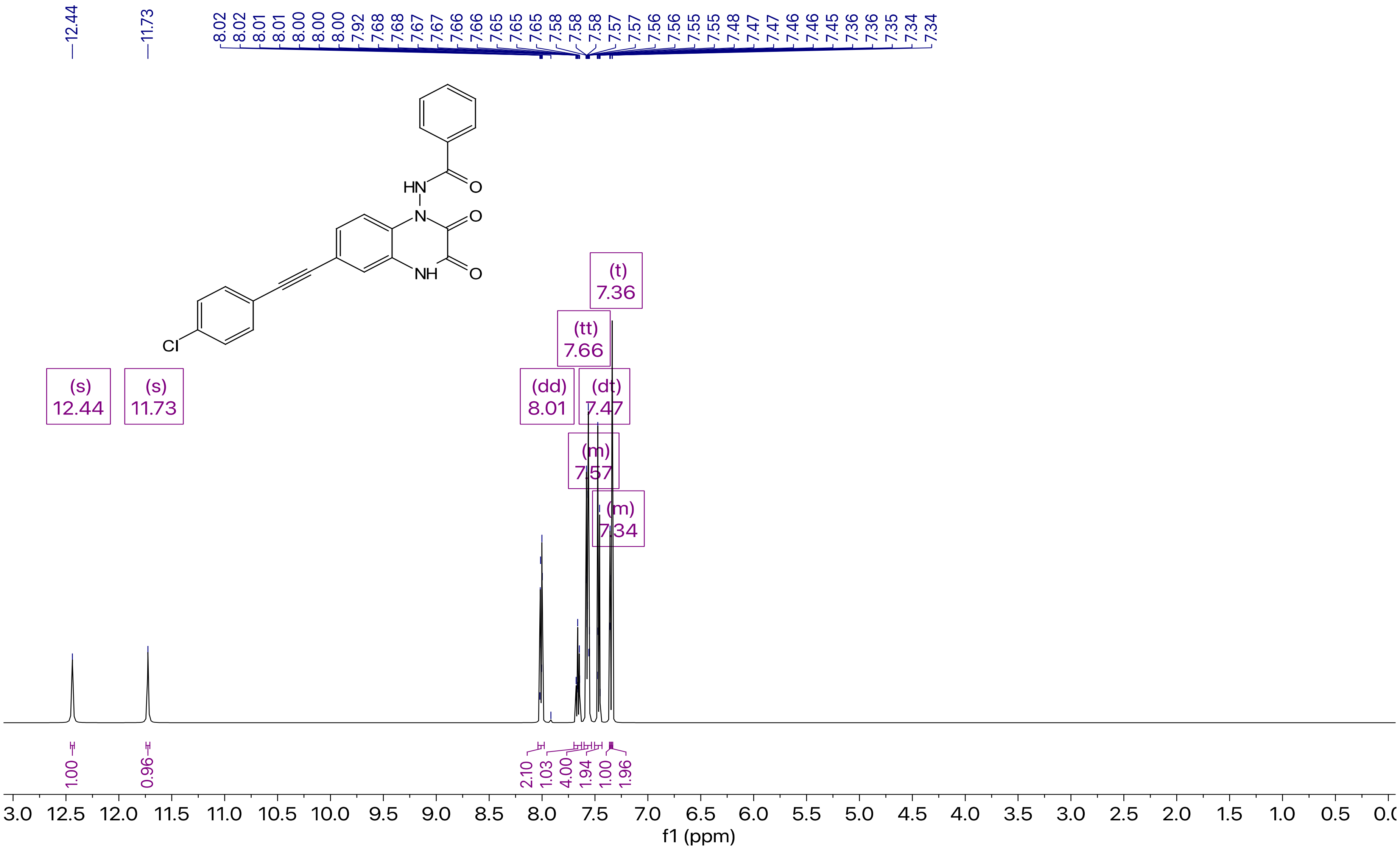

N-(6-((4-chlorophenyl)ethynyl)-2,3-dioxo-3,4-dihydroquinoxalin-1(2*H*)-yl)benzamide (**10**)

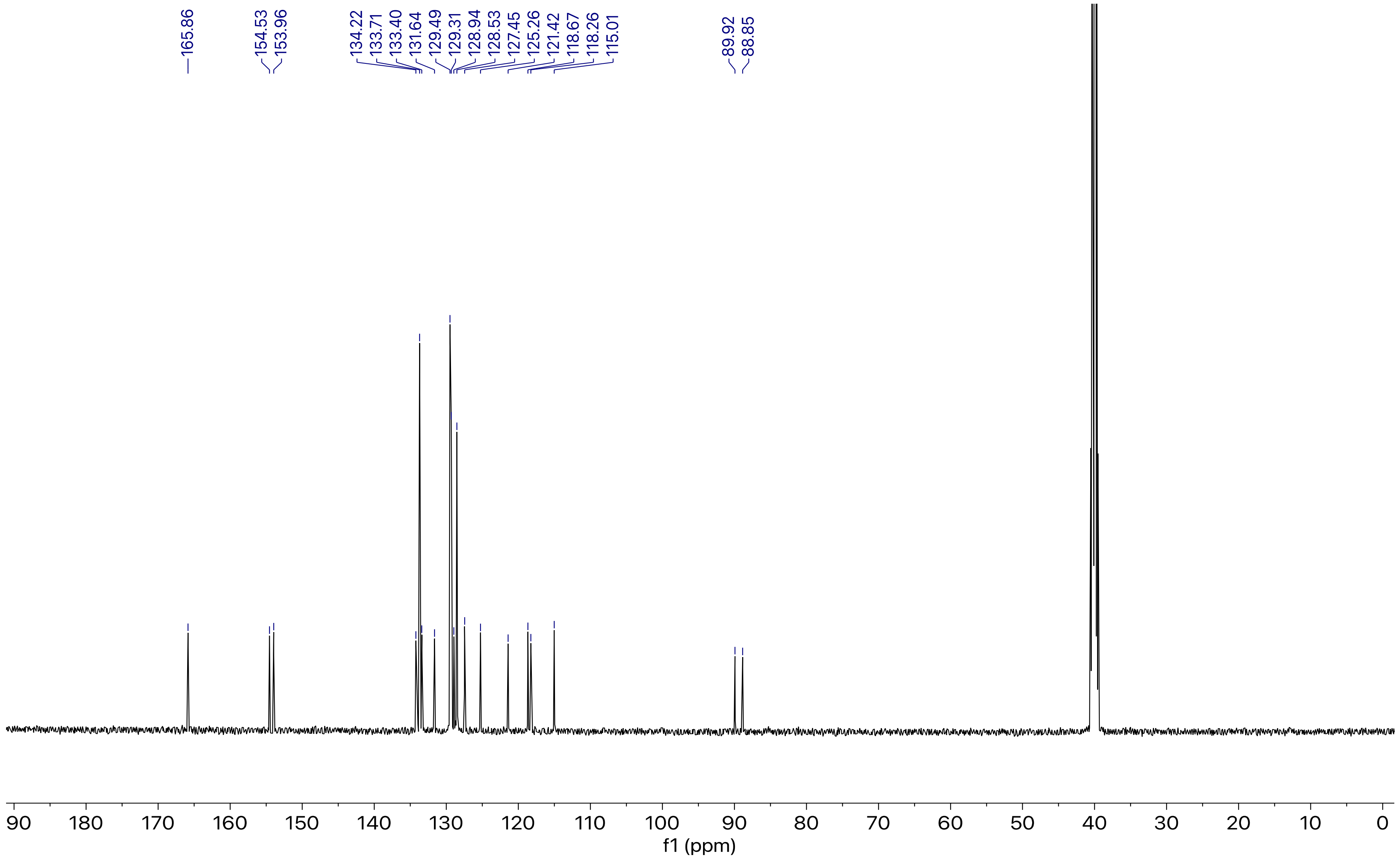

N-(2,3-dioxo-6-((4-(trifluoromethyl)phenyl)ethynyl)-3,4-dihydroquinoxalin-1(2*H*)-yl)benzamide (**11**)

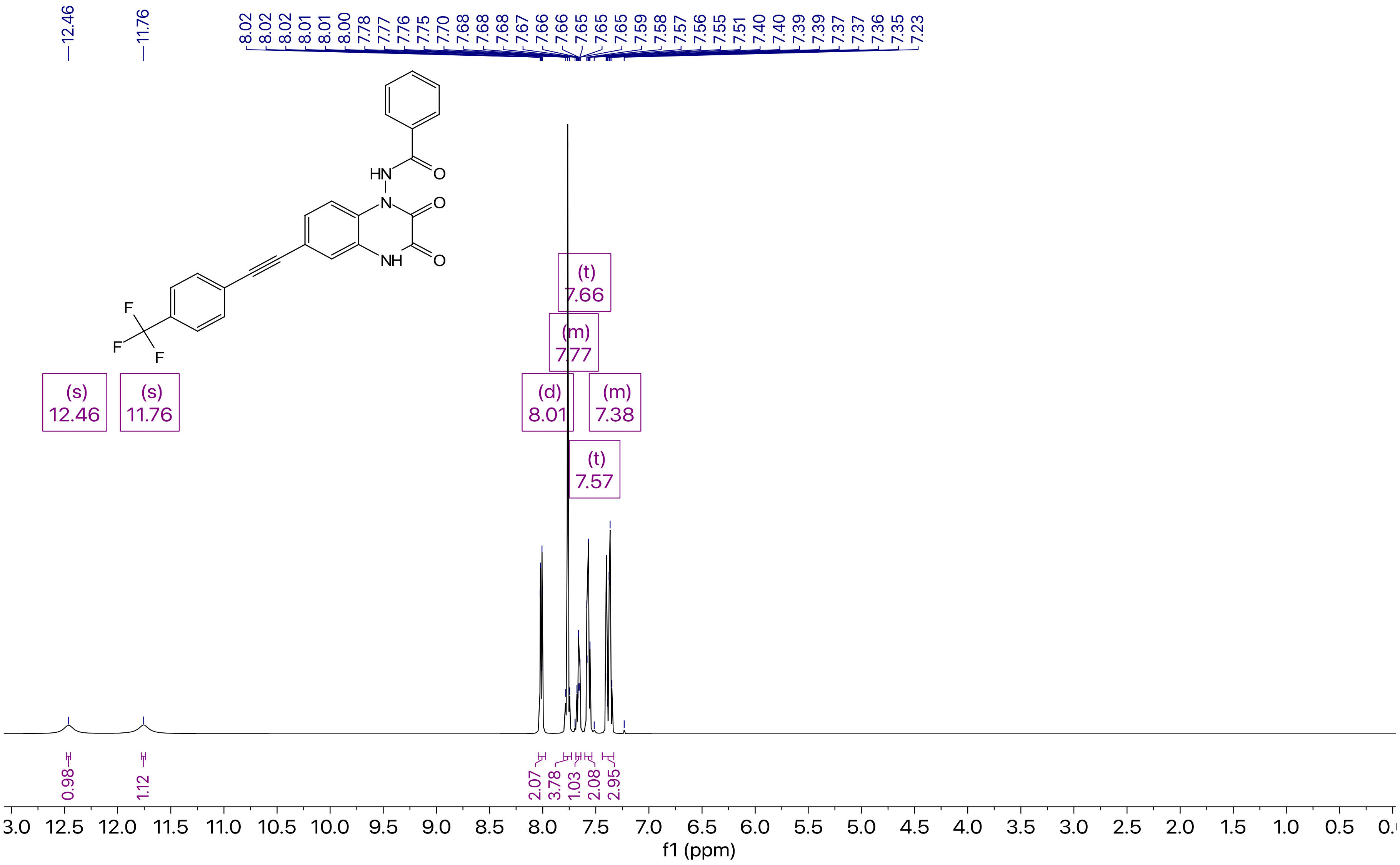

N-(2,3-dioxo-6-((4-(trifluoromethyl)phenyl)ethynyl)-3,4-dihydroquinoxalin-1(2H)-yl)benzamide (**11**)

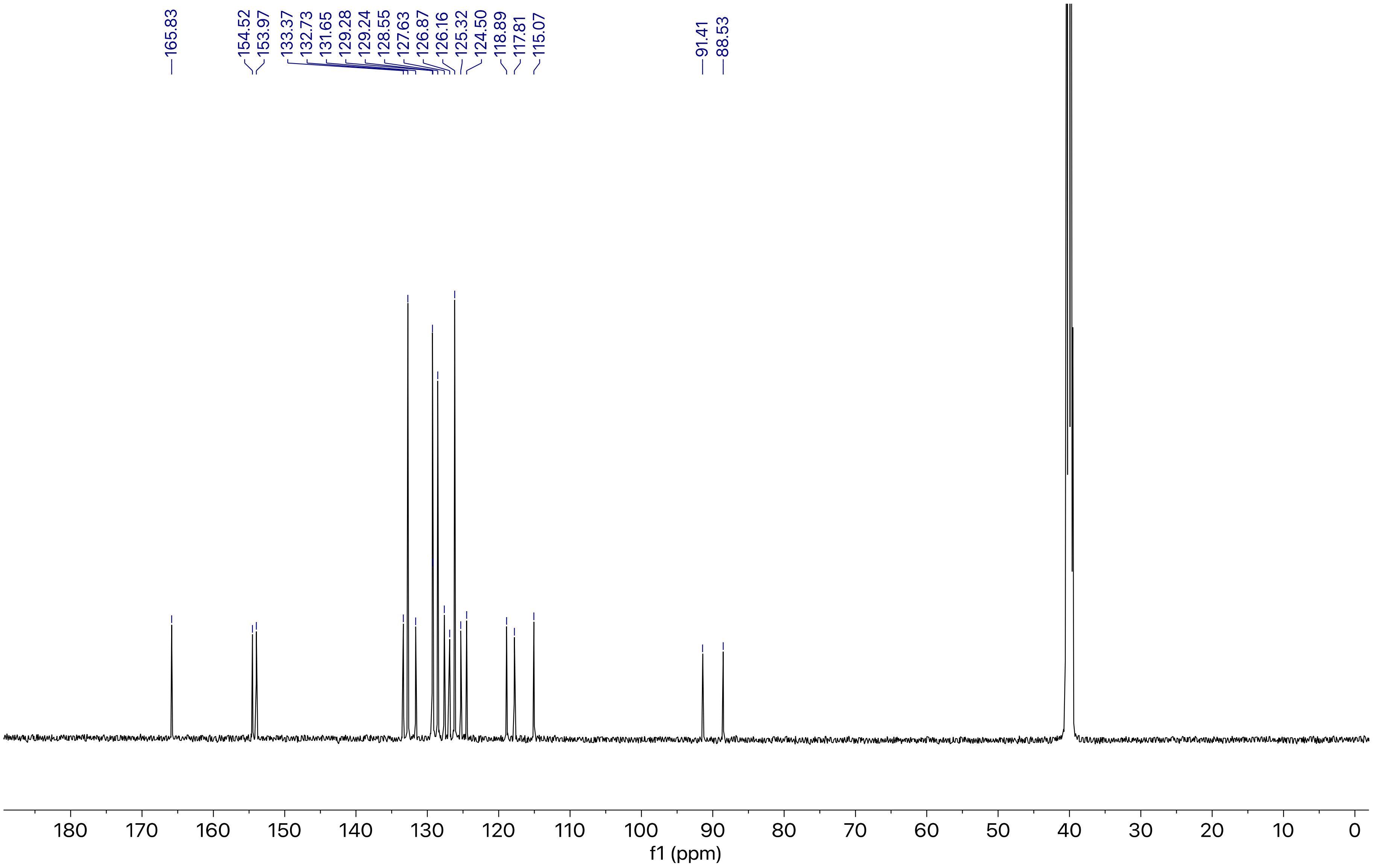

N-(6-((4-methoxyphenyl)ethynyl)-2,3-dioxo-3,4-dihydroquinoxalin-1(2H)-yl)benzamide (**12**)

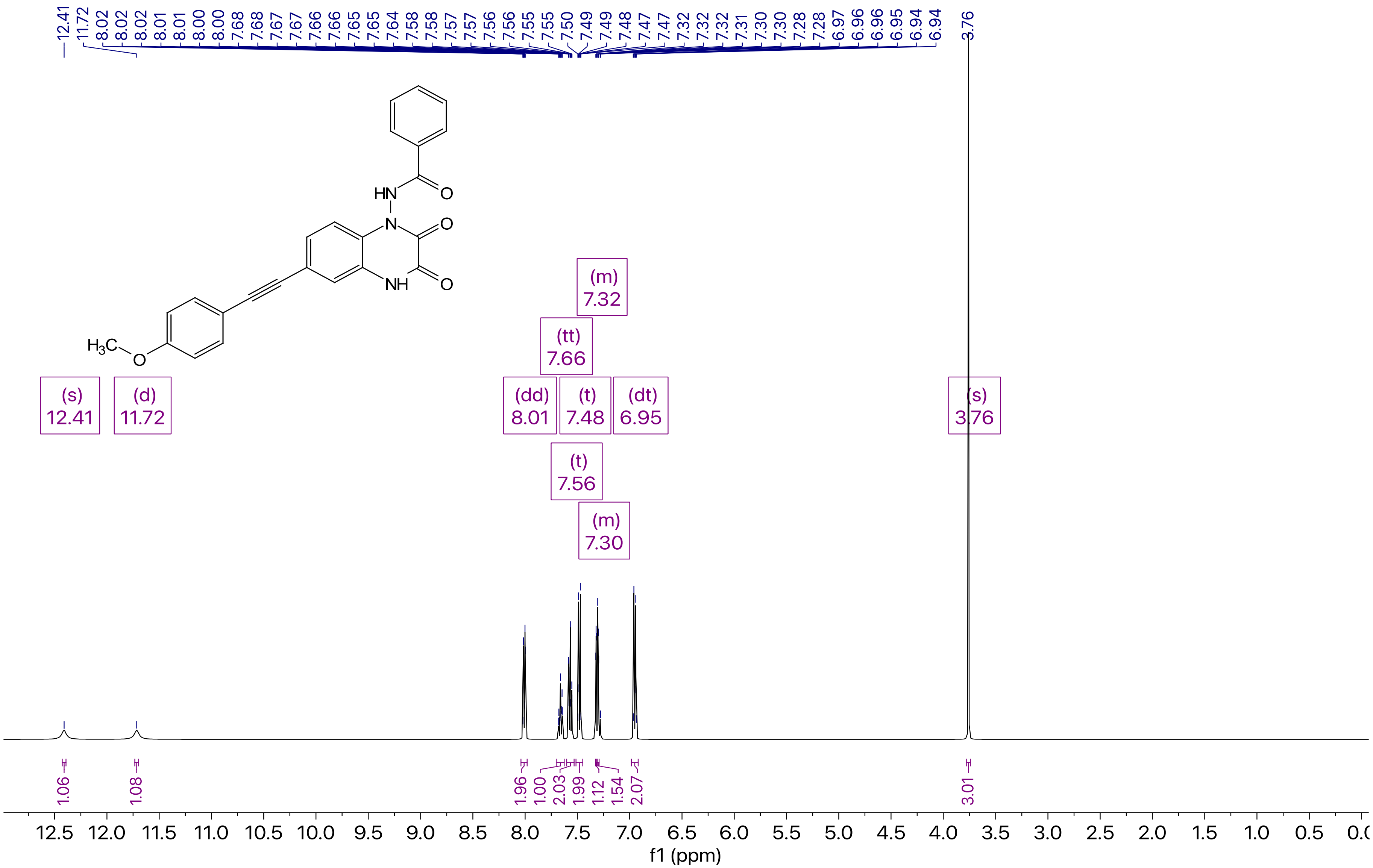

N-(6-((4-methoxyphenyl)ethynyl)-2,3-dioxo-3,4-dihydroquinoxalin-1(2H)-yl)benzamide (**12**)

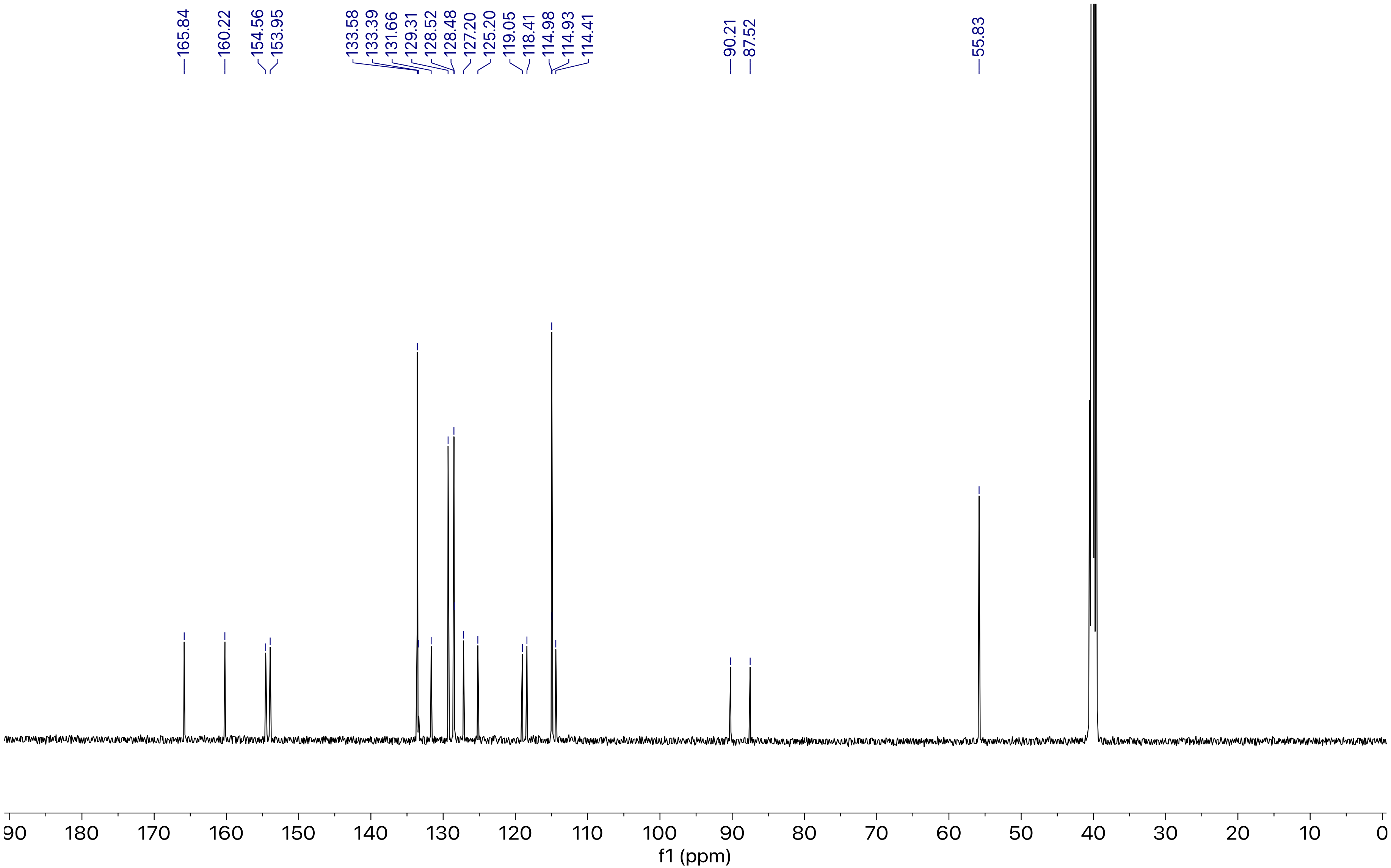

N-(2,3-dioxo-6-((4-phenoxyphenyl)ethynyl)-3,4-dihydroquinoxalin-1(2*H*)-yl)benzamide (**13**)

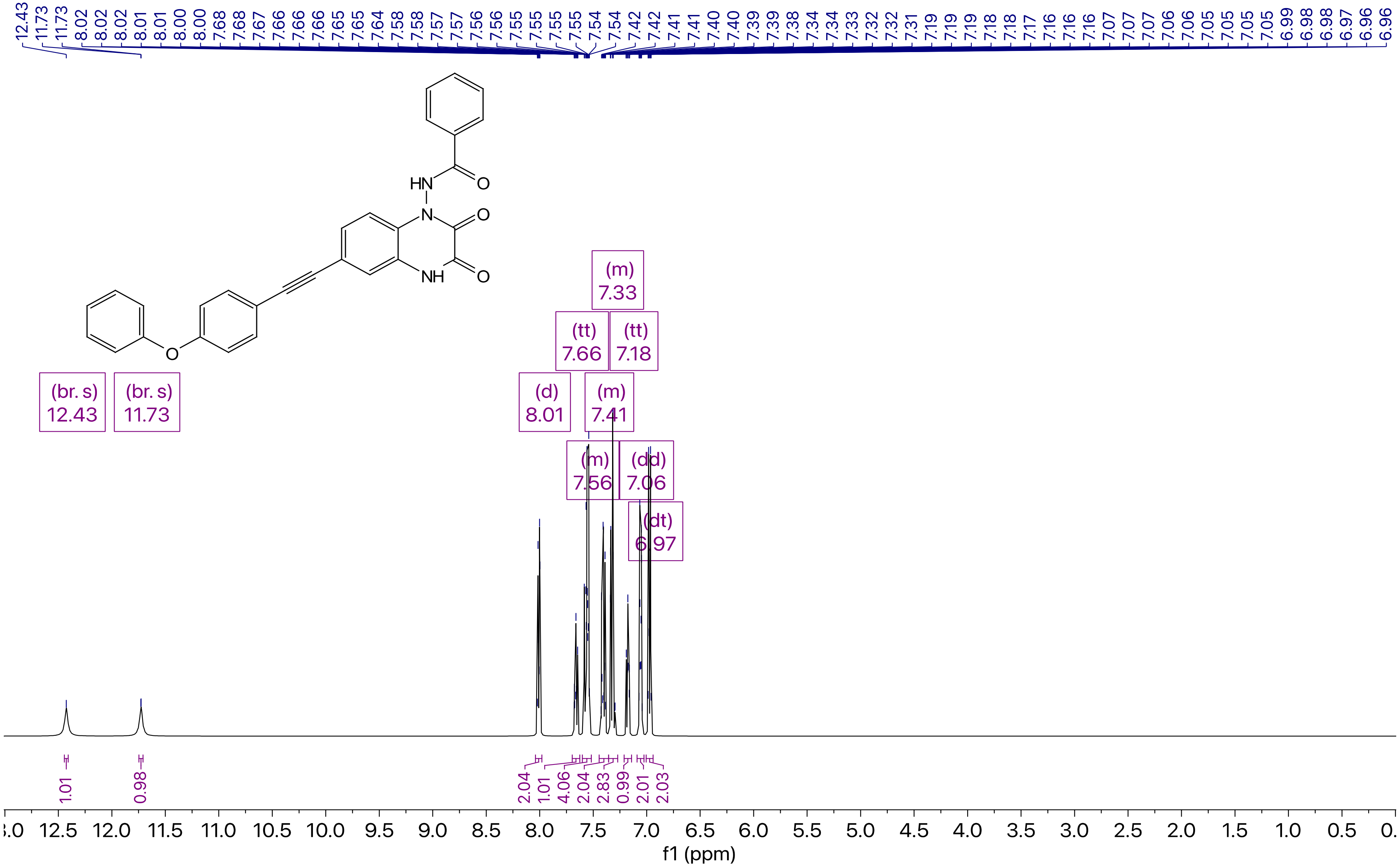

N-(2,3-dioxo-6-((4-phenoxyphenyl)ethynyl)-3,4-dihydroquinoxalin-1(2H)-yl)benzamide (**13**)

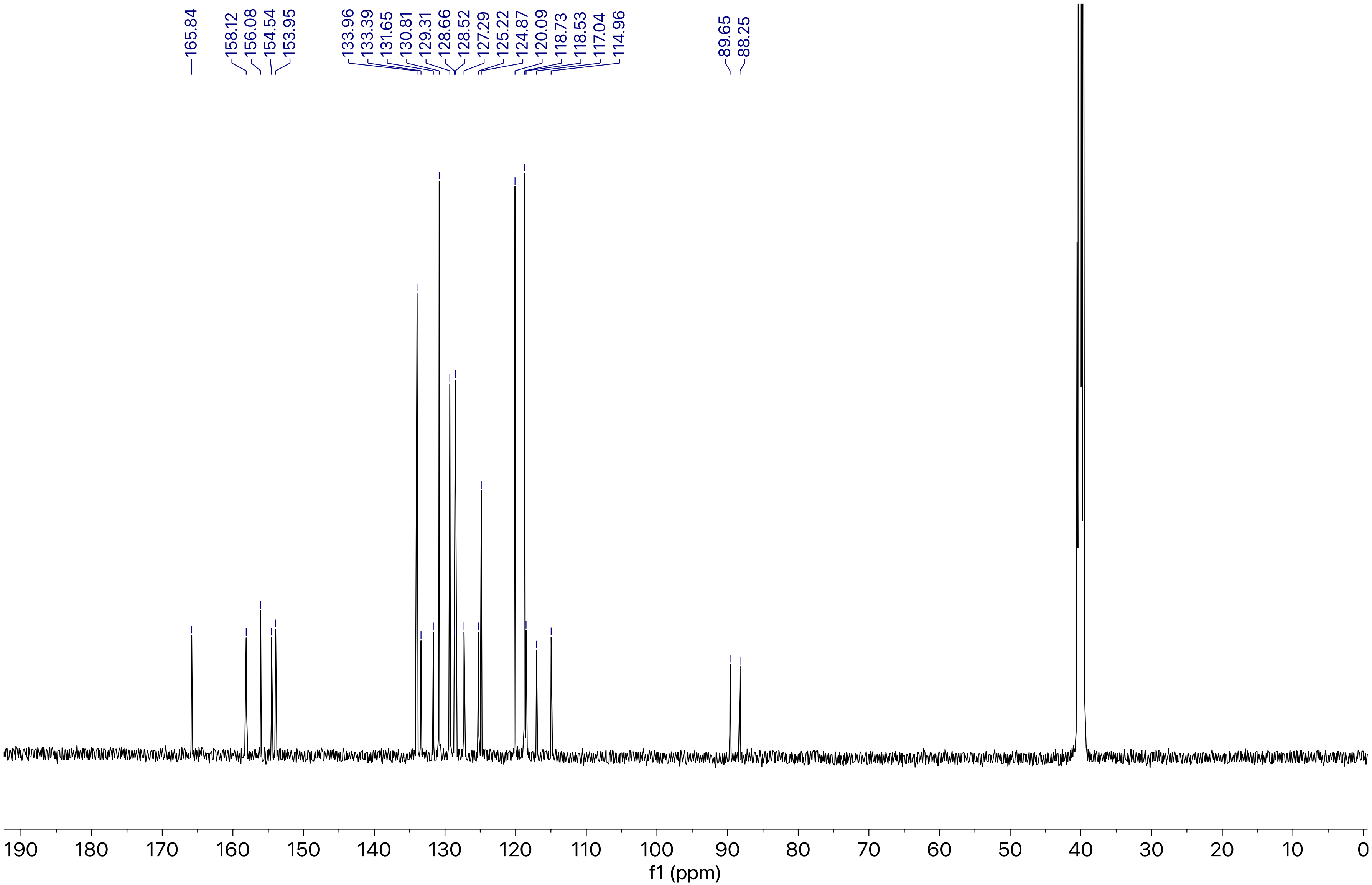

3-((1-benzamido-2,3-dioxo-1,2,3,4-tetrahydroquinoxalin-6-yl)ethynyl)benzoic acid (**14**)

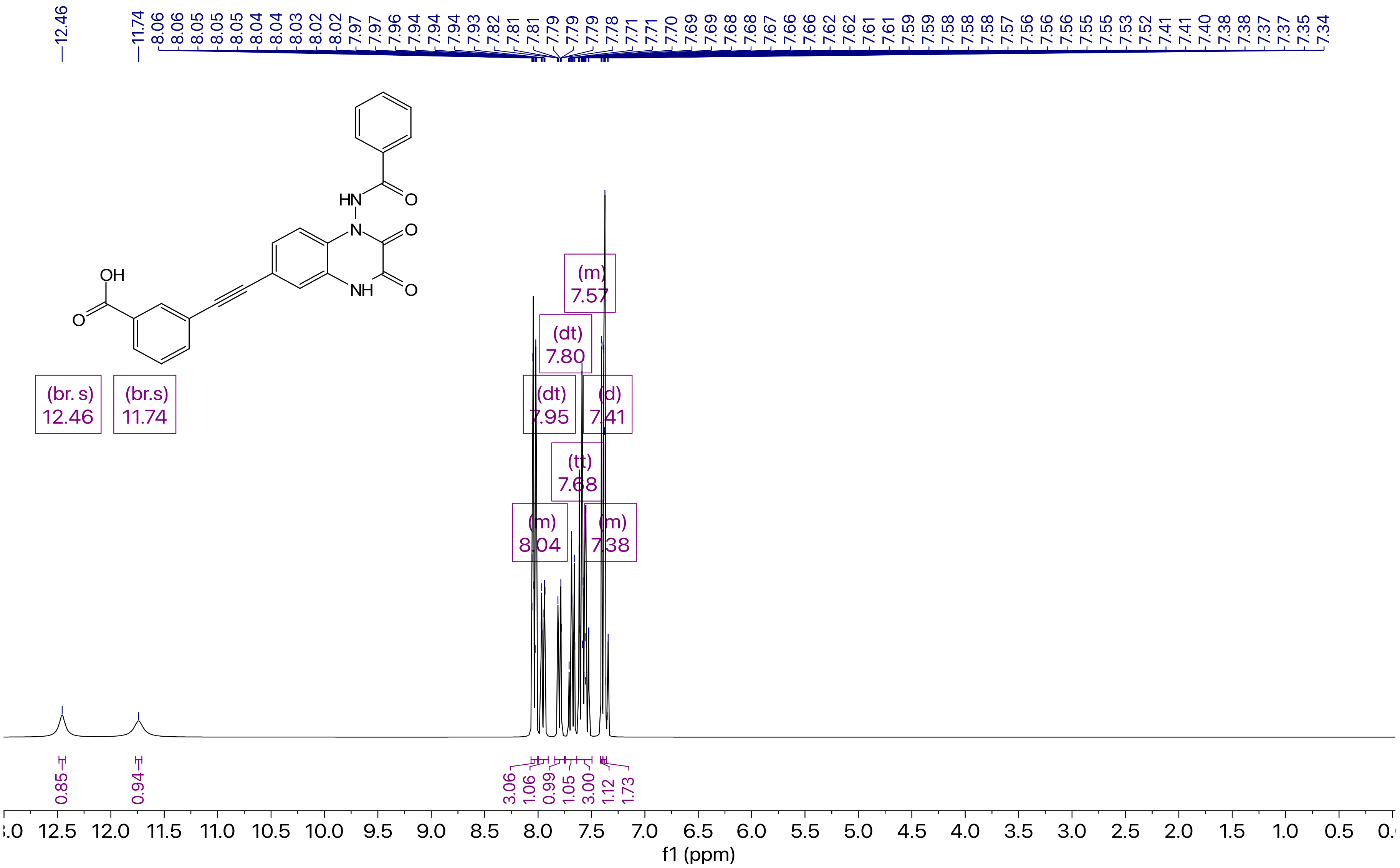

3-((1-benzamido-2,3-dioxo-1,2,3,4-tetrahydroquinoxalin-6-yl)ethynyl)benzoic acid (**14**)

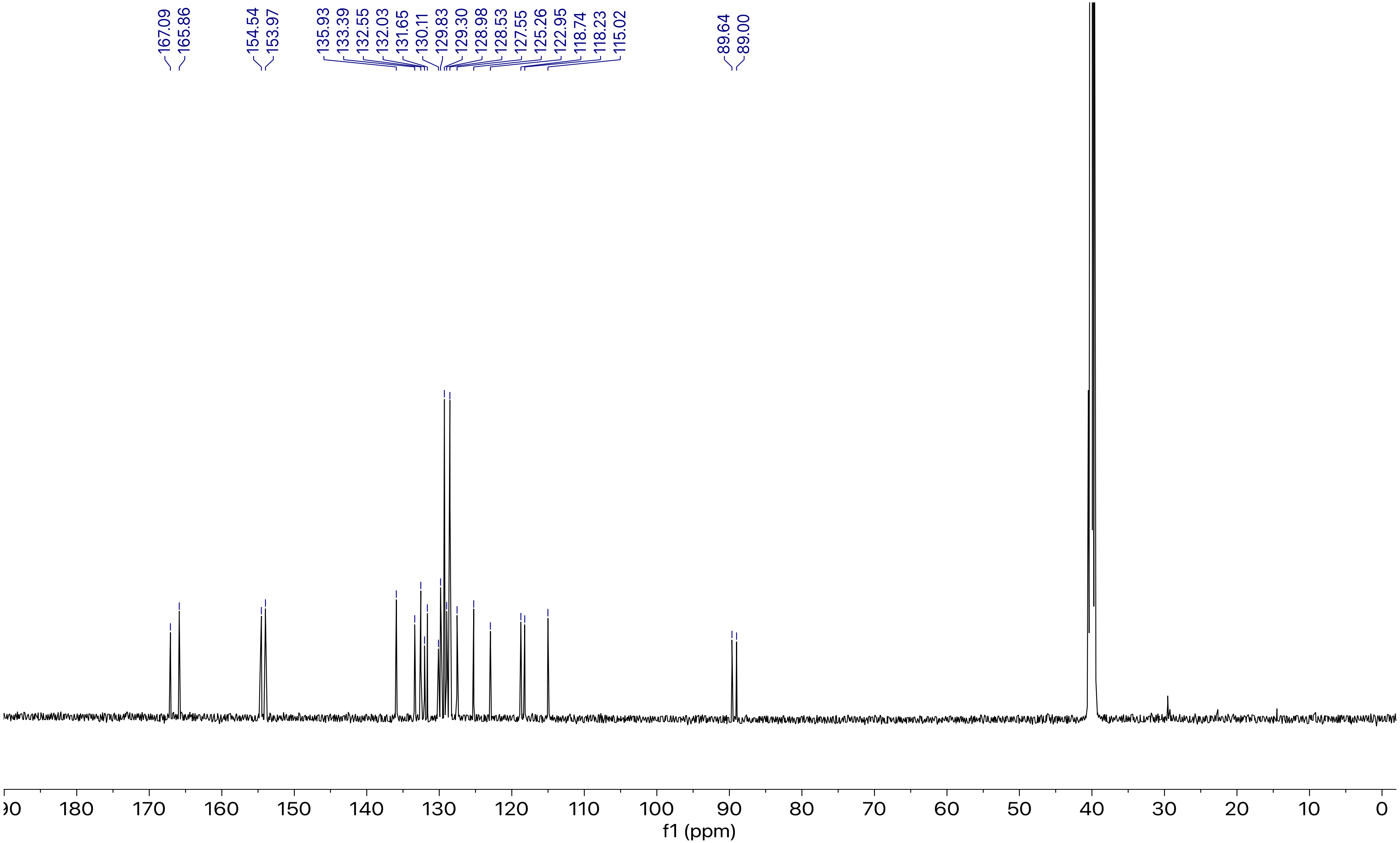

4-((1-benzamido-2,3-dioxo-1,2,3,4-tetrahydroquinoxalin-6-yl)ethynyl)benzoic acid (**15**)

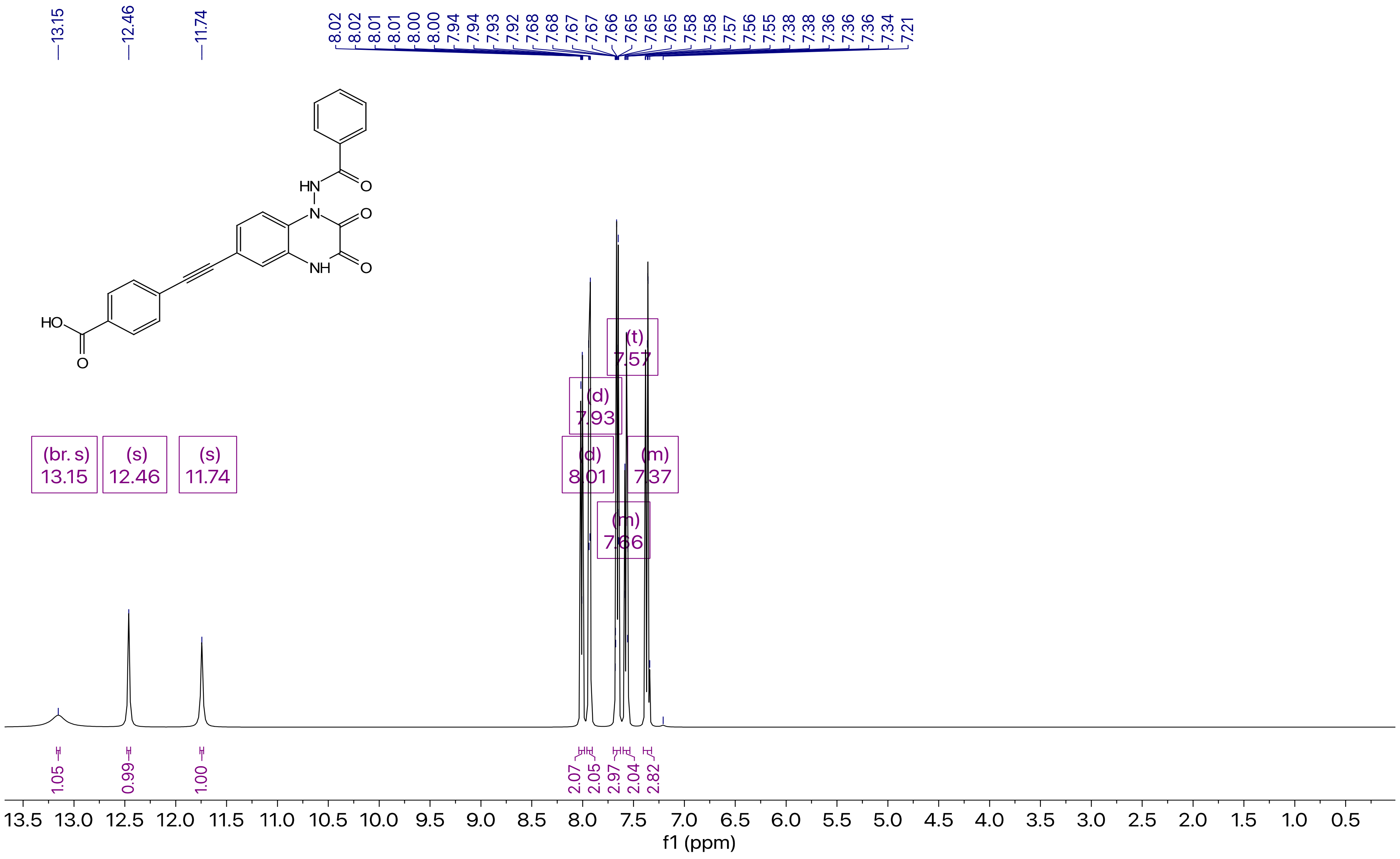

4-((1-benzamido-2,3-dioxo-1,2,3,4-tetrahydroquinoxalin-6-yl)ethynyl)benzoic acid (**15**)

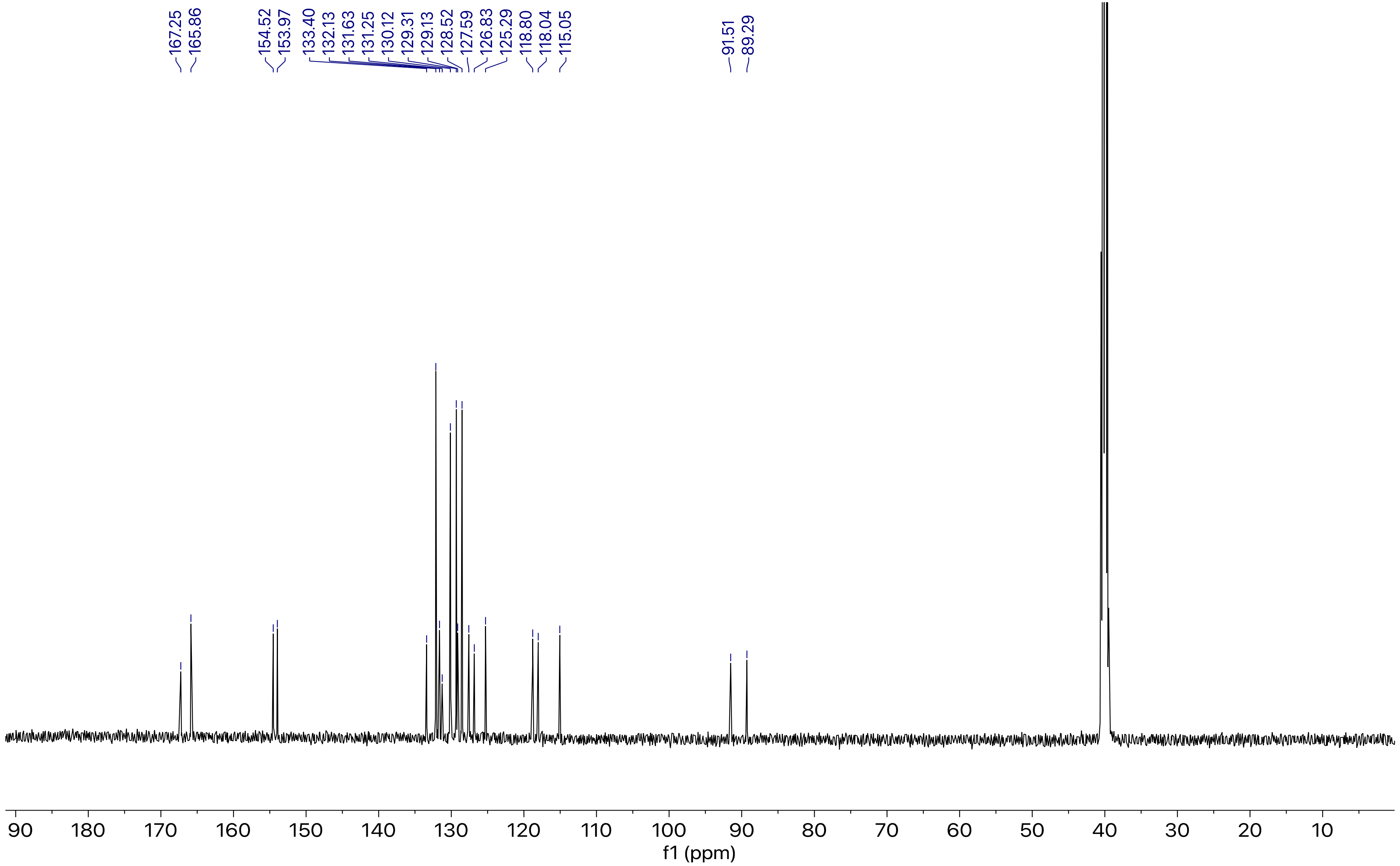

N-(2,3-dioxo-6-((4-sulfamoylphenyl)ethynyl)-3,4-dihydroquinoxalin-1(2*H*)-yl)benzamide (**16**)

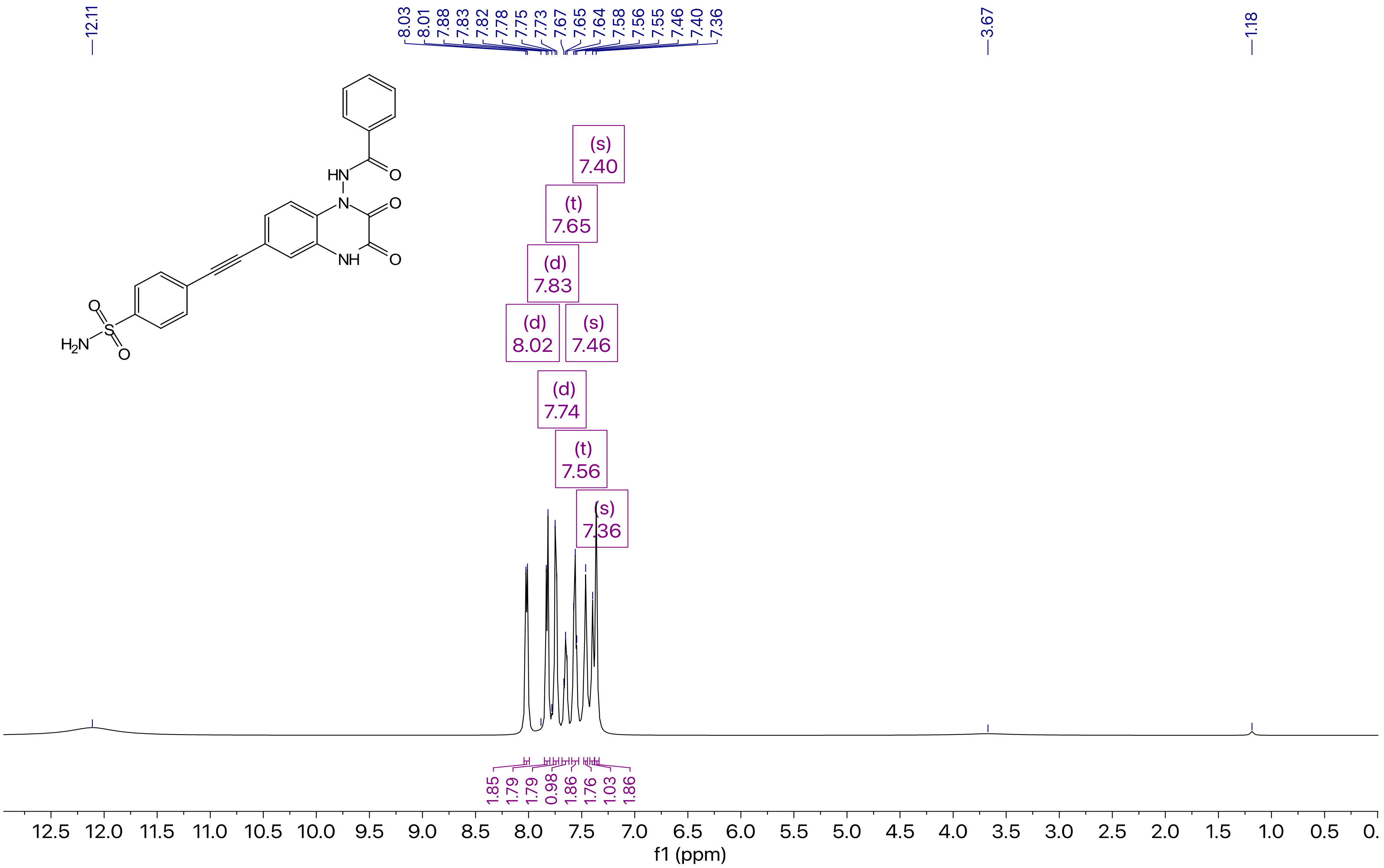

N-(2,3-dioxo-6-((4-sulfamoylphenyl)ethynyl)-3,4-dihydroquinoxalin-1(2*H*)-yl)benzamide (**16**)

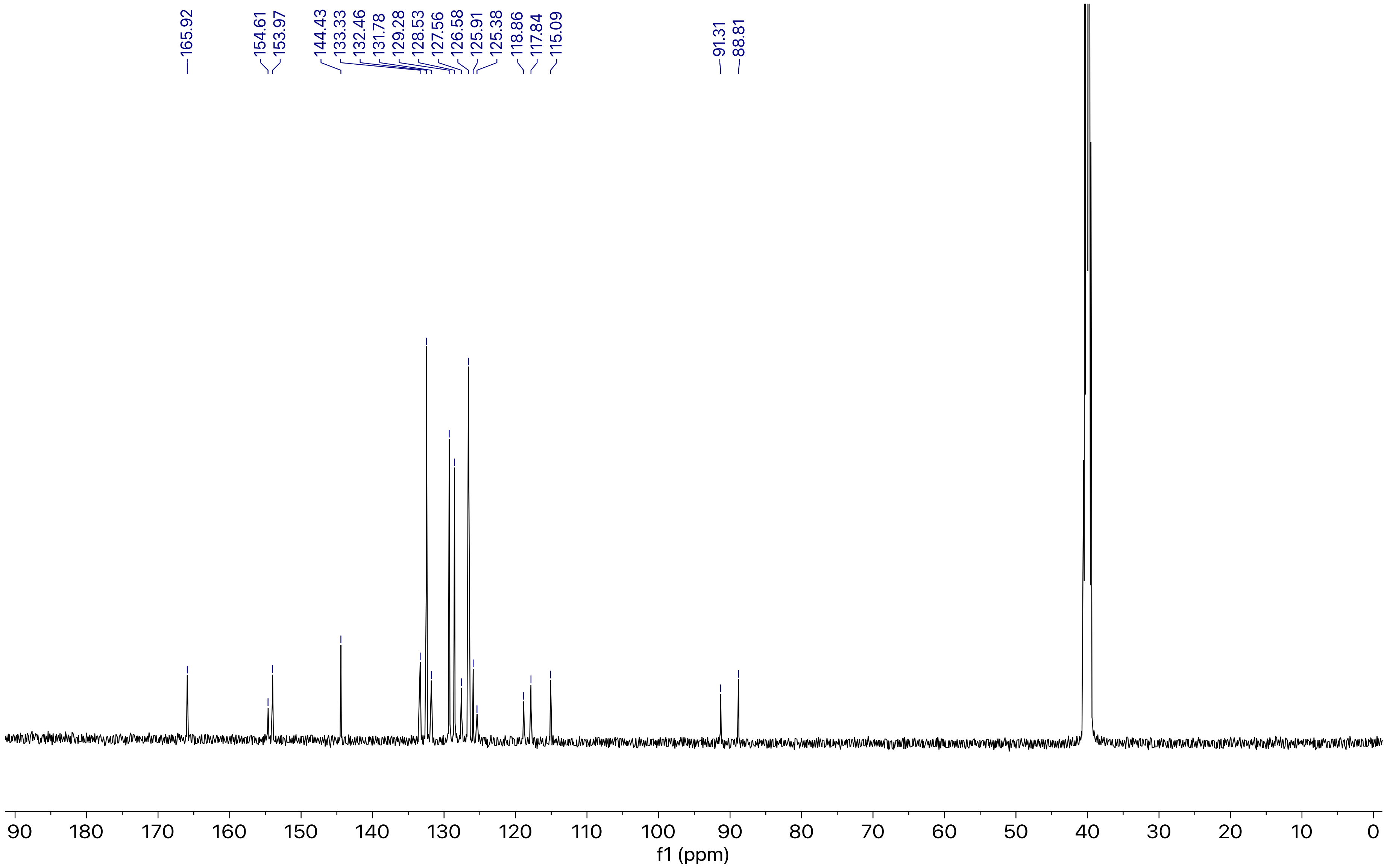

4-((1-benzamido-2,3-dioxo-1,2,3,4-tetrahydroquinoxalin-6-yl)ethynyl)benzenesulfonyl fluoride (**17**)

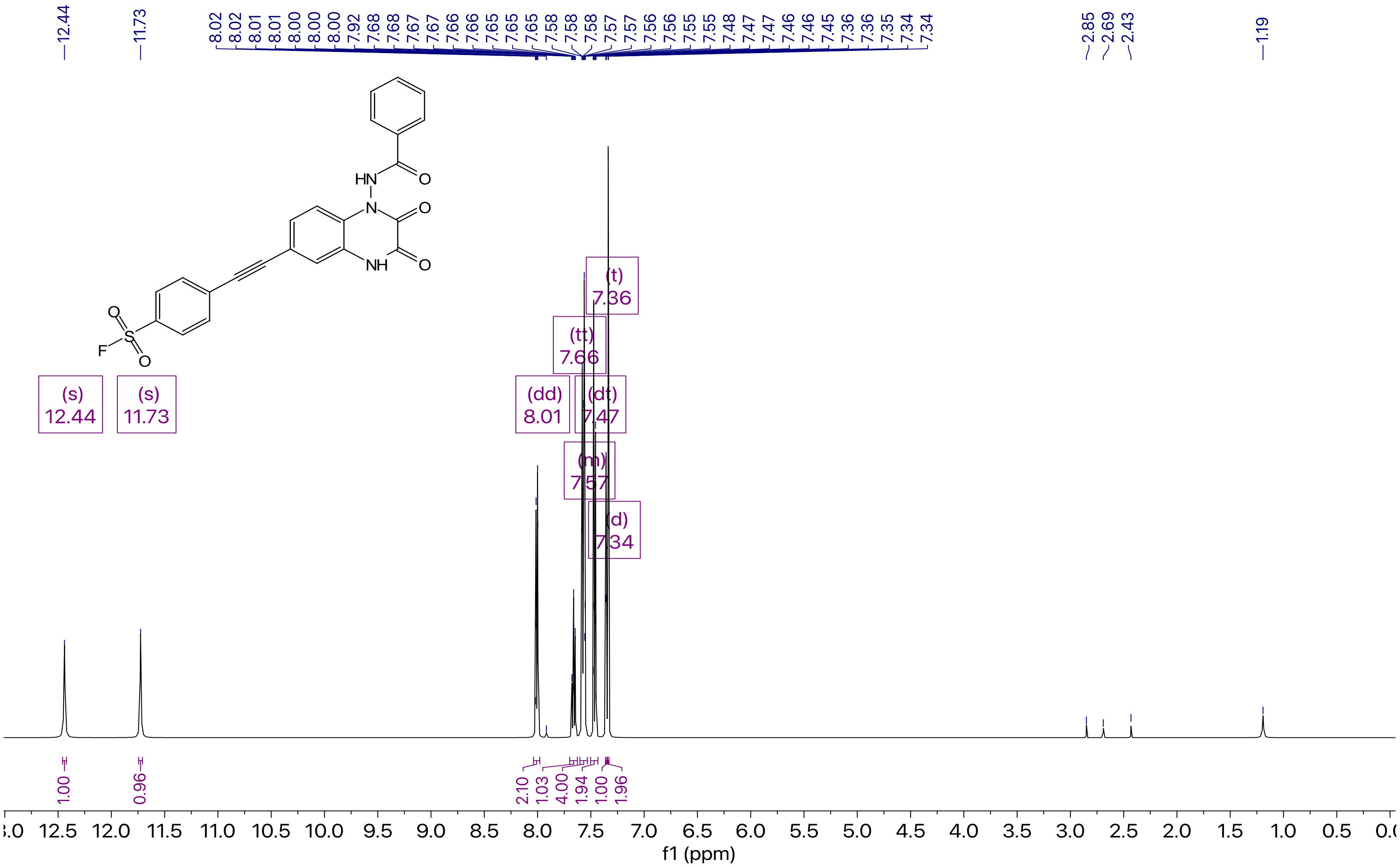

4-((1-benzamido-2,3-dioxo-1,2,3,4-tetrahydroquinoxalin-6-yl)ethynyl)benzenesulfonyl fluoride (**17**)

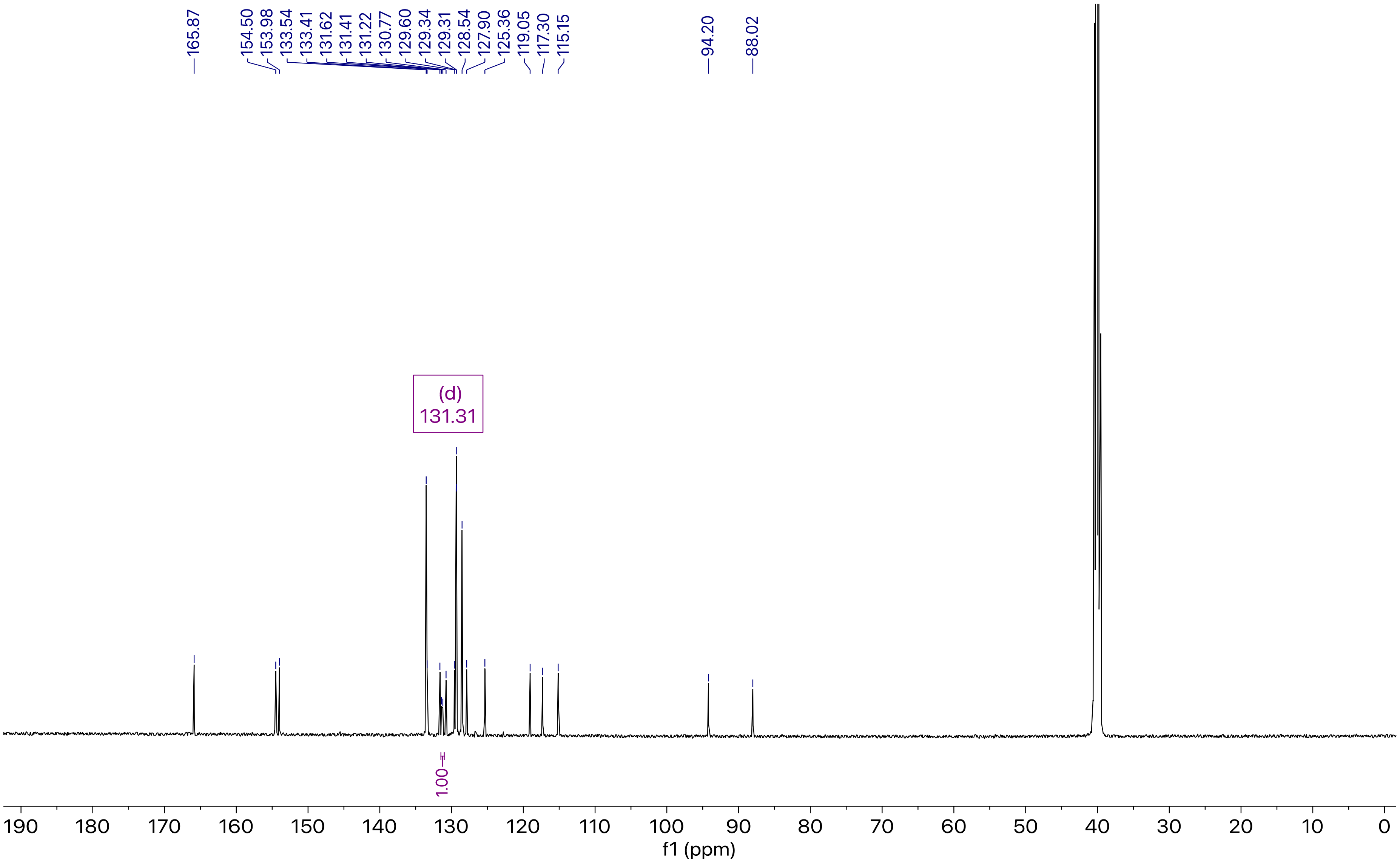

N-(6-((2-hydroxyphenyl)ethynyl)-2,3-dioxo-3,4-dihydroquinoxalin-1(2H)-yl)benzamide (**18**)

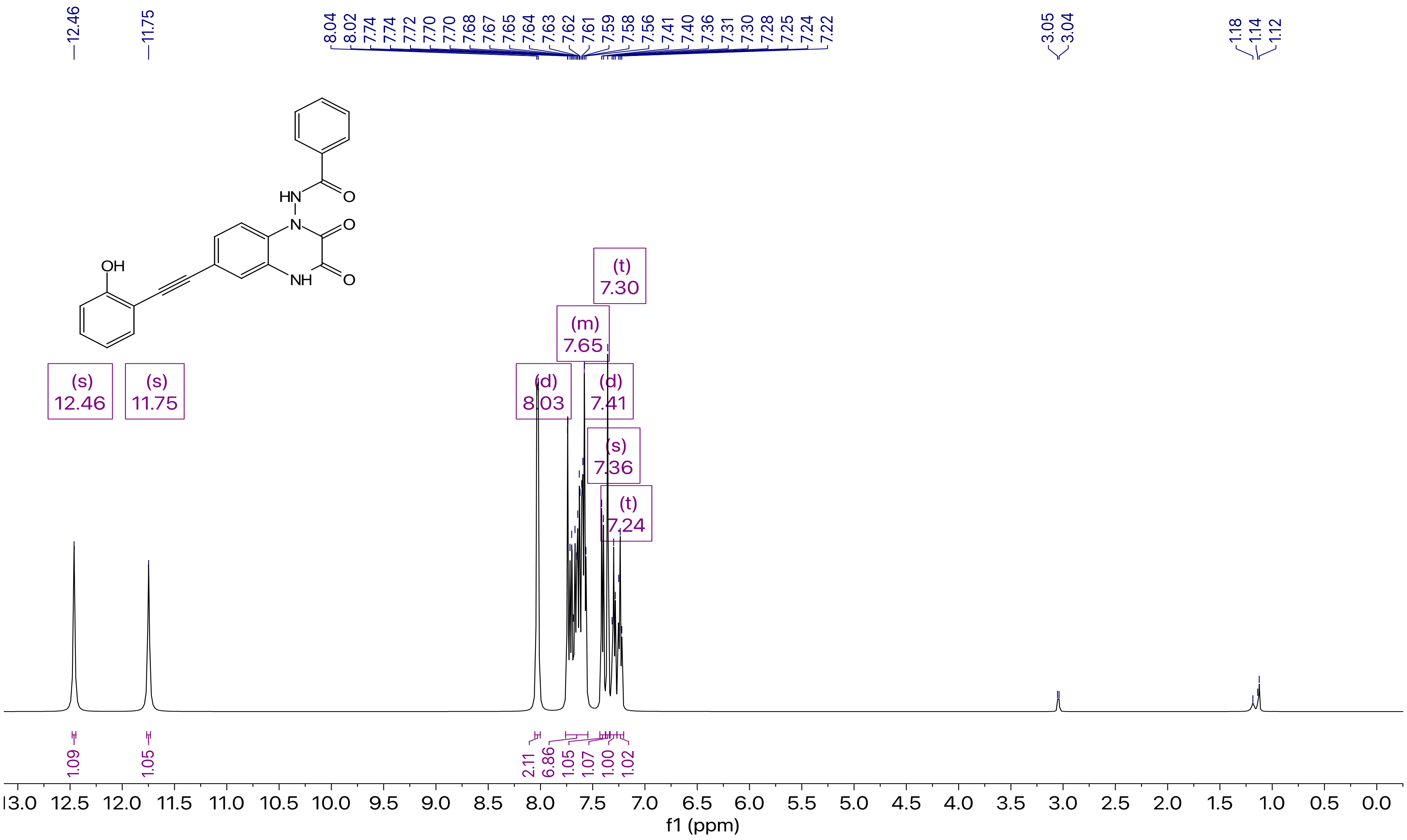

N-(6-((2-hydroxyphenyl)ethynyl)-2,3-dioxo-3,4-dihydroquinoxalin-1(2H)-yl)benzamide (**18**)

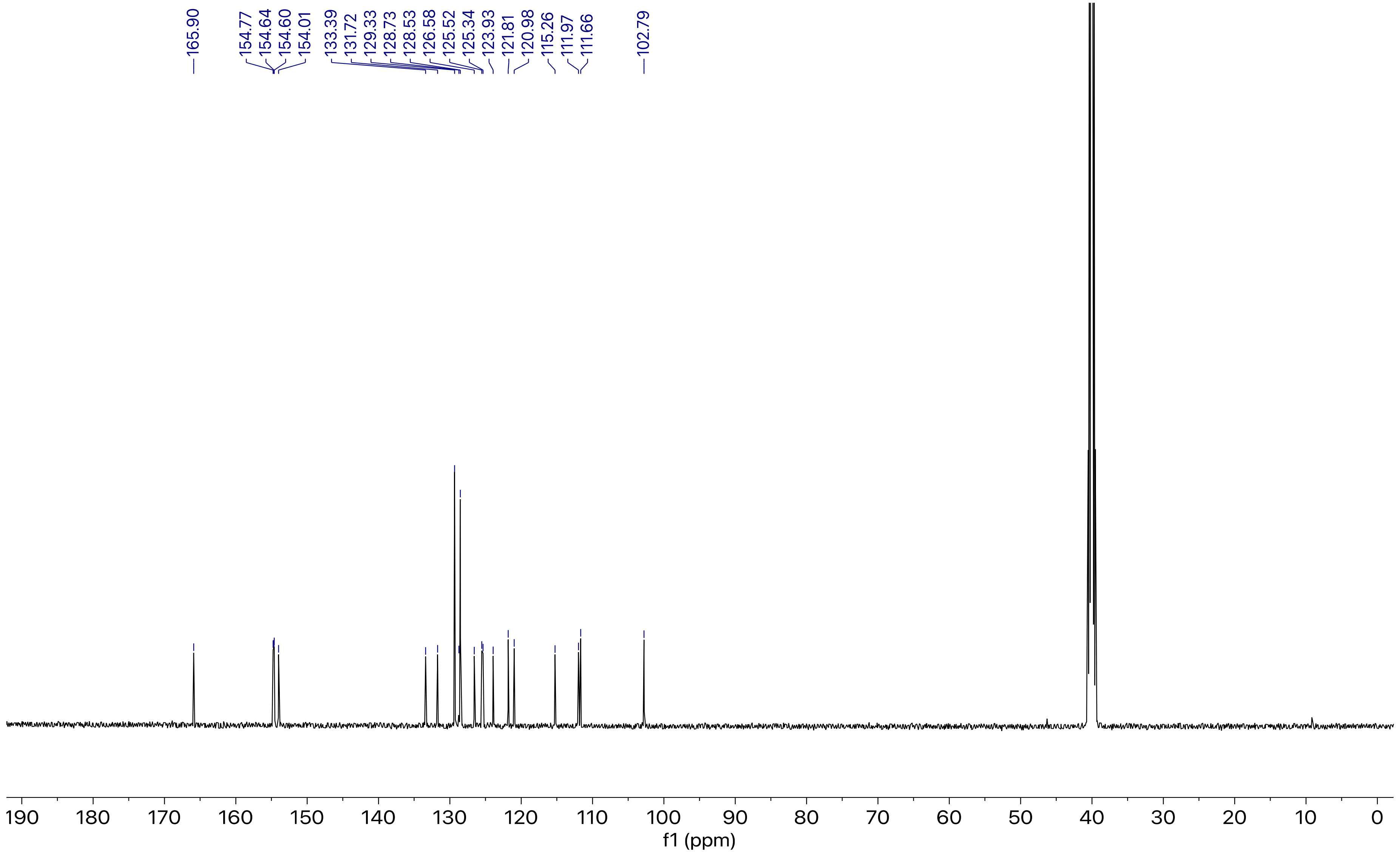

N-(6-((2-hydroxyphenyl)ethynyl)-2,3-dioxo-3,4-dihydroquinoxalin-1(2H)-yl)benzamide **(18)**

N-(6-((3-hydroxyphenyl)ethynyl)-2,3-dioxo-3,4-dihydroquinoxalin-1(2H)-yl)benzamide (**19**)

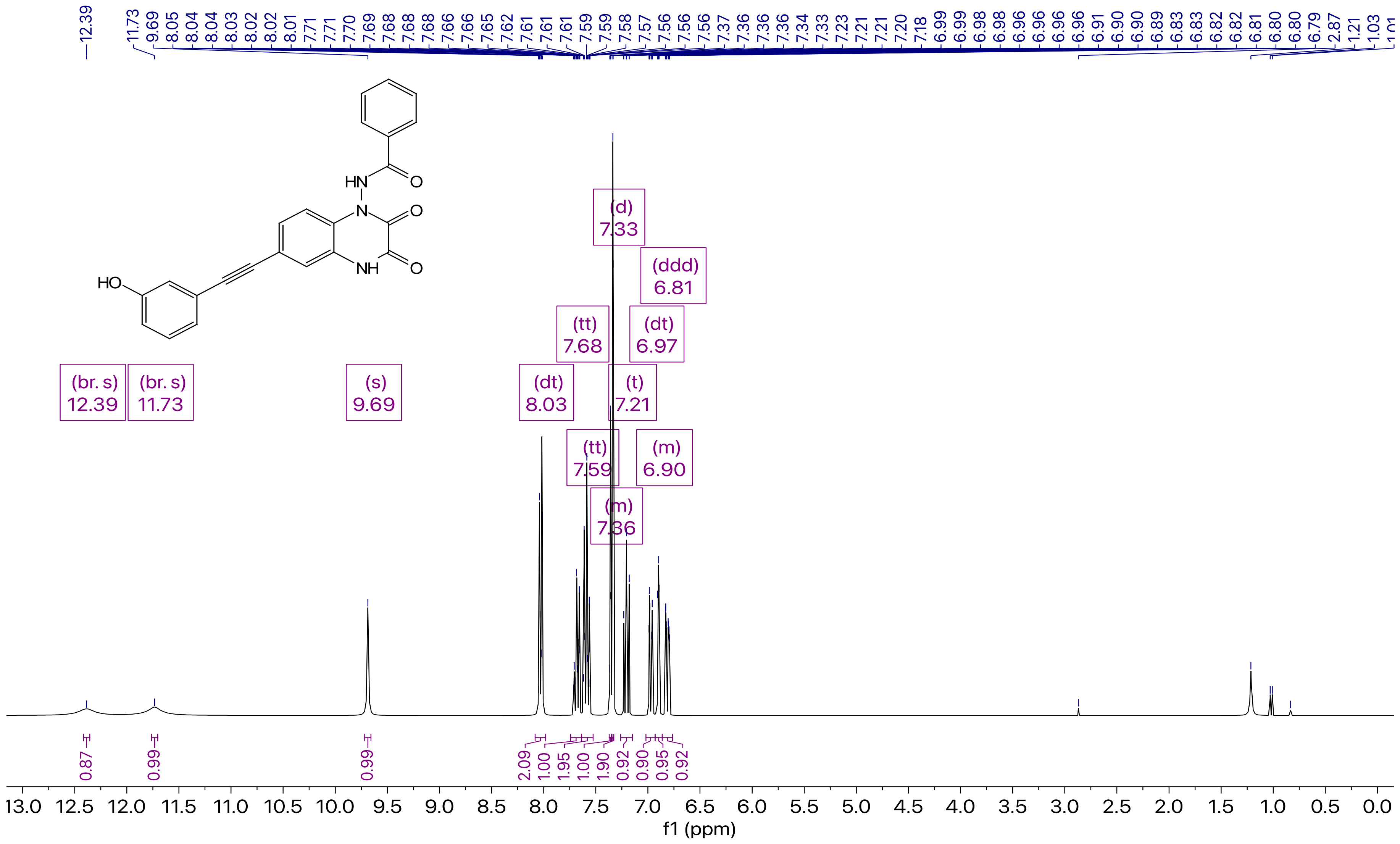

N-(6-((3-hydroxyphenyl)ethynyl)-2,3-dioxo-3,4-dihydroquinoxalin-1(2H)-yl)benzamide (**19**)

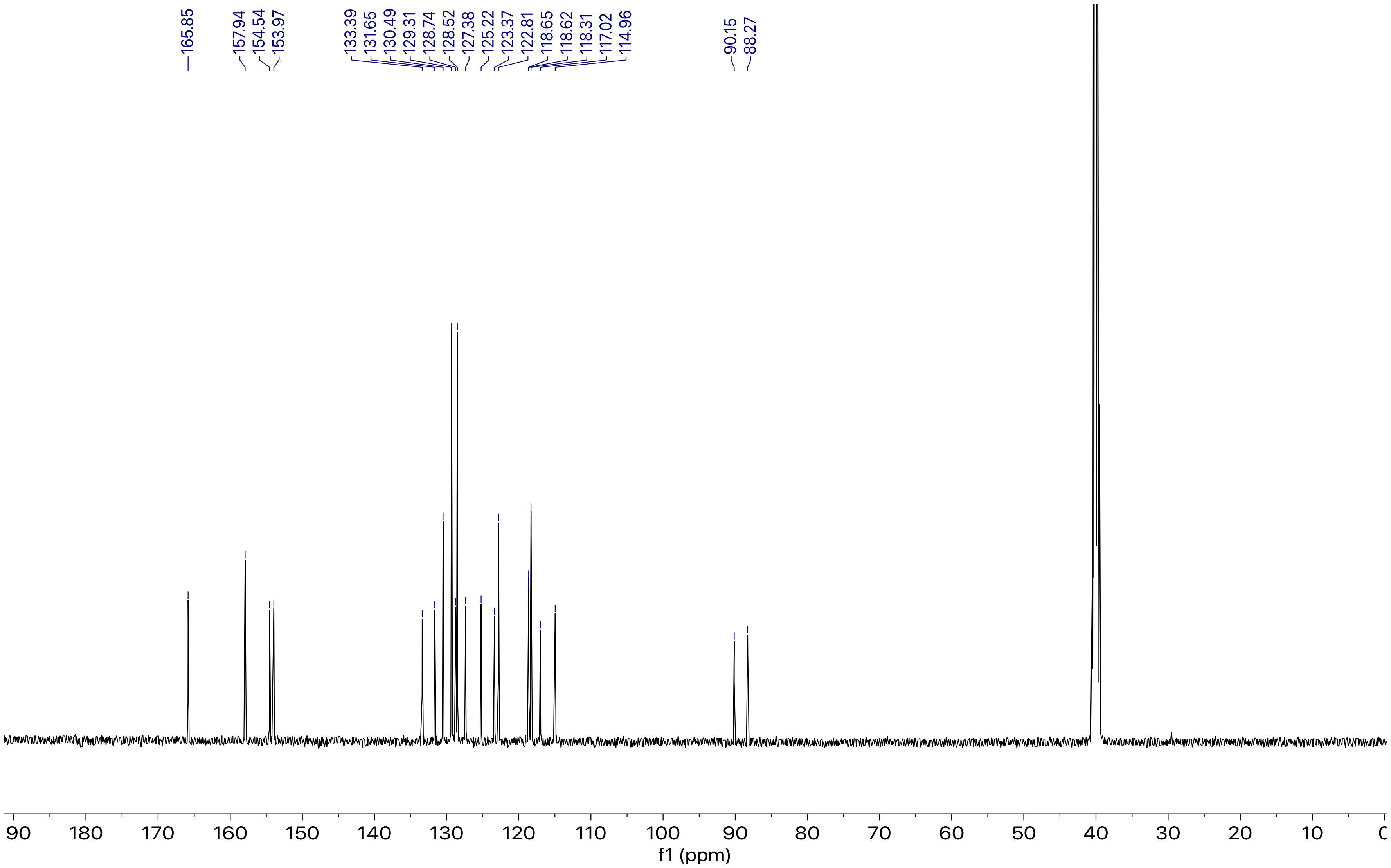

N-(6-((4-(hydroxymethyl)phenyl)ethynyl)-2,3-dioxo-3,4-dihydroquinoxalin-1(2*H*)-yl)benzamide (**20**)

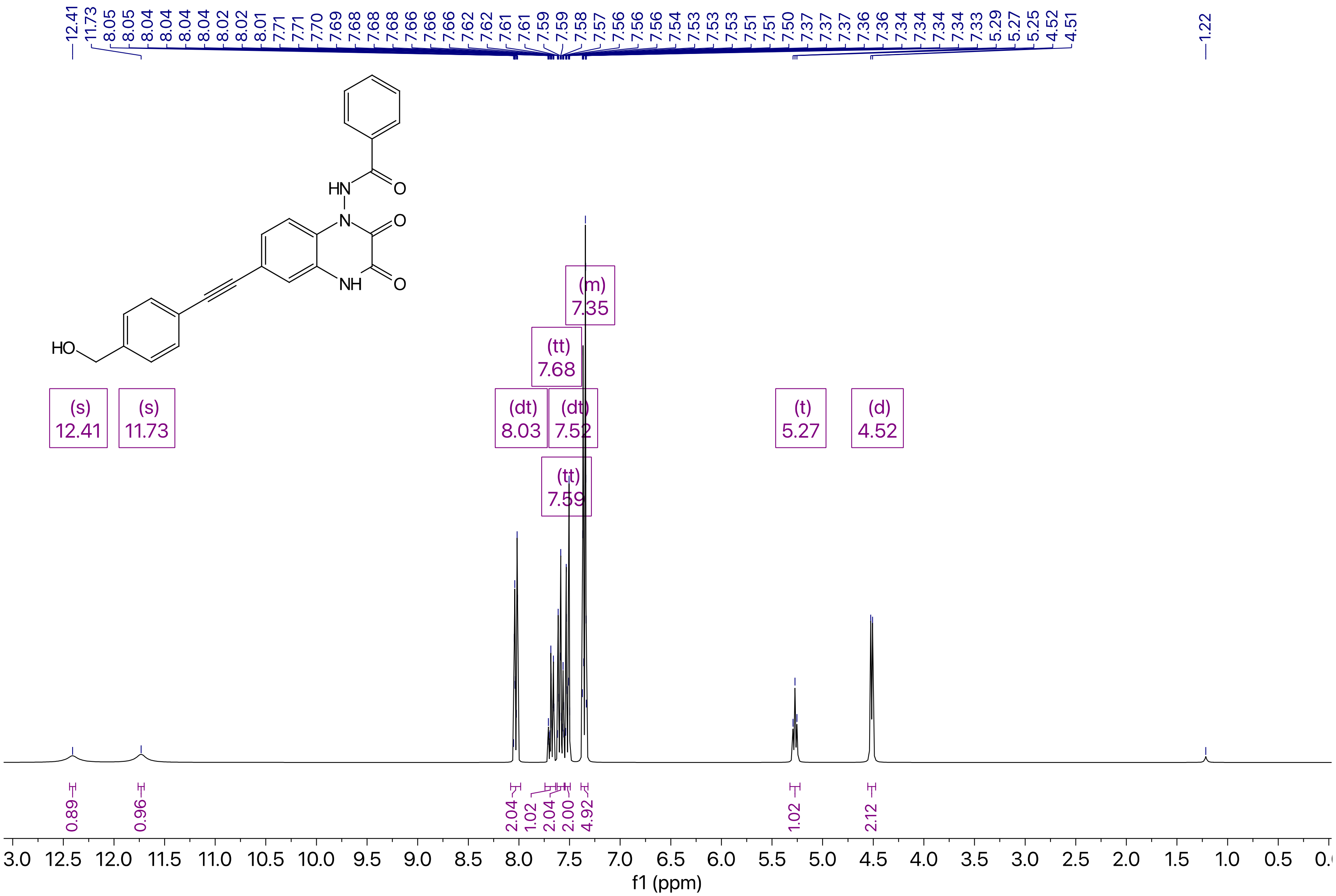

N-(6-((4-(hydroxymethyl)phenyl)ethynyl)-2,3-dioxo-3,4-dihydroquinoxalin-1(2*H*)-yl)benzamide (**20**)

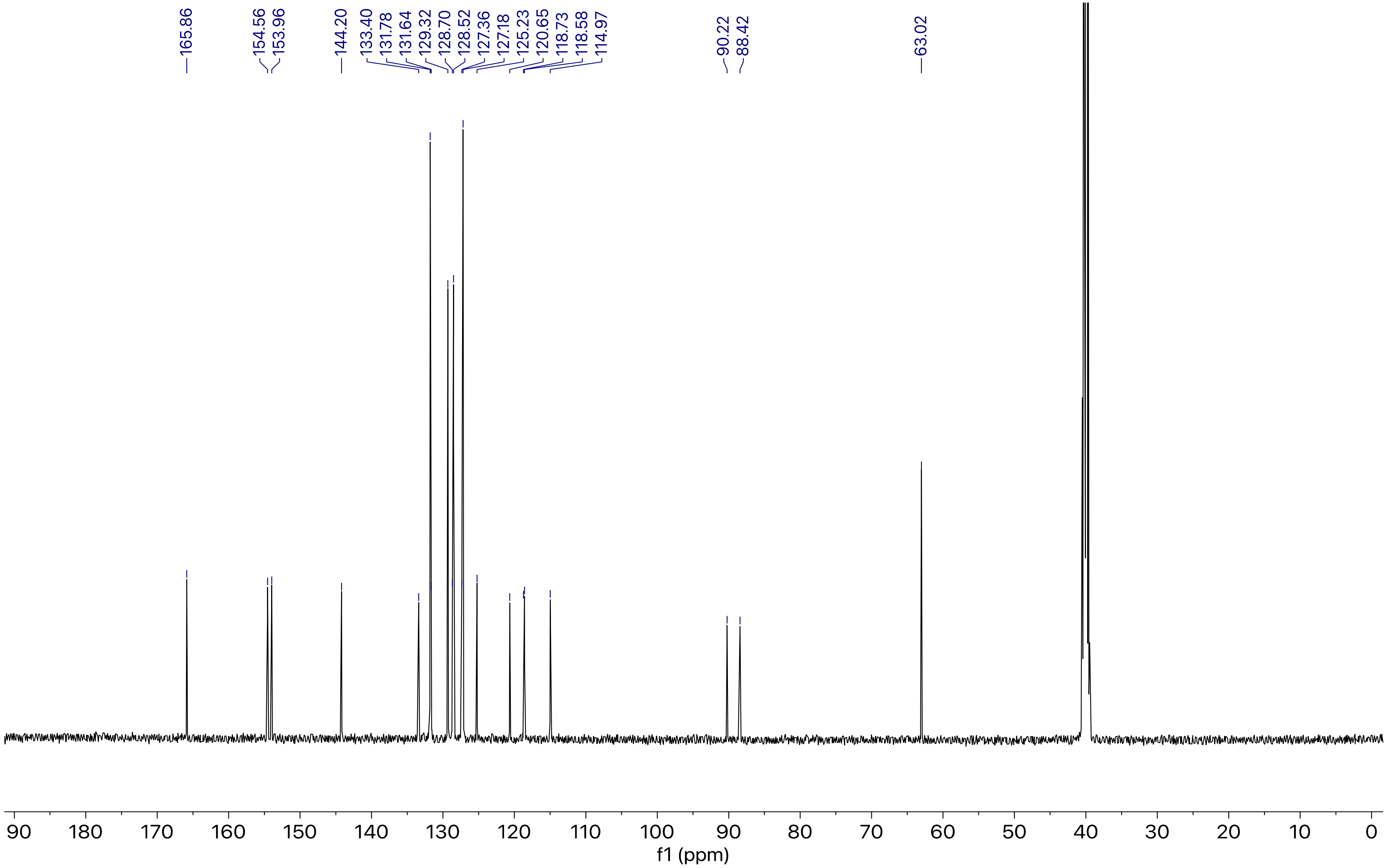

N-(6-((2-aminophenyl)ethynyl)-2,3-dioxo-3,4-dihydroquinoxalin-1(2H)-yl)benzamide (**21**)

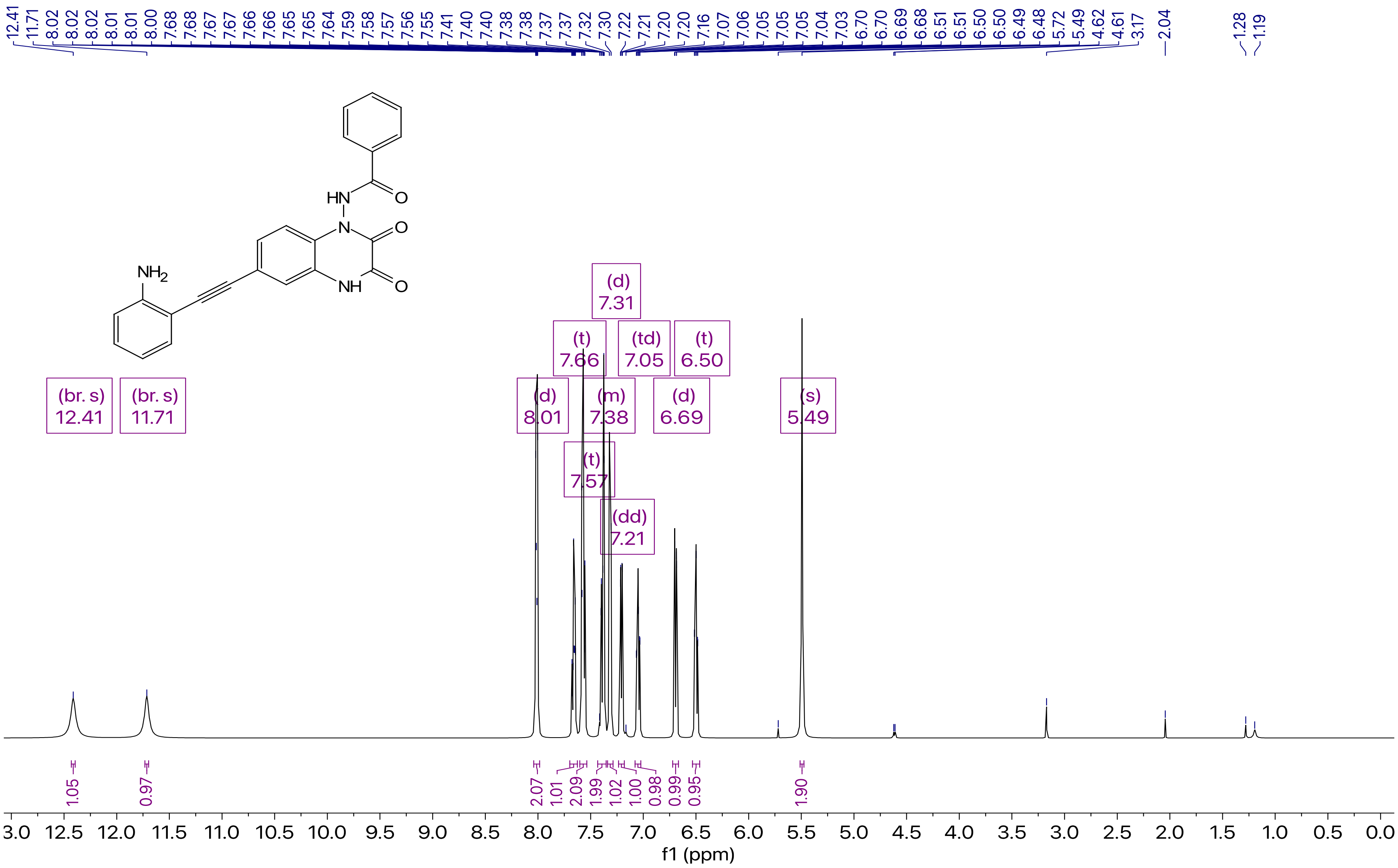

N-(6-((2-aminophenyl)ethynyl)-2,3-dioxo-3,4-dihydroquinoxalin-1(2H)-yl)benzamide (**21**)

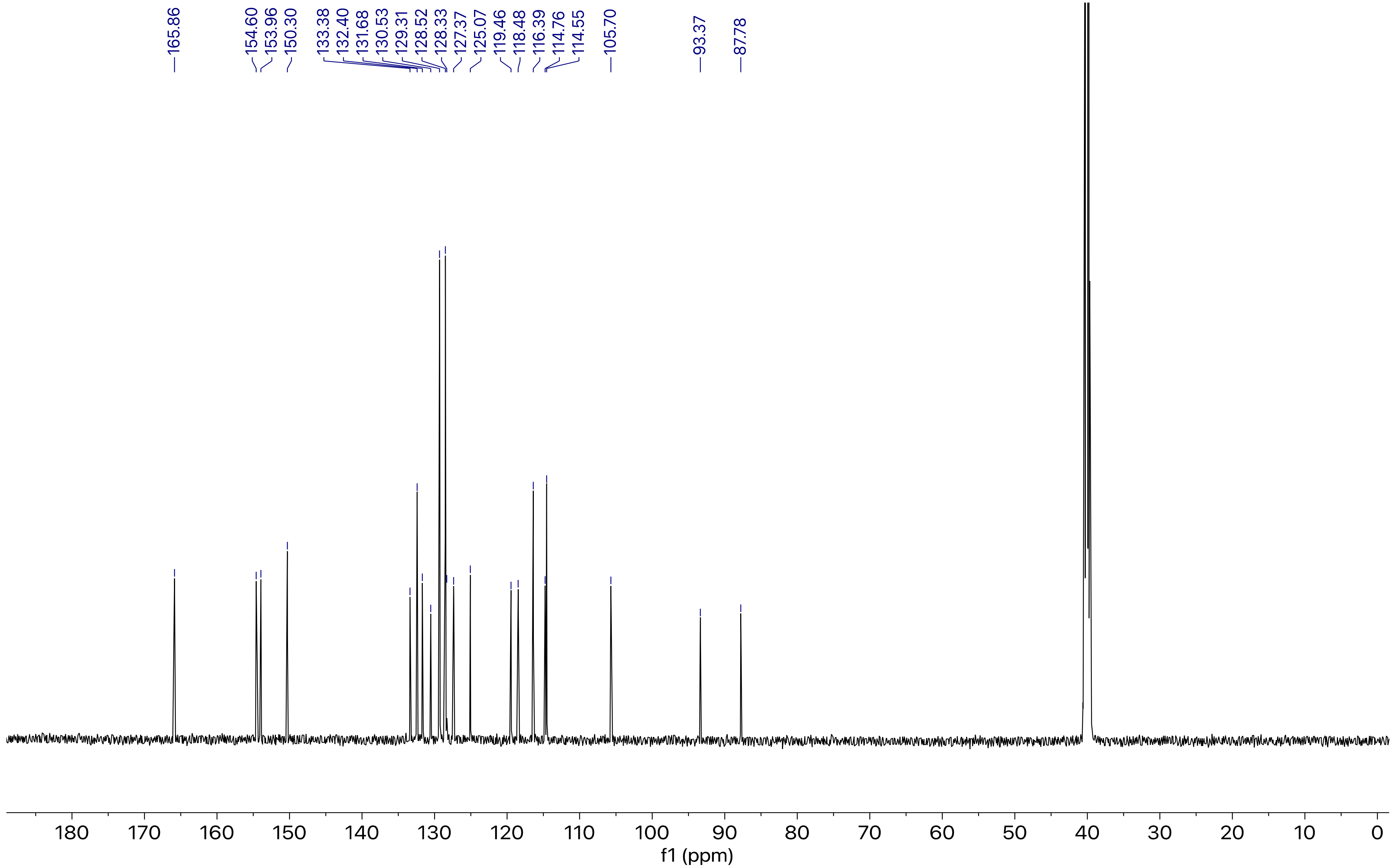

N-(6-((3-aminophenyl)ethynyl)-2,3-dioxo-3,4-dihydroquinoxalin-1(2H)-yl)benzamide (**22**)

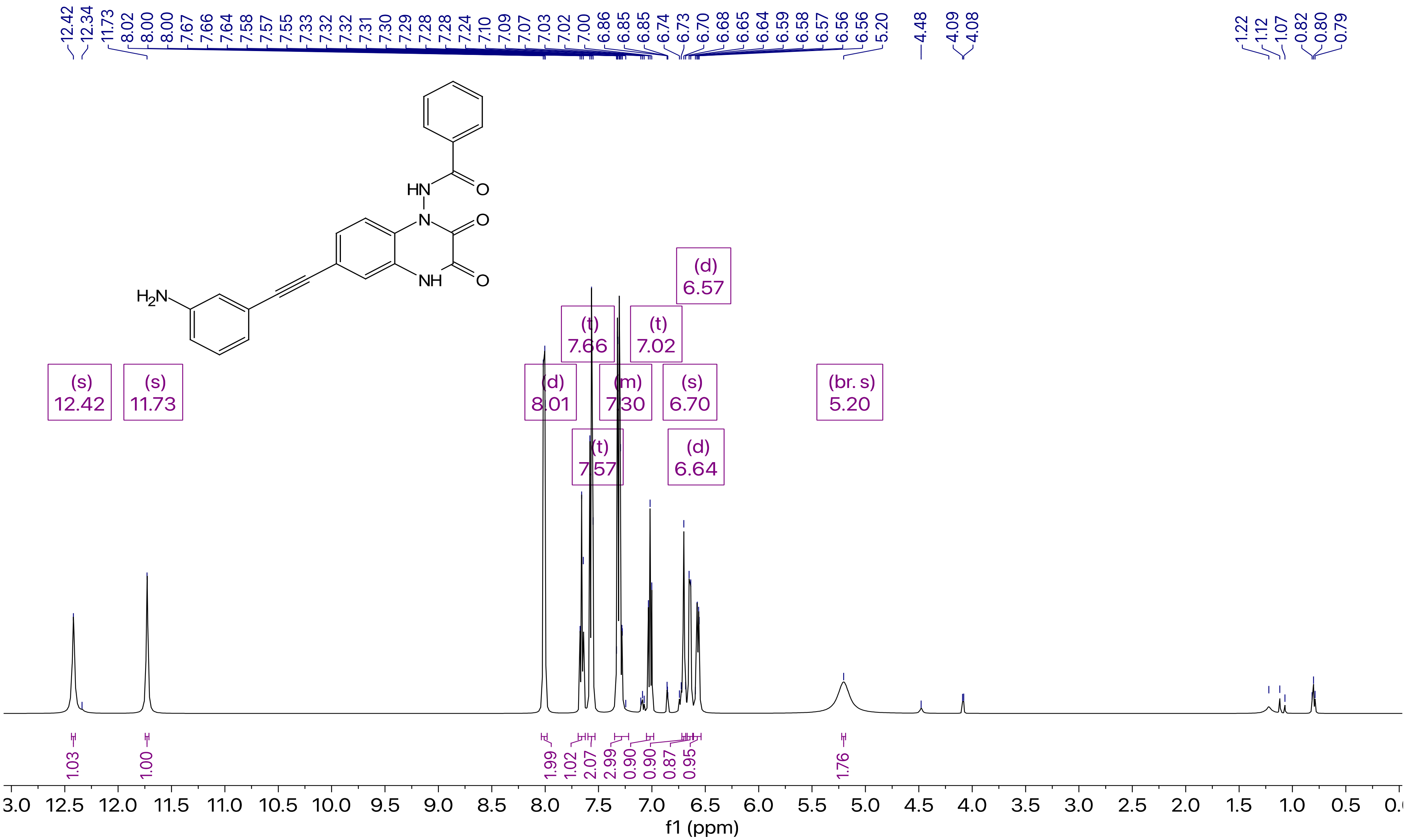

N-(6-((3-aminophenyl)ethynyl)-2,3-dioxo-3,4-dihydroquinoxalin-1(2H)-yl)benzamide (**22**)

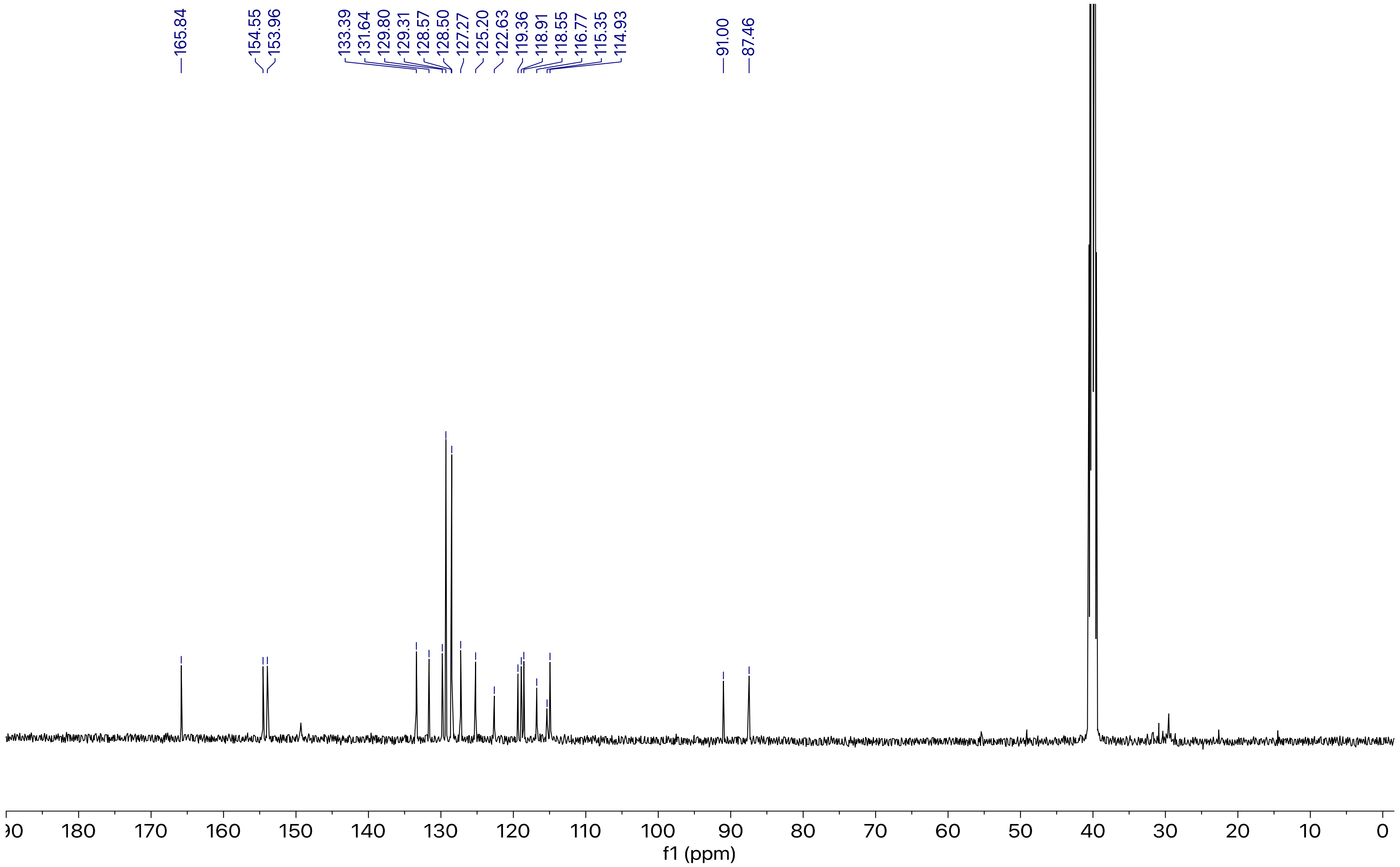

N-(6-((4-aminophenyl)ethynyl)-2,3-dioxo-3,4-dihydroquinoxalin-1(2H)-yl)benzamide (**23**)

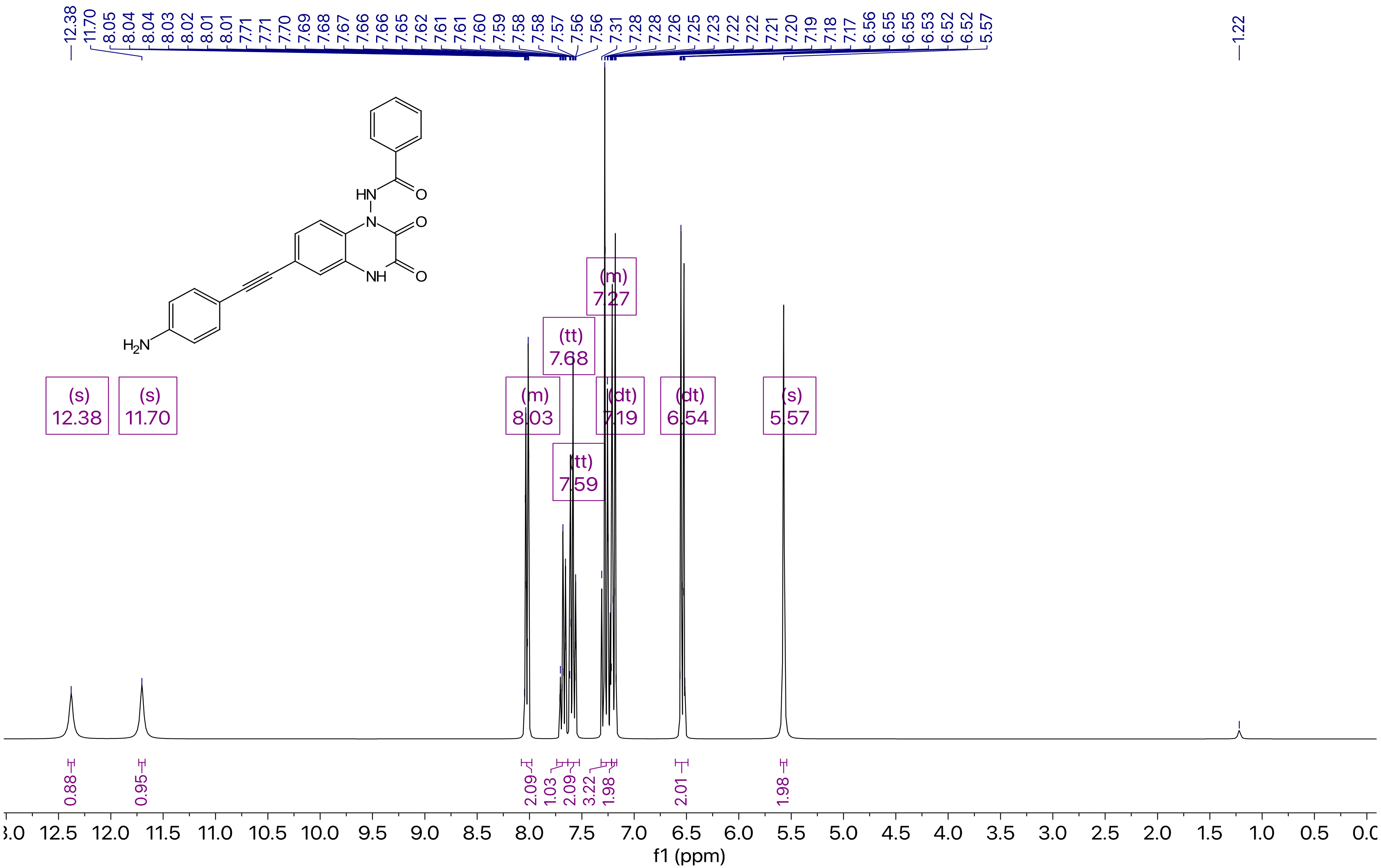

N-(6-((4-aminophenyl)ethynyl)-2,3-dioxo-3,4-dihydroquinoxalin-1(2H)-yl)benzamide (**23**)

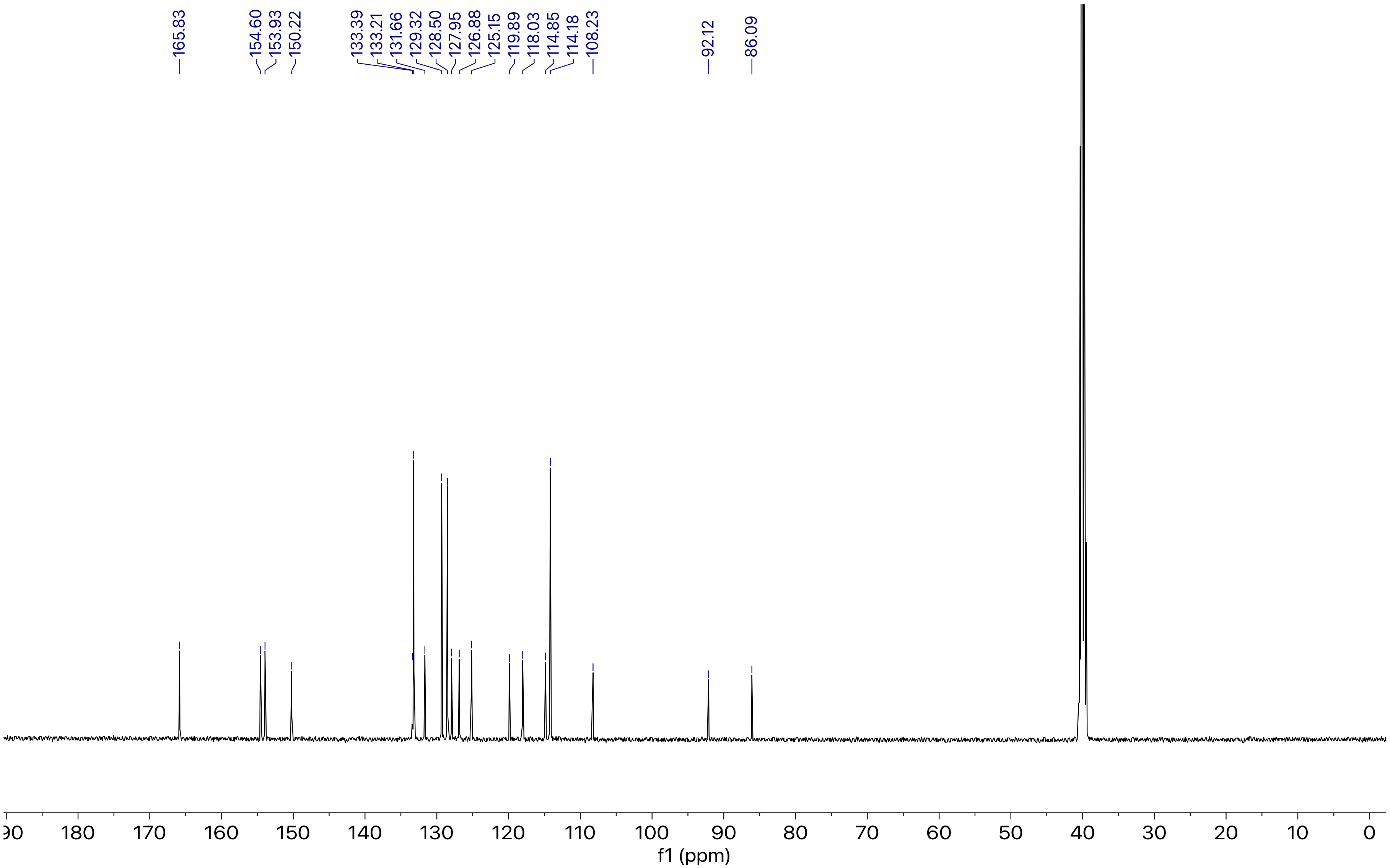

N-(6-((4-(dimethylamino)phenyl)ethynyl)-2,3-dioxo-3,4-dihydroquinoxalin-1(2H)-yl)benzamide (**24**)

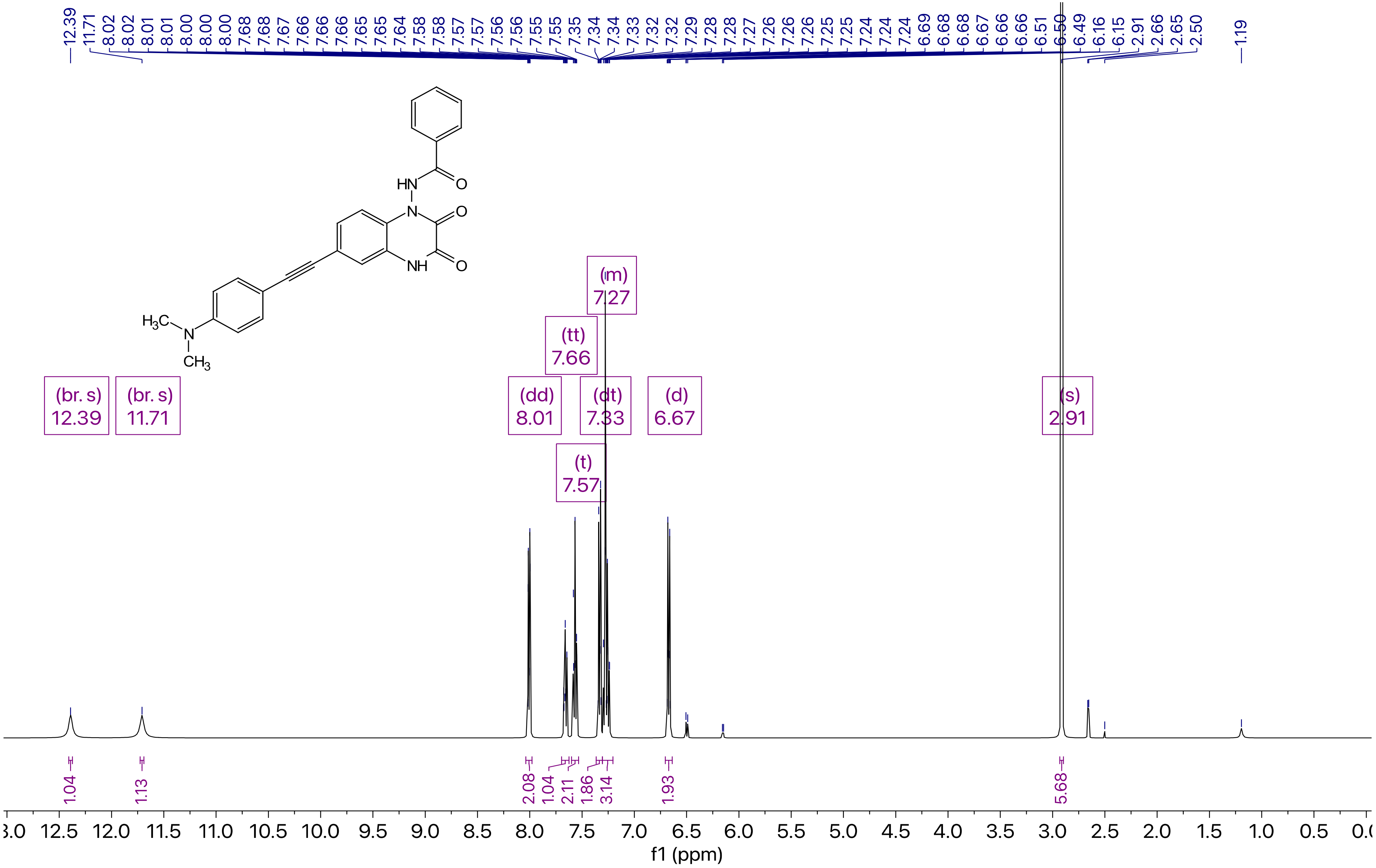

N-(6-((4-(dimethylamino)phenyl)ethynyl)-2,3-dioxo-3,4-dihydroquinoxalin-1(2*H*)-yl)benzamide (**24**)

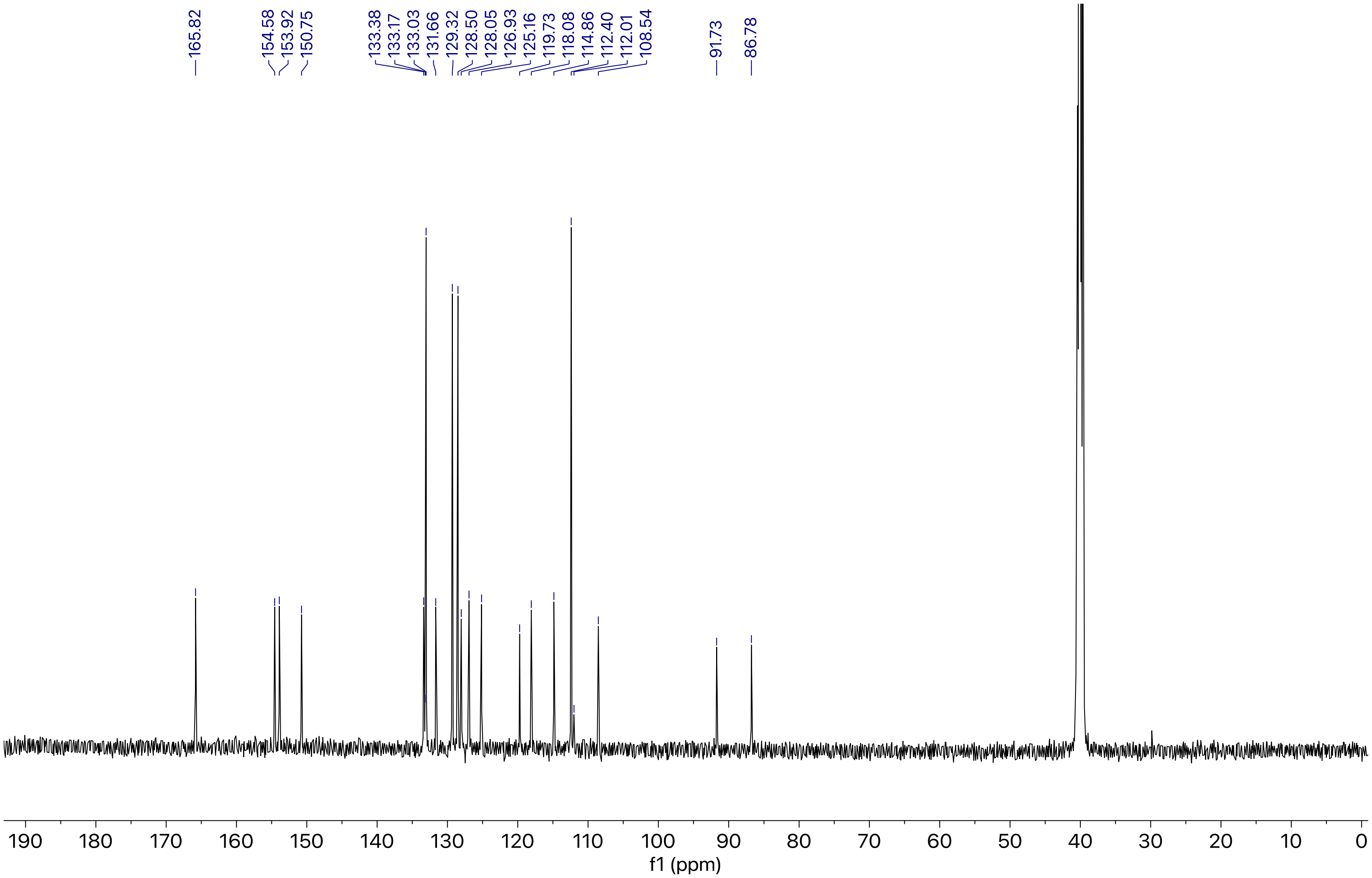

N-(2,3-dioxo-6-(pyridin-4-ylethynyl)-3,4-dihydroquinoxalin-1(2H)-yl)benzamide (**25**)

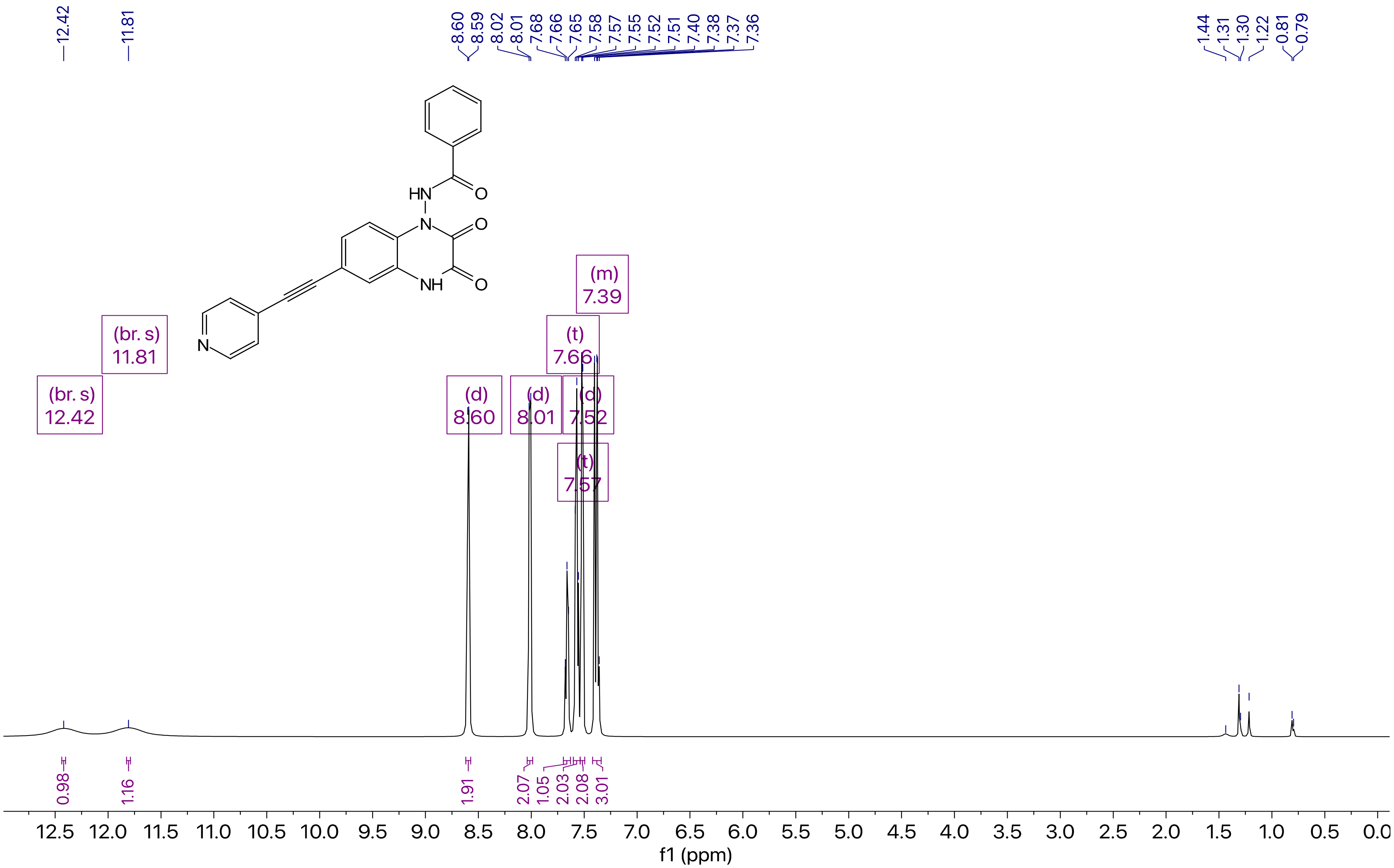

N-(2,3-dioxo-6-(pyridin-4-ylethynyl)-3,4-dihydroquinoxalin-1(2H)-yl)benzamide (**25**)

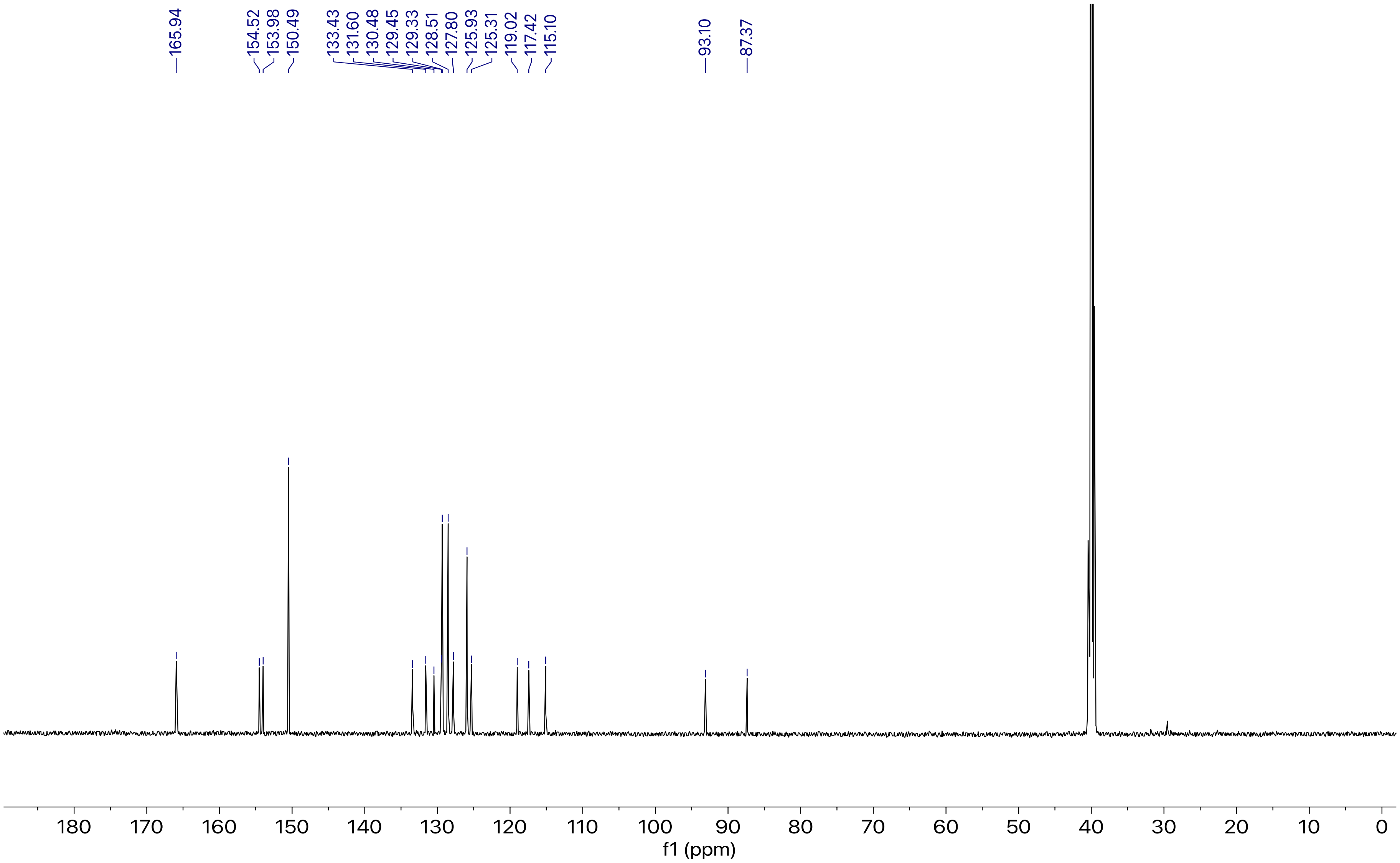

N-(6-((6-hydroxypyridin-3-yl)ethynyl)-2,3-dioxo-3,4-dihydroquinoxalin-1(2H)-yl)benzamide (**26**)

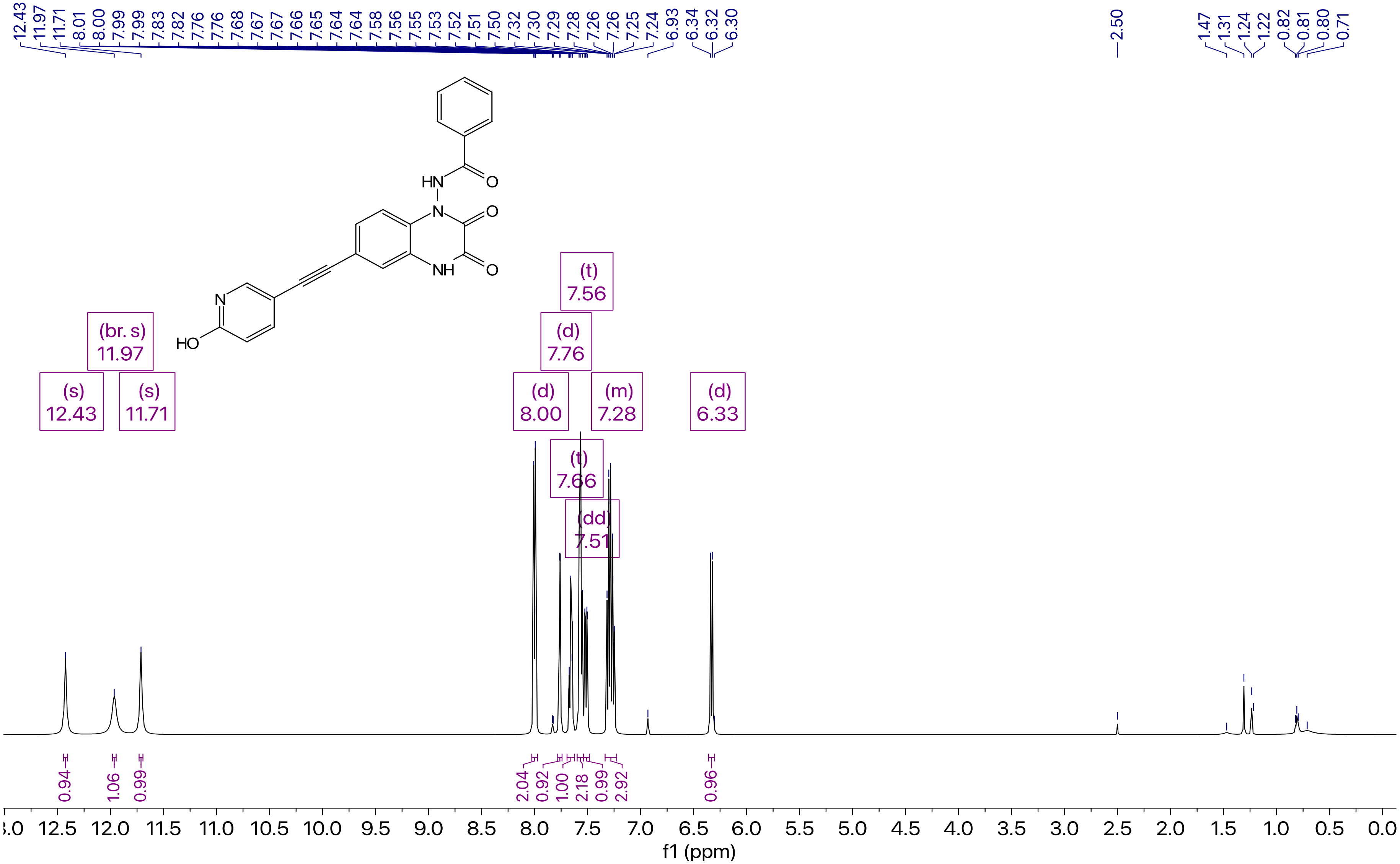

N-(6-((6-hydroxypyridin-3-yl)ethynyl)-2,3-dioxo-3,4-dihydroquinoxalin-1(2*H*)-yl)benzamide (**26**)

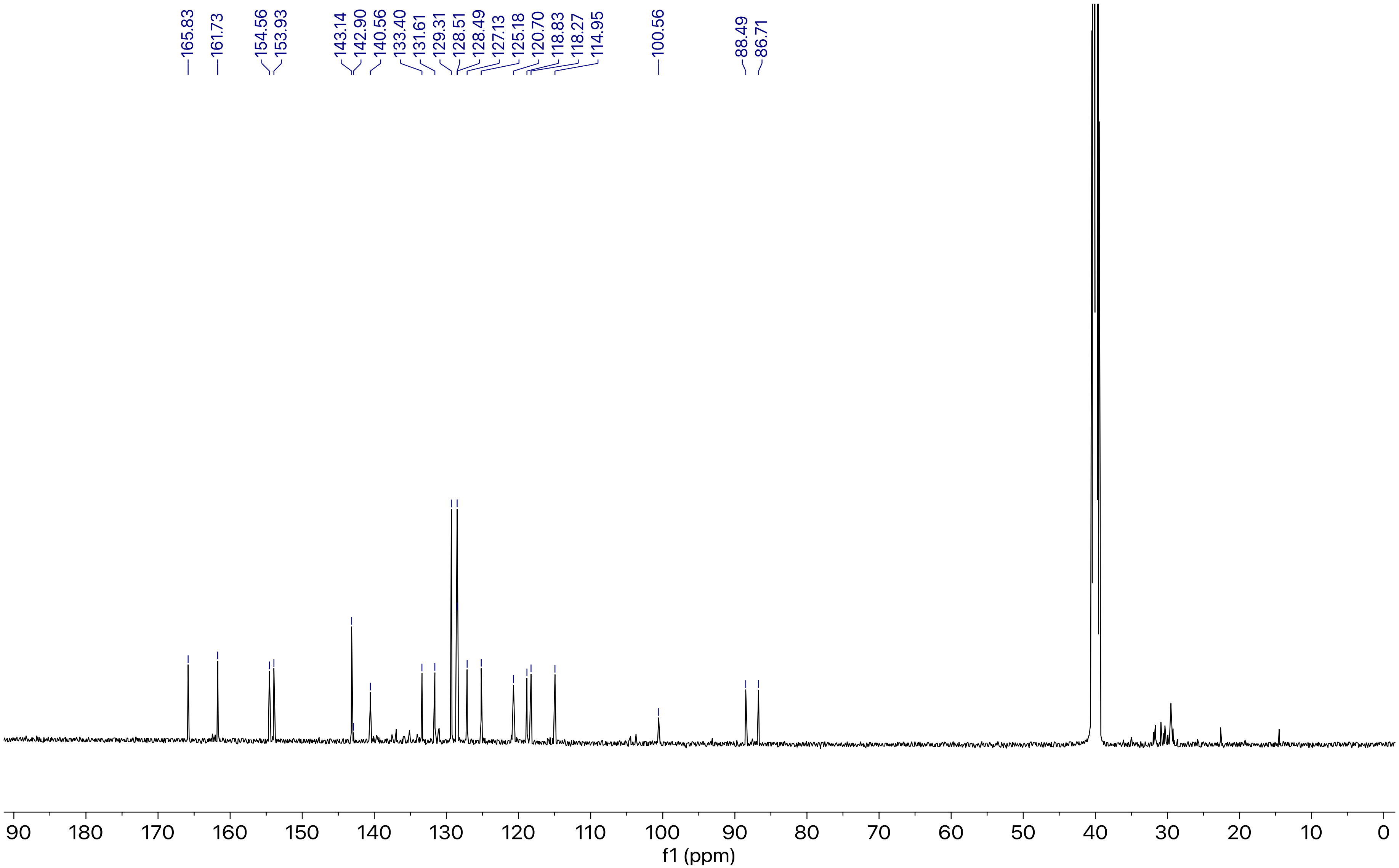

N-(2,3-dioxo-6-(pyrimidin-5-ylethynyl)-3,4-dihydroquinoxalin-1(2H)-yl)benzamide (**27**)

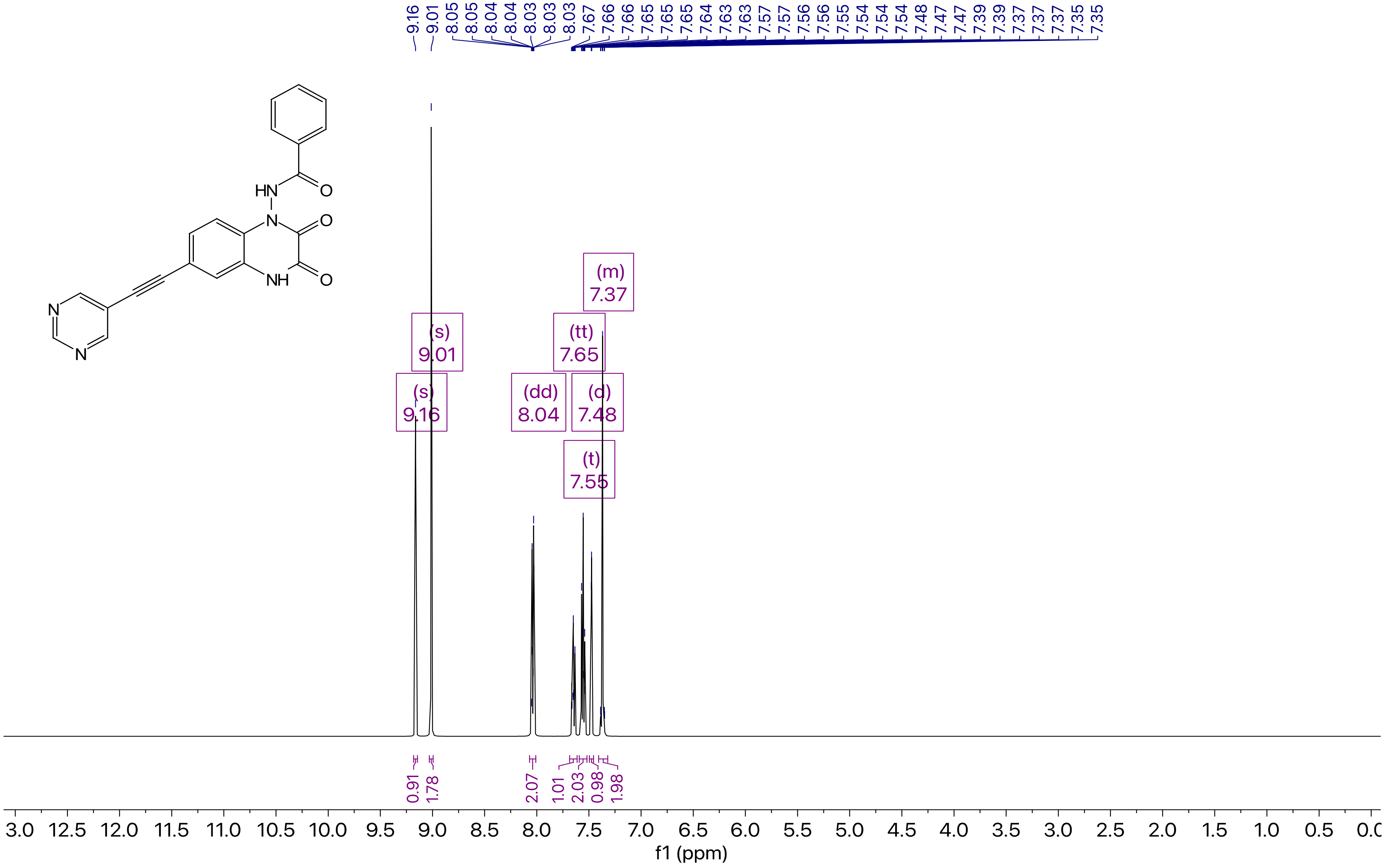

N-(2,3-dioxo-6-(pyrimidin-5-ylethynyl)-3,4-dihydroquinoxalin-1(2*H*)-yl)benzamide (**27**)

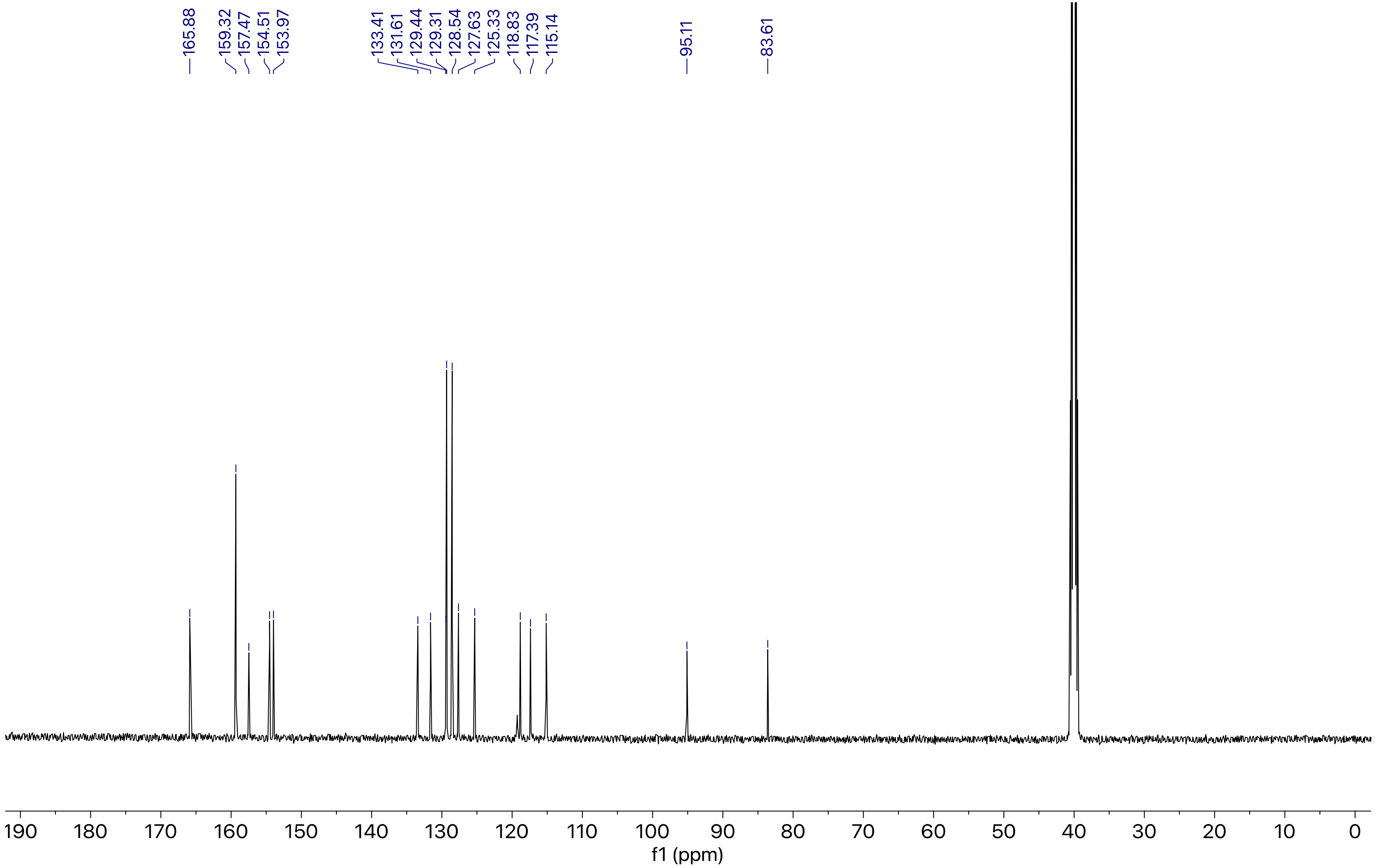

N-(6-(imidazo[1,2-b]pyridazin-3-ylethynyl)-2,3-dioxo-3,4-dihydroquinoxalin-1(2H)-yl)benzamide (**28**)

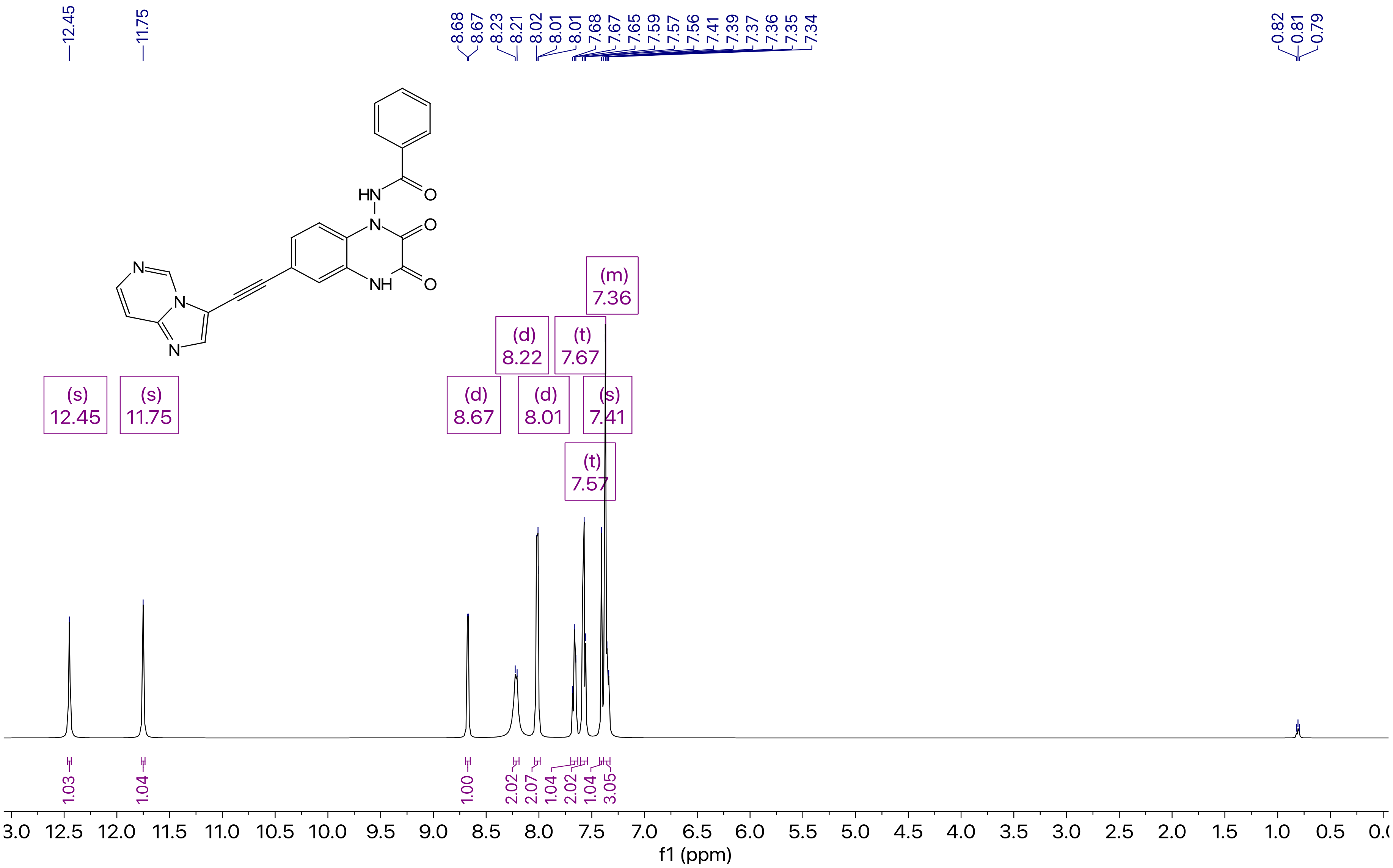

N-(6-(imidazo[1,2-b]pyridazin-3-ylethynyl)-2,3-dioxo-3,4-dihydroquinoxalin-1(2H)-yl)benzamide (**28**)

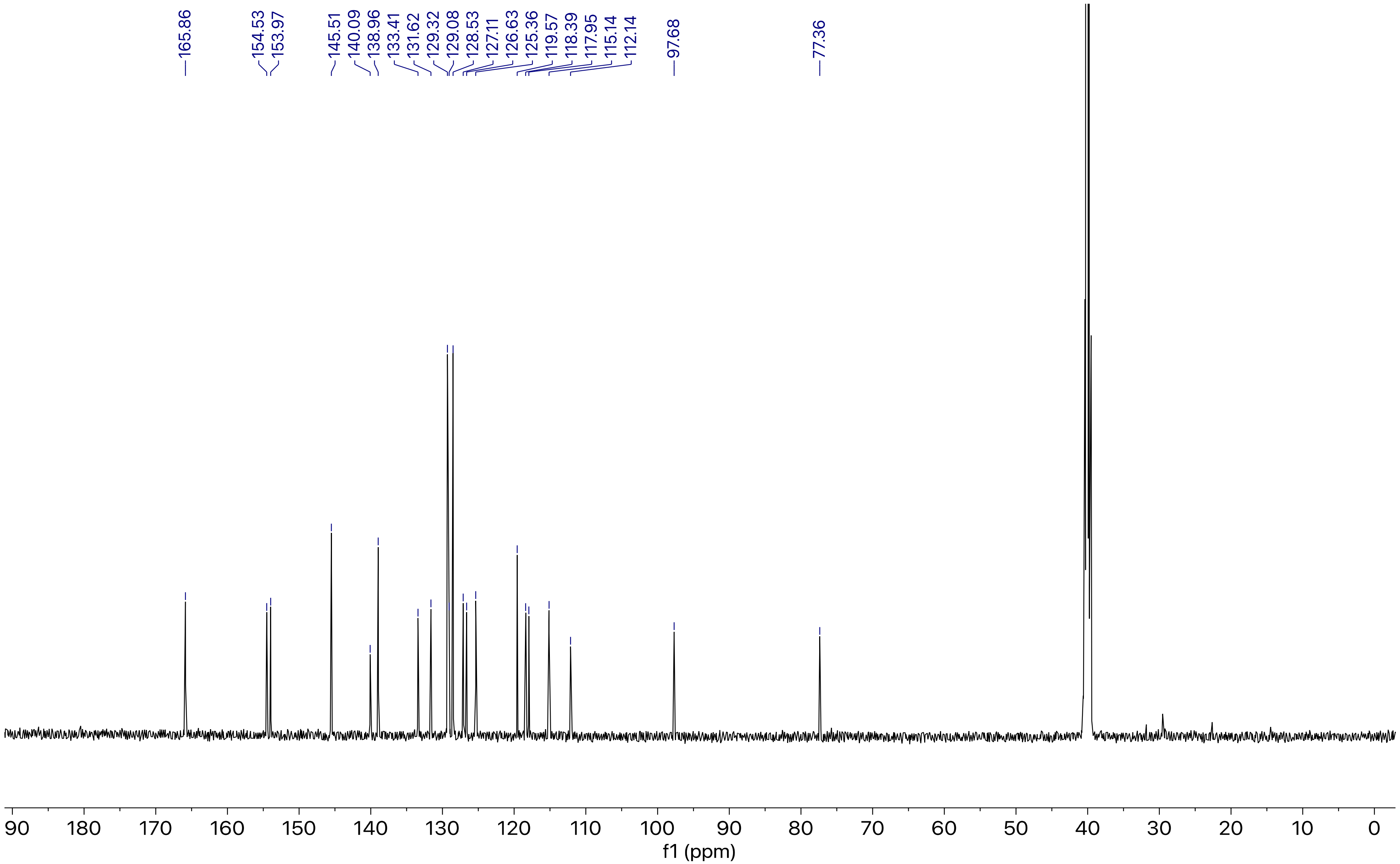

N-(6-((1H-pyrazolo[4,3-b]pyridin-5-yl)ethynyl)-2,3-dioxo-3,4-dihydroquinoxalin-1(2H)-yl)benzamide (**29**)

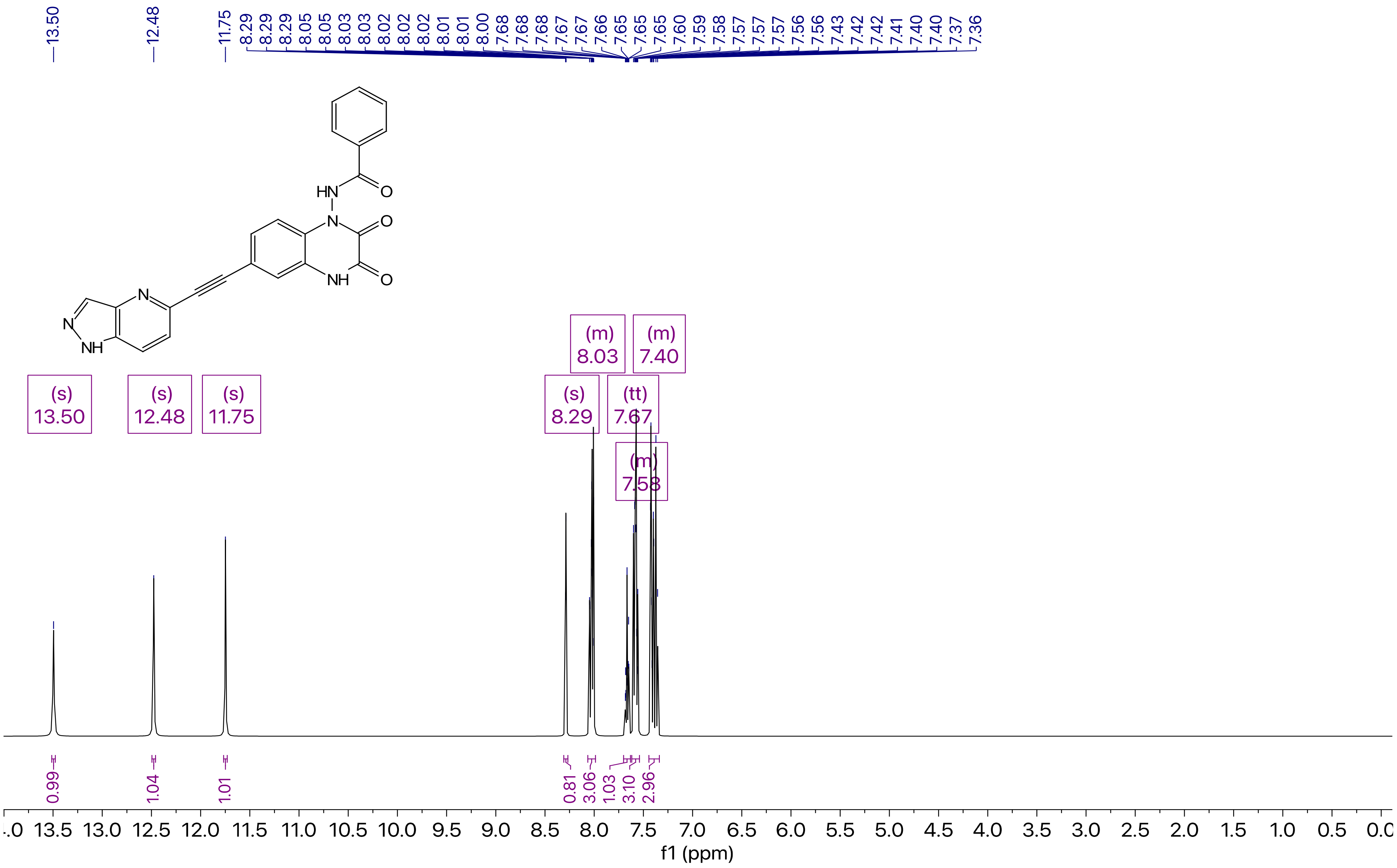

N-(6-((1H-pyrazolo[4,3-b]pyridin-5-yl)ethynyl)-2,3-dioxo-3,4-dihydroquinoxalin-1(2H)-yl)benzamide (**29**)

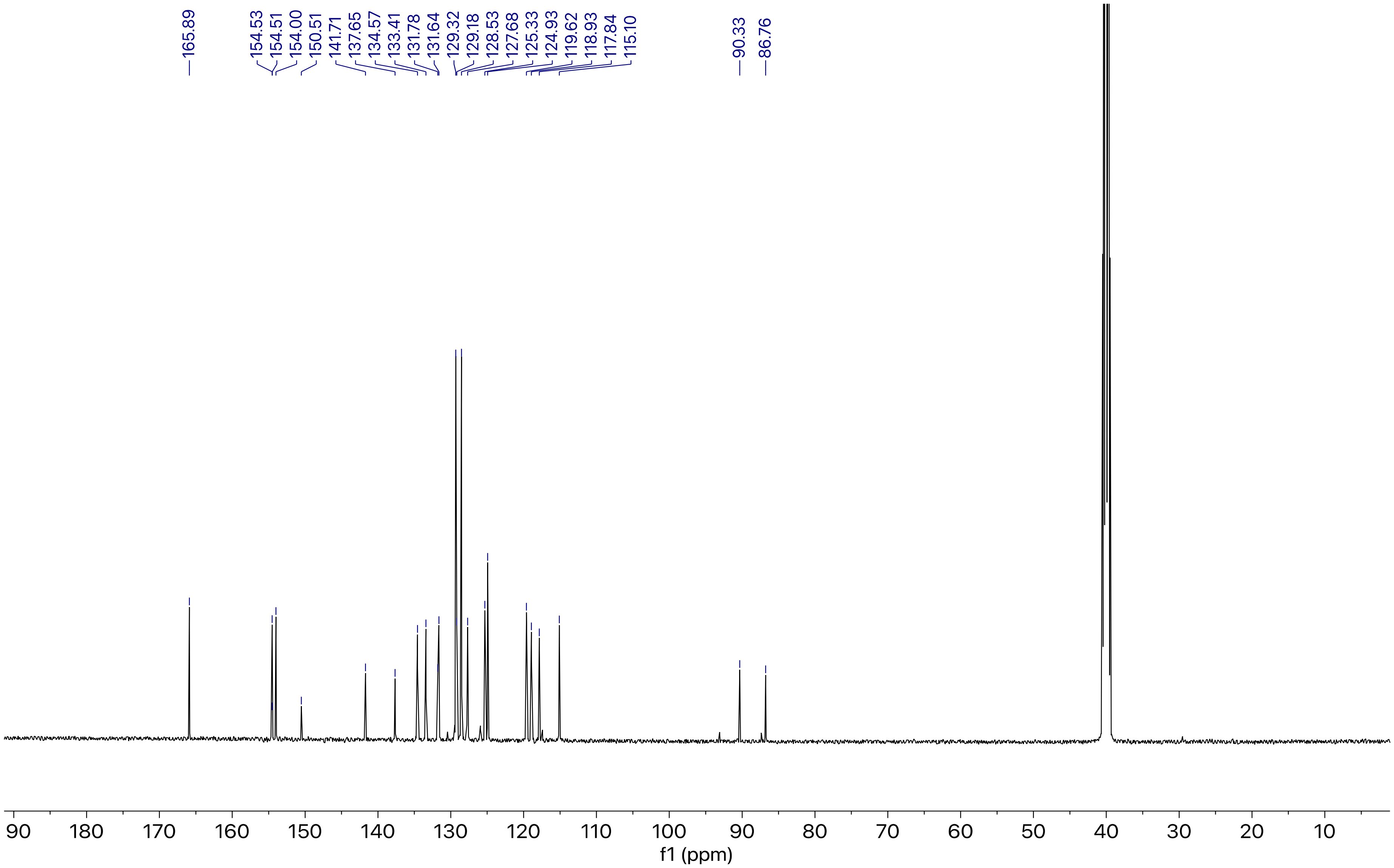

Supplement: Supplementary file 1 [file ijms-23-08797-s001.zip › ijms-1837131-supplementary.pdf]
